# Signaling pathway networks mined from human pituitary adenoma proteomics data

**DOI:** 10.1186/1755-8794-3-13

**Published:** 2010-04-28

**Authors:** Xianquan Zhan, Dominic M Desiderio

**Affiliations:** 1 Charles B. Stout Neuroscience Mass Spectrometry Laboratory, University of Tennessee Health Science Center, Memphis, Tennessee, USA; 2 Department of Neurology, University of Tennessee Health Science Center, Memphis, Tennessee, USA; 3 Clinical and Translational Science Institute, University of Tennessee Health Science Center, Memphis, Tennessee, USA; 4 Department of Molecular Science, University of Tennessee Health Science Center, Memphis, Tennessee, USA; 5 University of Tennessee Cancer Institute, University of Tennessee Health Science Center, Memphis, Tennessee, USA

## Abstract

**Background:**

We obtained a series of pituitary adenoma proteomic expression data, including protein-mapping data (111 proteins), comparative proteomic data (56 differentially expressed proteins), and nitroproteomic data (17 nitroproteins). There is a pressing need to clarify the significant signaling pathway networks that derive from those proteins in order to clarify and to better understand the molecular basis of pituitary adenoma pathogenesis and to discover biomarkers. Here, we describe the significant signaling pathway networks that were mined from human pituitary adenoma proteomic data with the Ingenuity pathway analysis system.

**Methods:**

The Ingenuity pathway analysis system was used to analyze signal pathway networks and canonical pathways from protein-mapping data, comparative proteomic data, adenoma nitroproteomic data, and control nitroproteomic data. A Fisher's exact test was used to test the statistical significance with a significance level of 0.05. Statistical significant results were rationalized within the pituitary adenoma biological system with literature-based bioinformatics analyses.

**Results:**

For the protein-mapping data, the top pathway networks were related to cancer, cell death, and lipid metabolism; the top canonical toxicity pathways included acute-phase response, oxidative-stress response, oxidative stress, and cell-cycle G2/M transition regulation. For the comparative proteomic data, top pathway networks were related to cancer, endocrine system development and function, and lipid metabolism; the top canonical toxicity pathways included mitochondrial dysfunction, oxidative phosphorylation, oxidative-stress response, and ERK/MAPK signaling. The nitroproteomic data from a pituitary adenoma were related to cancer, cell death, lipid metabolism, and reproductive system disease, and the top canonical toxicity pathways mainly related to p38 MAPK signaling and cell-cycle G2/M transition regulation. Nitroproteins from a pituitary control related to gene expression and cellular development, and no canonical toxicity pathways were identified.

**Conclusions:**

This pathway network analysis demonstrated that mitochondrial dysfunction, oxidative stress, cell-cycle dysregulation, and the MAPK-signaling abnormality are significantly associated with a pituitary adenoma. These pathway-network data provide new insights into the molecular mechanisms of human pituitary adenoma pathogenesis, and new clues for an in-depth investigation of pituitary adenoma and biomarker discovery.

## Background

Our long-term goals for this human pituitary study are to clarify the molecular mechanisms that are involved in pituitary adenoma pathogenesis and to discover tumor biomarkers. Towards those ends, a series of pituitary adenoma proteomic expression data, which include 111 proteins identified from a human pituitary non-functional adenoma tissue [1], 56 differentially expressed proteins (DEP's) from human pituitary nonfunctional adenoma tissues and from prolactinoma tissues [2,3], nine nitroproteins and three nitroprotein-protein complexes from a human pituitary nonfunctional adenoma tissue [4], and eight nitroproteins from a pituitary control tissue [5,6], were analyzed. There is a pressing need to clarify the significant signaling pathway networks that involve those pituitary adenoma proteins, DEP's, and nitroproteins in order to clarify and to better understand - on a molecular level - pituitary adenoma pathogenesis. Knowledge of significant signaling pathway networks will provide important clues and clear directions for an in-depth investigation of pituitary adenomas, for the discovery of tumor biomarkers, and for the development of efficacious therapeutic agents.

Over the past decades, high-throughput "-omic" technologies (genomics, transcriptomics, and proteomics) have been used in many fields, including biology and human diseases. Relative to the traditional molecular biology methods that had been used to study the role of a single gene, single protein, or single small-molecule model, those "-omic" data have driven the rapid development of systems biology to study a multiple-factor model of disease and to address the network of interaction and regulatory events that contribute to a disease. Pathway biology, as one important component of systems biology, has been extensively developed. Omic data-based pathway biology relies on an accurate and effective pathway analysis system. The Ingenuity Pathway Analysis (IPA) system is an extensively used (>1,300 peer-reviewed publications citing IPA; February 13, 2009) pathway analysis system that includes a large-scale knowledge base (~2.2 million scientific findings and 235 canonical pathways; February 13, 2009). IPA can identify statistically significant signaling pathway networks by analyzing the -omic data in those numerous canonical-pathway databases.

Proteomic data obtained from pituitary adenoma tissues [1-6] were analyzed with IPA software to define which well-characterized cell-signaling and metabolic pathways could be the most relevant during pituitary adenoma pathogenesis. This present study used the IPA system to reveal the significant signaling pathway networks that involve pituitary adenoma proteins, DEP's, and nitroproteins in an attempt to better understand the molecular mechanisms that are involved in pituitary adenoma pathogenesis, to discover potential biomarkers, and to develop efficacious therapeutic agents.

## Results

### Pathway networks derived from protein-mapping data

Among the 154 identifiers that represented the 111 proteins that were identified from human pituitary adenoma tissue, 147 identifiers were mapped to the corresponding molecules (genes; proteins), except for 7 identifiers (Additional file 1, **Table S1**). A total of 106 identifiers were eligible to proceed into pathway analysis after 41 duplicate identifiers were removed from those 147 mapped identifiers. Each identifier was annotated with a Swiss-Prot accession number, gene name, protein name, subcellular location, biofunction, and potential targets of drugs (Additional file 1, **Table S1**).

**Figure 1:**
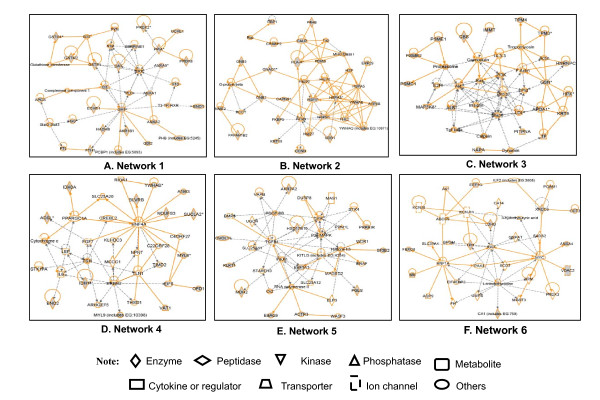
**Significant signaling pathway networks mined from pituitary adenoma protein-mapping dataset**. Significant signaling pathway networks that are involved in human pituitary adenoma mapping proteins and that function in (A) cancer, cell-to-cell signaling and interaction, small molecule biochemistry (Network 1); (B) post-translational modifications, protein-folding, hematological disease (Network 2); (C) organ morphology, reproductive-system development and function, molecular transport (Network 3); (D) lipid metabolism, molecular transport, smallmolecule biochemistry (Network 4); (E) cell death, hematological disease, cellular development (Network 5); and (F) lipid metabolism, small-molecule biochemistry, carbohydrate metabolism (Network 6). An orange solid edge denotes a direct relationship between two nodes (molecules: proteins; genes). A black unsolid edge denotes an indirect relationship between two nodes (molecules: proteins; genes). A gray node denotes an identified protein in our study [1]. The various shapes of nodes denote the different functions (Additional file 1, **Table S1**). A duplicated shape means this node contains multiple components. A curved line means intracellular translocation; A curved arrow means extracellular translocation.

The IPA analysis of those 106 network-eligible identifiers revealed 6 statistically significant pathway networks (Table 1 and Figure 1). Each network summarized in Table 1 includes all of the molecules (genes, proteins) that correspond to the nodes in Figure 1, the proteomics-identified molecules, and the statistical score.

**Table 1:** Signaling Pathway Networks that Involve Pituitary Adenoma Mapping Proteins.

Networks	Nodes (genes; proteins) in Network	Score	Nodes	Identified Nodes (Proteins)	Top Functions
1	AKR1B1, ANXA1, ANXA2, ANXA5, APCS, C3, CAT, Complement component 1, ECHS1, ENO1, ERK, F10, FGG, FTH1, FTL, FYN, GDI2, GH1, Glutathione transferase, GST, GSTM2, GSTO1, GSTP1, HADHB, Nfat, PCBP1 (includes EG:5093), PHB (includes EG:5245), PLC, PPIA, PRDX2, PRDX6, SERPINE1, Stat3-Stat3, T3-TR-RXR, UCHL1	58	35	27	Cancer, Cell-To-Cell Signaling and Interaction, Small Molecule-Biochemistry
2	ADCY, ATP5B, CALR, CAPNS1, CCNH, CRABP2, ERP29, FHL2, FKBP5, G-protein beta, GNAO1, GNB2, GNB3, HLA-A, HSF1, HSP, Hsp27, Hsp70, Hsp90, HSPA5, HSPA8, HSPB1, KRT19, MHC Class I, NFkB, NME2, P4HB, PAFAH1B2, PDIA3, Rbp, RBP1, SOD1, Tap, YWHAE, YWHAQ (includes EG:10971)	52	34	25	Post-Translational Modification, Protein Folding, Hematological Disease
3	14-3-3, Actin, Akt, ALB, APOA1, Calmodulin, Calpain, CBS, Dynamin, E2f, F Actin, GSN, HNRNPC, HPX, IMMT, Integrin, KRT9, LDL, MAP3K8, Mapk, NAPA, PI3K, PITPNA, Pkc(s), Pld, Proteasome, PSMC1, PSME1, PSME2, Ras, TF, Tgf beta, TPM3, TPM4, Tropomyosin	31	35	17	Organ Morphology, Reproductive System Development and Function, Molecular Transport
4	A1BG, ADSL, ARHGEF5, BLVRB, C22ORF28, C4ORF27, CREBL2, Cytochrome c, EIF6, ENO2, ERBB2, FGF7, FSH, HNF4A, IDH1, IDH3A, IL6, KLHDC3, LEP, MCCC1, MYL6, MYL9 (includes EG:10398), NDUFS3, NPNT, OFD1, PPARGC1A, RIOK1, SLC25A20, STK17A, SUCLA2, THSD1, TIMD2, TLN1, VAT1, YWHAB	22	35	13	Lipid Metabolism, Molecular Transport, Small-Molecule Biochemistry
5	ACTR3, AKR7A2, BMP3, Ck2, DUSP8, EBAG9, EEF1A1, ELP3, ERAF, Histone h3, HSD17B10, Insulin, Jnk, KITLG (includes EG:4254), KLK11, MAGED2, MAS1, MDH2, OVOL1, P38 MAPK, PDGF BB, PGLS, PPM1L, PRKRIR, RNA polymerase II, SF3B2, SLC25A11, SLC25A12, STARD10, STK4, TGFB1, UGDH, VAPB, WASF3, WDR1	18	36	11	Cell Death, Hematological Disease, Cellular Development
6	3-hydroxybutyric acid, ABCC9, AK1, ANXA4, AQP9, BPGM, CA14, CA1 (includes EG:759), CCT3, EEF1G, EIF4EBP2, FADS2, FBXO8, FH, GNPAT, HNF1A, ILF2 (includes EG:3608), INS1, KCNJ8, KCNJ11, L-triiodothyronine, LDHB, MGST3, MYC, PAX4, PGAM1, PRDX3, SCD2, SLC37A4, SRI, SRM, TPI1, UMPS, VDAC2, XRCC6	18	35	11	Lipid Metabolism, Small-Molecule Biochemistry, Carbohydrate Metabolism

Network 1 functions in cancer, cell-to-cell signaling and interaction, and small-molecule biochemistry (Figure 1A), and includes 35 nodes (genes; proteins); 27 of the proteins (77% of the total nodes) were identified with mass spectrometry (MS). GH1 and ERK play key roles in Network 1.

Network 2 functions in post-translational modifications, protein-folding, and hematological disease (Figure 1B), and includes 34 nodes (genes; proteins); 25 of the proteins (74% of the total nodes) were identified with MS. NF-kB, HSPA, and G-protein play key roles in Network 2.

Network 3 functions in organ morphology, reproductive-system development and function, and molecular transport (Figure 1C), and include 35 nodes (genes; proteins); 17 proteins (49% of the total nodes) were identified with MS. MAPK, Pkc, Ras, PI3K, Akt, and Calmodulin play key roles in Network 3.

Network 4 functions in lipid metabolism, molecular transport, and small-molecule biochemistry (Figure 1D), and includes 35 nodes (genes; proteins); 13 proteins (37% of the total nodes) were identified with MS. HNF4A, ERBB2, and FSH play key roles in Network 4.

Network 5 functions in cell death, hematological disease, and cellular development (Figure 1E), and includes 36 nodes (genes; proteins); 11 proteins (31% of the total nodes) were identified with MS. TGFB1, Jnk, P38 MAPK, and insulin play key roles in Network 5.

Network 6 functions in lipid metabolism, small-molecule biochemistry, and carbohydrate metabolism (Figure 1F), and includes 35 nodes (genes; proteins); 11 proteins (31% of the total nodes) were identified with MS. INS1, MYC, and HNF1A play key roles in Network 6.

**Figure 2:**
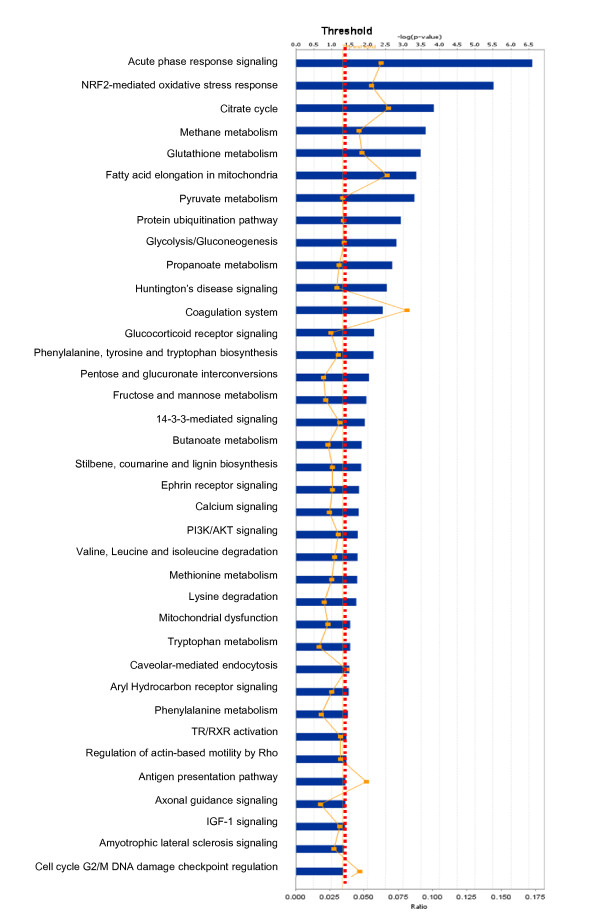
**Significant canonical pathways that are involved with pituitary adenoma protein-mapping data**.

**Figure 3:**
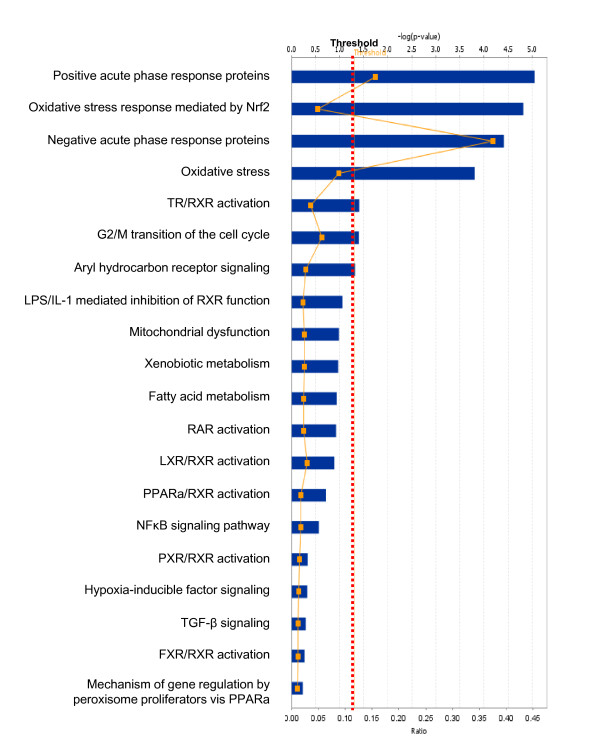
**Significant toxicological events that are involved with pituitary adenoma protein-mapping data**.

**Figure 4:**
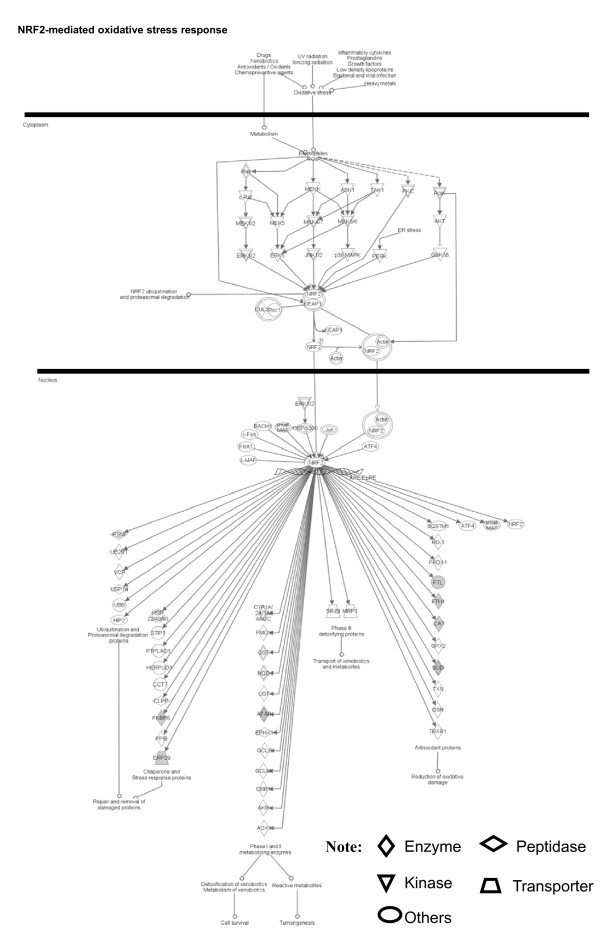
**NRF2-mediated oxidative-stress response pathway that is involved with pituitary adenoma protein-mapping data**. A gray label denotes an identified protein. The various shapes of nodes denote the different functions. A duplicated shape means this node contains multiple components. An arrow denotes the pathway direction. A line with a small circle denotes a biological result.

Among those pituitary adenoma protein-mapping data, a total of 37 statistically significant canonical pathways were identified that involve the identified proteins (Figure 2). The top ten canonical pathways include acute-phase response signaling, NRF2-medicated oxidative-stress response, citrate cycle, methane metabolism, glutathione metabolism, fatty-acid elongation in mitochondria, pyruvate metabolism, the protein ubiquitination pathway, glycolysis/gluconeogenesis, and propanoate metabolism. Seven statistically significant toxicity pathways out of a total of 20 were mined from those mapping proteomic data, and include positive acute-phase response proteins, oxidative stress-response mediated by Nrf2, negative acute-phase response proteins, oxidative stress, TR/RXR activation, cell-cycle G2/M transition, and aryl hydrocarbon receptor signaling (Figure 3). The identified proteins in the linkage of each canonical pathway are labeled (Figure 4; Additional file 2, **Figure S1**).

Figure 4 shows the scheme of a representative canonical pathway - the NRF2-mediated oxidative stress response. The extracellular oxidative stress-related factors induce intracellular electophile ROS formation to activate the NRF2 via MAPK-signaling pathways such as the Ras/Raf-ERK, JNK, p38MAPK, and PI3K/AKT pathways. The activated NRF2 is translocated into the nucleus to initiate antioxidative gene/protein expression such as antioxidant proteins (SOD, GPX2, CAT, FTH1, FTL, etc.) to reduce oxidative damage, chaperone/stress-response proteins, and ubiquitination and proteasonal degradation proteins to repair and remove damaged proteins. Also, NRF2 involves the regulation of expression of detoxifying proteins for cell survival. However, the dysregulation of this NFR2 pathway will cause the formation of more reactive metabolites, which in turn could promote tumorigenesis. Some components in this pathway have been identified with our proteomics study, and include FTL, FTH1, CAT, SOD, AFAR, FKBP5, and ERP29. The detailed components of the other canonical pathways that are derived from the pituitary adenoma protein-mapping data are shown in Additional file 2, Figures S1.1-S1.36.

### Pathway networks derived from comparative proteomics data

Among the 86 identifiers that represent the 56 DEP's that were identified from human pituitary adenoma tissues, 75 identifiers were mapped to the corresponding molecules (genes; proteins) except for 11 identifiers (Additional file 1, **Table S2**). The 75 identifiers are significant because they derive from a comparison of adenomas and controls. A total of 47 identifiers were eligible to proceed into pathway analysis after 28 duplicate identifiers were removed from those 75 mapped identifiers. Each identifier was annotated with a Swiss-Prot accession number, gene name, fold-change, protein name, subcellular location, biofunction, and potential targets of drugs (Additional file 1, **Table S2**).

**Figure 5:**
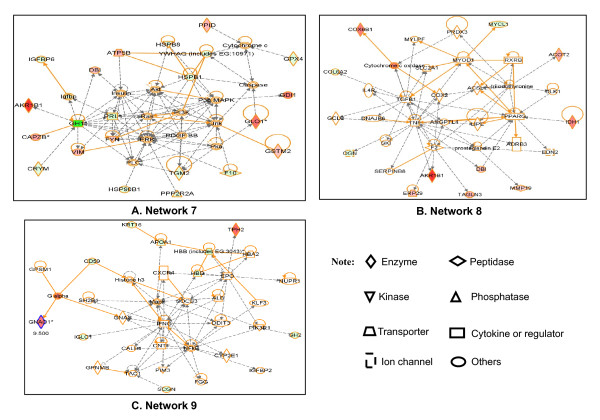
**Significant signaling pathway networks mined from pituitary adenoma comparative dataset**. Significant signaling pathway networks that are involved in human pituitary adenoma differentially expressed proteins and that function in (A) cancer, endocrine-system development and function, organ morphology (Network 7); (B) lipid metabolism, molecular transport, smallmolecule biochemistry (Network 8); and (C) hematological-system development and function, tissue morphology, hematological disease (Network 9). An orange solid edge denotes a direct relationship between two nodes (molecules: proteins; genes). A black unsolid edge denotes an indirect relationship between two nodes (molecules: proteins; genes). A red node denotes an up-regulated protein, and a green node denotes a down-regulated protein in our studies [2,3], with different color levels that reflect the fold-change of protein differential expression (Additional file 1, **Table S2**). The various shapes of nodes denote the different functions (Additional file 1, **Table S2**). A duplicated shape means this node contains multiple components. A curved line means intracellular translocation; A curved arrow means extracellular translocation.

The IPA analysis of those 47 network-eligible identifiers revealed three statistically significant pathway networks (Table 2 and Figure 5). Each network summarized in Table 2 includes all of those molecules (genes; proteins) that correspond to the nodes in Figure 5 the DEP's, and the statistical score.

**Table 2:** Signaling Pathway Networks that Involve Human Pituitary Adenoma Differentially Expressed Proteins (DEPs).

Networks	Nodes (genes; proteins) in Network	Score	Nodes	Identified Nodes (DEPs)	Top Functions
7	AKR1B1, Akt, ATP5B, CAPZB, Caspase, CRYM, Cytochrome c, DBI, ERK, F10, FYN, GDI1, GH1, GLO1, GPX4, GSTM2, HSP90B1, HSPB1, HSPB8, Igfbp, IGFBP6, Insulin, Jnk, P38 MAPK, PDGF BB, PI3K, Pka, PLC, PPID, PPP2R2A, PRL, Ras, TGM2, VIM, YWHAQ (includes EG:10971)	53	35	22	Cancer, Endocrine System Development and Function, Organ Morphology
8	ACOT2, ACSL1, ADRB3, AKR1B1, ANGPTL4, COL6A2, COX2, COX6B1, Cytochrome c oxidase, DBI, DLK1, DNAJB6, EDN2, ERP29, F2, GCLC, IDH1, IL4R, L-triiodothyronine, LIPE, MMP19, MYCL1, MYLPF, MYOD1, OGN, PPARG, PRDX3, prostaglandin E2, RXRB, SERPINB8, SKI, SLC2A1, TAGLN3, TGFB1, TNF	22	35	11	Lipid Metabolism, Molecular Transport, Small Molecule Biochemistry
9	ALB, APOA1, CALB1, CD59, CNTF, CXCR4, CYP2E1, DDIT3, EPO, FGG, G alpha, GH2, GNAO1, GNAS, GPNMB, GPSM1, HBA2, HBB (includes EG:3043), HBD, Histone h3, IFNG, IGFBP2, IGLC1, KLF3, KRT16, Mapk, NFkB, NUPR1, PIK3R1, PIM3, SCGN, SH2B1, SOCS3, TAC1, TPH2	19	35	10	Hematological System Development and Function, Tissue Morphology, Hematological Disease

Network 7 functions in cancer, endocrine-system development and function, and organ morphology (Figure 5A) and includes 35 nodes (genes; proteins); among those 35 nodes, 22 DEPs (63% of the total nodes) were identified with MS. GH1, ERK, P38 MAPK, PRL, Insulin, Akt, Ras, and Jnk play key roles in Network 7.

Network 8 functions in lipid metabolism, molecular transport, and small-molecule biochemistry (Figure 5B), and include 35 nodes (genes; proteins); 11 DEP's (31% of the total nodes) were identified with MS. TGFB1, TNF, PPARG, and MYOD1 play key roles in Network 8.

Network 9 functions in tissue morphology, and hematological-system development, function, and disease (Figure 5C), and include 35 nodes (genes; proteins); 10 DEP's (29% of the total nodes) were identified with MS. MAPK, IFNG, NFkB, and EPO play key roles in Network 9.

**Figure 6:**
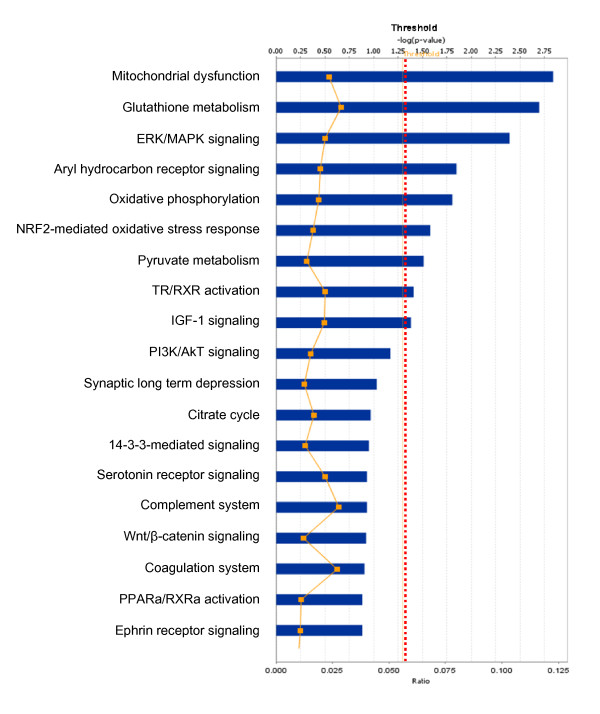
**Significant canonical pathways that are involved with pituitary adenoma comparative-proteomic data**.

**Figure 7:**
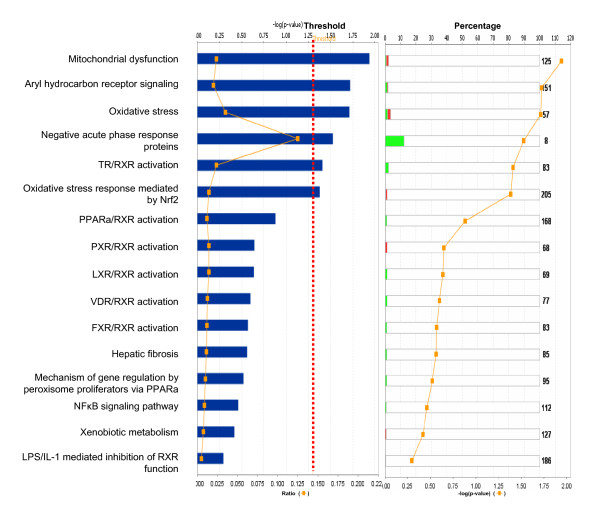
**Significant toxicological events that are involved with pituitary adenoma comparative-proteomic data**. Green bar = downregulated, red bar = upregulated, grey bar = no change, white bar = no overlap with dataset.

**Figure 8:**
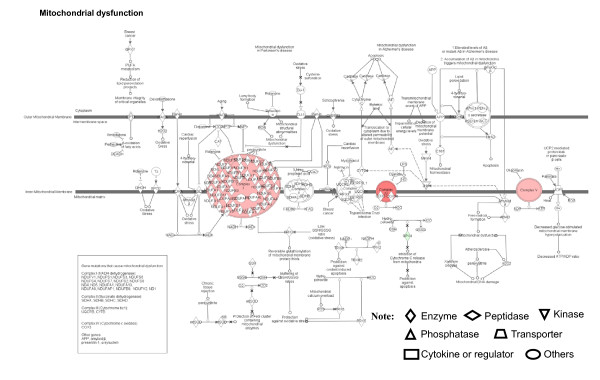
**Mitochondrial dysfunctional pathway that is involved with pituitary adenoma comparative-proteomic data**. Red label = upregulated, green label = down regulated. The various shapes of nodes denote the different functions. A duplicated shape means this node contains multiple components. An arrow denotes the pathway direction. A line with a small circle denotes a biological result.

**Figure 9:**
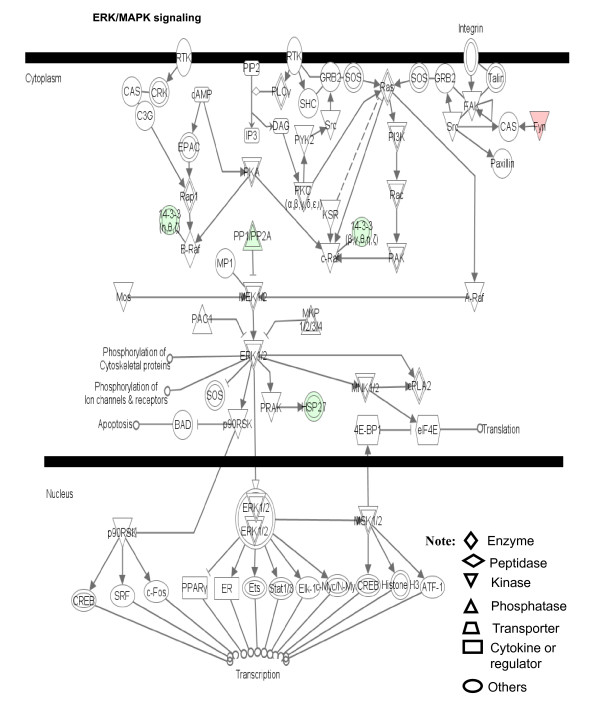
**ERK/MAPK signaling pathway that is involved with pituitary adenoma comparative-proteomic data**. Red label = upregulated, green label = downregulated. The various shapes of nodes denote the different functions. A duplicated shape means this node contains multiple components. An arrow denotes the pathway direction. A line with a small circle denotes a biological result.

Among those pituitary adenoma comparative proteomic data, a total of nine statistically significant canonical pathways out of 19 pathways were identified that involve those DEP's (Figure 6). The top nine canonical pathways include mitochondrial dysfunction, glutathione metabolism, ERK/MAPK signaling, aryl hydrocarbon-receptor signaling, oxidative phosphorylation, NRF2-mediated oxidative-stress response, pyruvate metabolism, TR/RXR activation, and IGF-1 signaling. Six statistically significant toxicity pathways out of a total of 16 were mined from those comparative proteomic data, and include mitochondrial dysfunction, aryl hydrocarbon-receptor signaling, oxidative stress, negative acute-phase response proteins, TR/RXR activation, and oxidative-stress response mediated by Nrf2 (Figure 7). The identified proteins in the linkage of each canonical pathway are labeled (Figures 8 and 9; and Additional file 2, **Figure S2**).

Figure 8 shows, as an example, the complicated mitochondrial dysfunctional pathway. The mitochondrial dysfunctional pathway was distributed within five regions of a cell - cytoplasm, outer mitochondrial membrane, intermembrane space, inner mitochondrial membrane, and mitochondrial matrix. The mitochondrial complexes I - V locate in the inner mitochondrial membrane. Gene mutations that cause mitochondrial dysfunction include Complex I (NADH dehydrogenesase) (NDUFV1, NDUFV2, NDUFS1, NDUFS2, NDUFS3, NDUFS6, NDUFS7, NDUFS8, ND1, ND4, ND5, NDUFA1, NDUFA8, NDUF13, NDUFAF1, and NDUFB9), Complex II (succinate dehydrogenase) (SDHA, SDHB, SDHC, and SDHD), Complex III (cytochrome bc 1) (UQCRB, CYTB), Complex IV (cytochrome c oxidase) [COX3 (cyclooxygenase 3)], and other genes (APP, amyloid β, presenin-1, and α-synuclein). Our comparative proteomics data demonstrate that some components of mitochondrial complexes are significantly up-regulated in pituitary adenomas relative to controls; for example, ATP5B (ATP synthase, H^+^-transporting, mitochondrial F1 complex, beta polypeptide; 5-fold), COX6B1 (cytochrome c oxidase subunit Vib polypeptide 1; 9-fold), and NDUFS8 (NADH dehydrogenase ubiquinone Fe-S protein 8; 5-fold). Moreover, GPX4 (glutathione peroxidase 4) was significantly down-regulated (26-fold) in pituitary adenomas relative to controls. GPX4 plays important roles in the cytochrome c - apoptosis pathway.

Figure 9 shows the ERK/MAPK signaling pathway. The extracellular signals are transducted into the cytoplasm via the receptor tyrosine kinase (RTK) and the integrin receptor to activate the Ras/Raf pathway. Ras is regulated by PKC and SOS that is regulated by Src and FYN signals. Raf includes three subfamilies A, B, and C. A-Raf is activated by the Ras signal. B-Raf is activated by Rap1, PKA, and 14-3-3 signals. C-Raf is activated by Ras, PKA, and 14-3-3 signals. ERK1/2 are activated by Rafs-MEK1/2 signals. The activated ERK1/2's will perform their biological roles in the cytoplasm such as phosphorylation of cytoskeletal proteins, ion channels and receptors, and regulation of apoptosis and translation; or will translocate into the nucleus to regulate the transcription of multiple genes such as Stat1/3, Myc, CREB, histone H3, etc. Compared to human pituitary controls, in pituitary adenomas, the FYN (FYN oncogene related to SRC, FGR, YES) was up-regulated (4-fold), 14-3-3 protein down-regulated (44-fold), HSPB1 (heat shock 27 kDa protein 1) down-regulated (5-fold), and PPP2R2A (protein phosphatase 2 regulatory subunit B alpha isoform) down-regulated (8-fold), within the ERK/MAPK signaling pathway system. Also, the PKA regulatory subunit type I beta was nitrated (Additional file 1, **Table S3**) to most probably interfere with PKA functions; that nitration suggests that oxidative/nitrative stress signals are also involved in the regulation of the ERK/MAPK signaling system. The detailed components of the other canonical pathways that are derived from pituitary adenoma comparative proteomic data are shown in the Additional file 2, Figures S2.1-S2.7.

### Pathway networks derived from nitroproteomic data

A total of 12 identifiers that represent nine nitroproteins, and three non-nitrated proteins, from a human pituitary adenoma tissue were mapped to their corresponding genes/proteins (Additional file 1, **Table S3**). A total of 10 identifiers were eligible to proceed into pathway analysis, except for LILRA4 and ZNF432. Each identifier was annotated with a Swiss-Prot accession number, gene name, nitration status, protein name, subcellular location, biofunction, and potential targets of drugs (Additional file 1, **Table S3**). Twelve identifiers that represent nine nitroproteins from human pituitary control tissue were also mapped to nine genes. Nine network-eligible identifiers proceeded to pathway analysis (Additional file 1, **Table S4**). Nitration usually decreases the activity of a protein.

**Figure 10:**
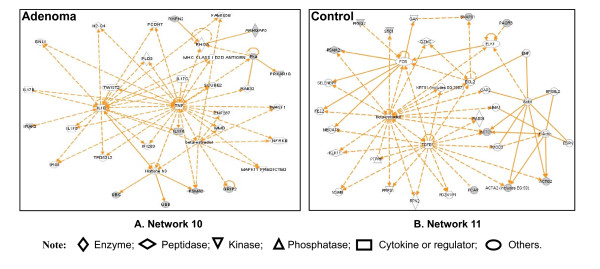
**Significant signaling pathway networks mined from nitroproteomic dataset**. Significant signaling pathway networks that are involved in human pituitary adenoma nitroproteomic data. (A) Network 10 is derived from adenoma nitroproteomic data and function in cancer, cell cycle, reproductive-system disease. A gray node denotes an identified nitroprotein or protein that interact with nitroproteins in our study [4]. (B) Network 11 is derived from control nitroproteomic data and function in gene expression, cellular development, connective tissue development and function. A gray node denotes an identified nitroprotein in our studies [5,6]. An orange solid edge denotes a direct relationship between two nodes (molecules: proteins; genes). An orange unsolid edge denotes an indirect relationship between two nodes (molecules: proteins; genes). The various shapes of nodes denote the different functions (Additional file 1, Tables S3 and S4). A curved line means intracellular translocation; a curved arrow means extracellular translocation.

The IPA analysis of those 10 network-eligible identifiers from human pituitary adenoma tissue revealed one statistically significant pathway network (Table 3 and Figure 10A). That network (Table 3) includes all molecules (genes; proteins) that correspond to the nodes in Figure 10A, the MS-identified nitroproteins, and the statistical score. Network 10 functions in cancer, cell cycle, and reproductive-system disease (Figure 10A), and includes 35 nodes (genes; proteins); nine nitroproteins (26% of the total nodes) were identified with MS. TNF, IL1B, and beta-estradiol play key roles in Network 10. For those nine network-eligible identifiers from human pituitary control tissue, the IPA analysis revealed one statistically significant pathway network (Network 11) that functions in gene expression, cellular development, and connective-tissue development and function (Table 3 and Figure 10B). Network 11 includes 35 nodes (genes; proteins); and nine nitroproteins (26% of the total nodes) were identified with MS. TGFB1, FOS, and beta-estradiol play key roles in Network 11.

**Table 3:** Signaling Pathway Networks that Involve Nitroproteomic Data From Pituitary Adenoma and Control.

Networks	Nodes (genes; proteins) in Network	Score	Nodes	Identified Nodes (Nitroproteins)	Top Functions
10 (Adenoma)	ARHGAP5, beta-estradiol, FAM105B, GNL1, GRIP2, H2-Q4, Histone h3, IFI203, IL17B, IL17C, IL1B, IL1F5, IL1F6, IRAK2, IRG1, MAPK11 PREDICTED, MHC CLASS I D2D ANTIGEN, MMD, NFRKB, PCDH7, Pka, PLD3, PRKAR1B, PSMA2, RAB32, RHOA, RHPN2, SCUBE2, TM4SF1, TNF, TPD52L2, TWIST2, UBB, UBC, ZNF267	24	35	9	Cancer, Cell Cycle, Reproductive System Disease
11 (Control)	ACTA2 (includes EG:59), ACTC1, ACTG2, Actin, BCL2, beta-estradiol, BMF, CAP2, ELK1, EPS8L2, ESPN, F Actin, FCAR, FEZ2, FOS, GAK, GZMC, KLK11, KRT81 (includes EG:3887), LIMA1, MBOAT5, MSMB, PAQR3, PDZK1IP1, PRKG2, PRPS1, PSMA2, PTPRK, RAB31, RPN2, SELENBP1, SNAP91, STC1, TGFB1, TMOD3	26	35	9	Gene Expression, Cellular Development, Connective Tissue Development and Function

**Figure 11:**
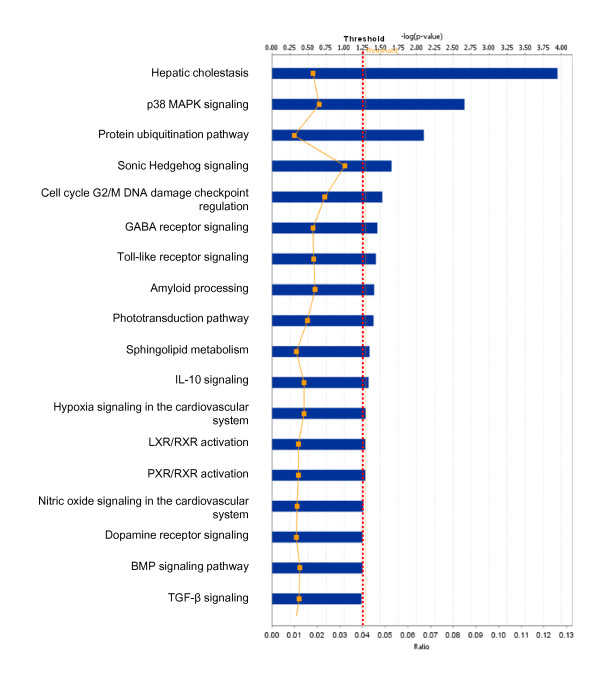
**Significant canonical pathways that are involved with pituitary adenoma nitroproteins**.

**Figure 12:**
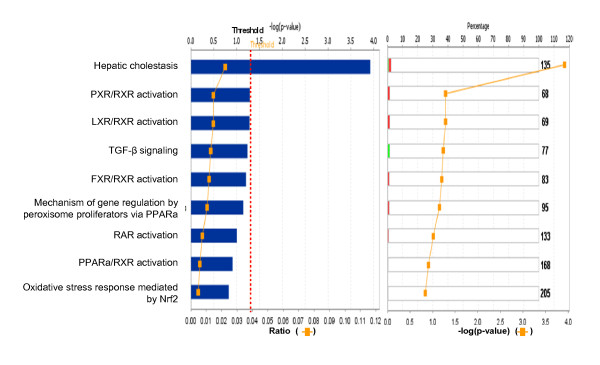
**Significant toxicological events that are involved with pituitary adenoma nitroproteins**. Red bar = nitrated proteins, green bar = unnitrated proteins, grey bar = no change, white bar = no overlap with dataset.

**Figure 13:**
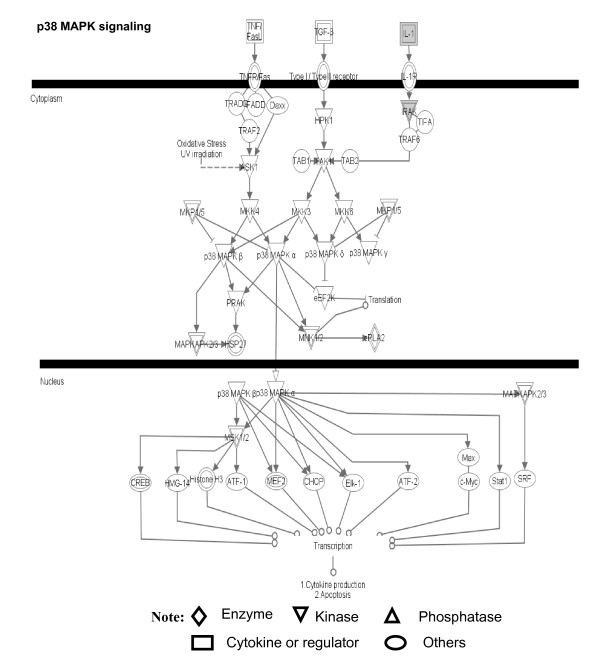
**p38 MAPK signaling pathway that is involved with pituitary adenoma nitroproteins**. A gray label denotes an identified protein. The various shapes of nodes denote the different functions. A duplicated shape means this node contains multiple components. An arrow denotes the pathway direction. A line with a small circle denotes a biological result.

Among those pituitary adenoma qualitative nitroproteomic data, a total of 12 statistically significant canonical pathways were identified that involve nitroproteins (Figure 11). The top canonical pathways include hepatic cholestasis, p38 MAPK signaling, the protein-ubiquitination pathway, sonic-hedgehog signaling, cell-cycle G2/M DNA damage-checkpoint regulation, GABA-receptor signaling, Toll-like receptor signaling, amyloid processing, the phototransduction pathway, sphingolipid metabolism, IL-10 signaling, hypoxia signaling, LXR/RXR activation, and PXR/RXR activation. Three statistically significant toxicity pathways were mined, and include hepatic cholestasis, PXR/RXR activation, and LXR/RXR activation (Figure 12). The identified nitroproteins in the linkage of each canonical pathway are labeled (Figures 13; and Additional file 2, **Figure S3**).

Figure 13 shows the representative p38 MAPK signaling pathway. The extracellular inflammatory cytokine signals (such as TNF, TGF-β, and IL-1) are transducted into the cytoplasm via a binding to their corresponding receptors in the membrane to activate ASK1 and TAK1. p38 MAPK's include four subfamilies (α, β, γ, δ) that are activated by ASK1/MKK4, TAK1/MKK3/6, and MKP1/5 signals. The activated p38 MAPKs are translocated into the nucleus to activate the transcription of multiple genes such as CREB, c-Myc, Stat1, histone H3, Elk-1, etc., and to regulate apoptosis. Our pituitary adenoma nitroproteomic study has discovered the IL1-IL1R-IRAK2 complex in a human pituitary adenoma tissue; IL-1 was nitrated, and IRAK2 (interleukin-1 receptor-interacting protein 2) was identified to associate with IL1R.

Additional file 2, **Figure S3.4 **shows the cell-cycle G2/M DNA damage checkpoint-regulation pathway. p53 and cdc25 B/C play important roles in this oxidative damage-induced pathway. The 14-3-3 proteins (down-regulated 44-fold in pituitary adenomas compared to controls; Additional file 1, **Table S2**) are the important regulators in this pathway - they couple with Cdc25 B/C to participate in the nuclear export of Cdc25, and couple with Cdc2 and Cyclin B to participate in the cytoplasmic sequestration of cdc2 and cyclin B. The detailed components of the other canonical pathways that are derived from the pituitary adenoma nitroproteomic data are shown in Additional file 2, Figures S3.1-S3.10.

**Figure 14:**
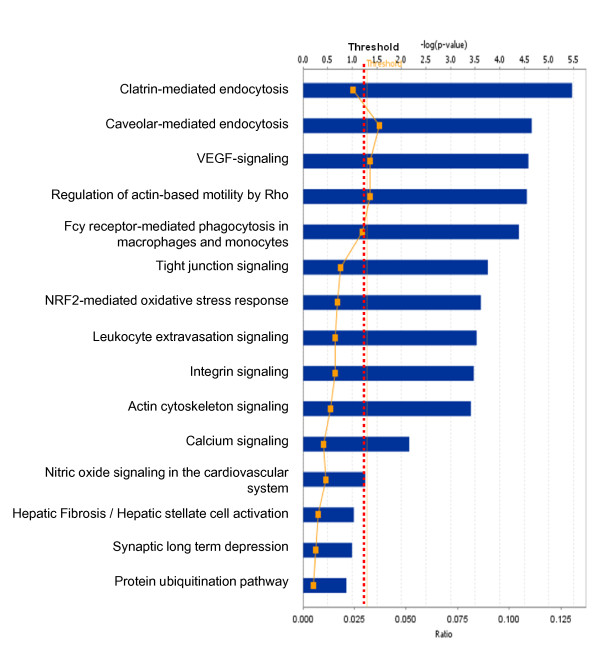
**Significant canonical pathways that are involved with control pituitary nitroproteins**.

Among those control pituitary adenoma qualitative nitroproteomic data, a total of 12 statistically significant canonical pathways were identified that involve nitroproteins (Figure 14), and include clatrin-mediated endocytosis, caveolar-mediated endocytosis, VEGF signaling, regulation of actin-based motility by Rho, Fcy receptor-mediated phagocytosis in macrophages and monocytes, tight-junction signaling, NRF2-mediated oxidative-stress response, leukocyte extravasation signaling, integrin signaling, actin-cytoskeleton signaling, and calcium signaling. No statistically significant toxicity pathways were mined. The identified nitroproteins in the linkage of each canonical pathway are labeled (Additional file 2, **Figure S4**). The detailed components of the other canonical pathways that are derived from pituitary control nitroproteomic data are shown in Additional file 2, Figures S4.1-S4.12.

## Discussion

The present study, for the first time, used bioinformatics pathway analysis to reveal the significant signaling pathways and networks that are associated with pituitary adenomas; three types of proteomic data were used - pituitary adenoma protein-mapping [1], comparative proteomic [2,3], and nitroproteomic [4-6]. Protein-mapping data were obtained with a 2DGE-arrayed pituitary adenoma proteome, followed by MS characterization of the proteins. The protein-mapping data-derived pathways and networks could reflect each potential pathway network that exists in a human pituitary adenoma proteome, and that associates with its pathophysiology. Those pathway networks are the baseline for the discovery of adenoma-related pathway networks. Comparative proteomic data were obtained from 2DGE-arrayed adenoma and control proteome images, followed by MS characterization of DEP's. Those DEP data-derived pathway networks will reflect significant adenoma-related pathway networks. Nitroproteomic data include those endogenous proteins that were nitrated at a tyrosine residue. Tyrosine nitration, a chemically stable marker of oxidative stress, alters protein function, and is extensively associated with tumor inflammation and neurodegenerative disease. The nitroproteomic data-derived pathway networks will directly reflect the pathways of oxidative damage that contribute to the formation of a pituitary adenoma. Among the complicated pathway networks described above, several signaling pathways and networks were found to be significantly associated with a pituitary adenoma, and include mitochondria dysfunction, oxidative stress, cell-cycle dysregulation, and the MAPK-signaling system. Those four systems will be described below.

### Mitochondria dysfunction

Mitochondria are dynamic intracellular organelles that are essential for cellular life, death, and differentiation, and play a central role in oxidative metabolism and apoptosis. Mitochondrial dysfunctions underlie a broad spectrum of human diseases [7,8] that include cancer [9,10], neurodegenerative diseases [11], cardiovascular diseases [12], diabetes mellitus [13], and inflammatory diseases [14,15]. Notable differences in the structure and function of mitochondria appear between cancer and normal cells, and include differences in mtDNA sequence, molecular composition, and metabolic activity [9,10]. Mitochondria involve multiple metabolic functions that include oxidative phosphorylation - an energy-generating process that couples the oxidation of respiratory substances to the synthesis of ATP, oxidative decarboxylation of pyruvate, the tricarboxylic acid cycle, fatty-acid oxidation, glycolysis, intracellular homeostasis of inorganic ions such as calcium and phosphate, and intracellular apoptosis [10]. Mitochondrial dysfunction in cancer includes an increased gluconeogenesis, reduced pyruvate oxidation and increased lactic acid production, increased glutaminolytic activity, and reduced fatty-acid oxidation. The activity of certain mitochondrial enzymes that are integral to the process of oxidative phosphorylation is decreased in cancer compared to normal cells; those enzymes include ATPase, cytochrome c oxidase, and adenine nucleotide translocase. The additional alterations of gene expression between cancer and normal cells include the anti-apoptotic oncogenes that encode Bcl-2 and Bcl-XL, and genes that encode the peripheral benzodiazepin receptor (PBR), the PBR-associated protein Prax-1, and mitochondrial creatine kinase. The expression of BAX, a pro-apoptotic, inner mitochondrial membrane protein, is also reduced in some cancer cell-lines. The metabolic imbalances and the enhanced resistance to mitochondrial apoptosis are the prominent features of cancer cells, and tumors rely heavily on glycolysis to meet their metabolic demands [16]. Mitochondrial dysfunctions have been proposed as a cause of cancer, and the citrate inhibition of glycosis has been proposed as a cancer treatment [17]. Mitochondria play crucial roles in this cancer-cell biology. Mitochrondrial dysfunction is a biomarker for the early detection of cancer, and is a therapeutic target for cancer.

Pituitary adenoma protein-mapping data clearly reveal the mitochondria-related signaling pathways that include fatty-acid elongation in mitochondria, glycolysis/gluconeogenesis, and oxidative stress that function in human pituitary adenoma cells (Figures 2 and 3). The DEP data also clearly reveal the significant signaling pathways that include mitochondrial dysfunction, oxidative phosphorylation, and oxidative-stress response in a human pituitary adenoma (Figures 6 and 7). Figure 8 shows the canonical pathway of mitochondrial dysfunctions.

Mitochondrial dysfunction could be confirmed with a mitochondrial morphological change in a human pituitary tumor. Studies found an increased number of mitochondria, and ultrastructurally abnormal mitochondria were present in pituitary oncocytomas [18]. Large mitochondria and mitochondrial swelling were present in a pituitary adenoma with hyperprolactinemia; those findings could be attributable to the prolonged intake of steroids and/or ischemia [19]. Characteristic vesicular mitochondria are present in adrenocortical-pituitary hybrid tumor cells that cause Cushing's syndrome [20]. Moreover, melatonin as an apoptotic inducer of tumor cells significantly inhibited prolactinoma cell proliferation, increased prolactinoma cell apoptosis, induced mRNA expression of Bax and cytochrome c protein expression, and, conversely, inhibited the mRNA expression of Bcl-2 and the mitochondrial membrane potential. Those data suggest that melatonin inhibits proliferation and induces apoptosis of a rat pituitary prolactin-secreting tumor via a perturbation of mitochondria physiology [21]. The ultrastructure of cells shows giant mitochondria and an increased number of mitochondria comparable to oncocytic adenomas in human pituitary adenomas that secrete growth hormone and prolactin, and electron microscopy shows many, in part densely arranged, mitochondria in human non-functioning pituitary adenomas [22]. Anti-mitochondrial staining shows intense and granular mitochondria, and electron microscopy shows swollen mitochondria in the cytoplasm with featured lamellar cristae in the spindle-cell oncocytoma of the adenohypophysis [23].

Evidence demonstrates that dysfunctional mitochondria, not oxygen insufficiency, cause cancer cells to produce inordinate amounts of lactic acid that impact on the treatment of cancer [24]. Studies demonstrate that cancer-cell mitochondria are pro-apoptotic targets for the marine antitumor drug lamellarin D [25]. Mitochondria are emerging as biomarkers for the early detection of, and for novel therapeutic targets in, cancer [17,26-29]. Therefore, mitochondrial dysfunction is not only a significant signal in a human pituitary adenoma, but also provides biomarkers for early detection, and targets for novel therapeutic agents to treat a pituitary adenoma.

### Oxidative stress

Oxidative stress is produced when the balance is disturbed between an upload of free radical/reactive oxygen/nitrogen species (ROS/RNS) from *in vivo *formation and from *in vitro *environmental carcinogens, and the capability of endogenous antioxidant defense mechanisms to remove those reactive species [30,31]. Oxidative stress can cause injury to several important cellular components such as proteins, DNA, and membrane lipids. An increased formation of ROS/RNS can promote the development of tumorigenesis, and the 'normal' rates of ROS/RNS generation might account for the increased risk of cancer development [32]. The mitochondrial oxidative respiratory chain and the oxidative phosphorylation system are the central components that produce endogenous ROS such as superoxide radicals (O2^.-^). In a pathogenesis, large amounts of nitric oxide (NO), the most important RNS component, are generated from the inducible nitric oxide synthase (iNOS) system. The superoxide radicals can rapidly react with nitric oxide to produce the more toxic peroxynitrite anion (ONOO^-^), or the highly reactive hydroxyl radical (OH^.^), to attack proteins, DNA, and membrane lipids. Many studies have indicated the presence of NOS in the human and rat pituitary [33-37], and the increased activities of NOS and its mRNA have been found in pituitary adenomas relative to controls [37,38]. NO is involved in the hypothalamic-pituitary-adrenocortical axis [39]. NO plays an important role to activate the release of luteinizing hormone-releasing hormone (LHRH) and follicle-stimulating hormone-releasing hormone (FSHRH) from the hypothalamus, and of LH and FSH from the pituitary [40-42]; to stimulate or inhibit the secretion of PRL [43]; to regulate the secretion of growth hormone (GH) in the normal human pituitary and in acromegaly [44,45]; and to modulate GH secretion in a dose-dependent manner in GH adenomatous cells from human pituitary adenomas [46].

Our nitroproteomics study [4] discovered nine tyrosine-nitrated proteins in human pituitary adenoma tissues, and that each tyrosine nitration site is located within an important protein domain to alter protein functions. However, with the formation of ROS and RNS, the *in vivo *antioxidative mechanism is also initiated against ROS/RNS [47]. The anti-oxidative system includes enzymatic [superoxide dismutase (CuZnSOD, MnSOD), catalase, glutathione peroxidase] and non-enzymatic [Vitamin C, Vitamin E, carotenoids, thiol antioxidants (glutathione, thioredoxin, and lipoic acid), flavonoids, selenium, and others] antioxidants, as well as the antioxidant interactions with various regulatory factors such as NF-κB, AP-1, and Nrf2 [47]. Copper- and zinc-containing superoxide dismutase (CuZnSOD) in most parts of cells and manganese-containing superoxide dismutase (MnSOD) in the mitochondrial matrix can effectively scavenge the superoxide radicals to generate H2O2 [48-50], which is removed by peroxiredoxins (thioredoxin-dependent peroxidase enzymes) and GPX's (glutathione peroxidases) [51,52]. The decrease or deficiency of the activities of those antioxidative enzymes could contribute to tumorigenesis [52,53]. Studies show that the content of CuZnMOD is markedly higher in normal cells than in pituitary adenoma cells [54]. Pivotal to the antioxidant response is the transcription factor Nrf2 (nuclear factor-E2-related factor-2) that is mainly located in the cytoplasm under basal conditions [55]. Under oxidative stress derived from the accumulation of ROS [56,57] and RNS [58,59], Nrf2 can quickly translocate into the nucleus and elicit an antioxidant response. Nrf2 signaling is regulated by multiple components [55,60]. At least four components in combination [Nrf2, Keap1 (Kelch-like ECH-associated protein 1), a group of small musculoaponeurotic fibrosarcoma (Maf) proteins, and a cis-acting enhancer called antioxidant response element (ARE) or electrophile responsive element (EpRE)] are essential for the antioxidant response. The Nrf2 signaling pathway not only regulates the expression of antioxidative genes, but also regulates the anti-inflammatory response, the molecular chaperone/stress-response system, and the ubiquitin/proteasome system [61]. Any decrease in the capability of this antioxidant protective system could increase the susceptibility to oxidative stress, tumor inflammation, carcinogen toxicity, and tumorigenesis.

Our pathway analysis of pituitary adenoma protein-mapping data and DEP's has clearly revealed the oxidative stress and Nrf2-mediated oxidative stress-response pathway (Figures 2, 3, 6, and 7) in pituitary adenomas. Figure 4 presents the canonical pathway of the Nrf2-mediated oxidative-stress response. Therefore, the oxidative stress-antioxidative stress-response system is not only a significant signaling pathway of pituitary adenoma formation, but also those components in this pathway could be the novel targets to develop effective therapeutic agents that could be used for human pituitary adenomas [61,62].

### Cell-cycle dysregulation

The basic biological characteristics of tumor cells are the unrestricted proliferation and growth compared to normal cells; the latter are in a state of balance between restricted proliferation and apoptosis. This proliferation process is controlled by the cell cycle. The cell cycle includes four phases (G0) G1 → S → G2 → M → G1 (G0) that will make the cell grow, replicate their genome, and divide; this cycle is regulated by a cyclically operating biochemical system that includes cyclins, cyclin-dependent kinases (CDK), and their inhibitors (CDKI) [63]. The CDKI families mainly include the INK family (INK4a/p16, INK4b/p15, INK4c/p18, and INK4d/p19) and the WAF/KIP family (WAF1/p21, KIP1/p27, and KIP2/p57). The progression through a cell cycle is mainly regulated by the fluctuations in the concentration of cyclins and CDKI that is achieved through the programmed degradation of these proteins by the proteolysis within the ubiquitin-proteasome system [64]. Cyclin D1 is expressed at the G0/G1 transition, and is involved in the regulation of progression through G1 into the S phase. Cyclin E expression occurs at the beginning of G1, maximizes at the G1/S transition, is degraded at the beginning of the S phase, and is involved in DNA replication. Cyclins D and E, in combination with CDKs/CDKI, regulate the G1 and S phases to prepare for cell division. Cyclin A accumulates in late G1, maximizes during the S phase, and is degraded in the M phase. Cylin B is necessary for the transition from G2 to mitosis. Studies have demonstrated that the ectopic expression of cyclin D and the overexpresion of Cyclins A, B, and E occur in a pituitary adenoma to regulate different phases of the cell cycle, and to accelerate the progression of the cell-cycle [63]. The overexpressed pituitary tumor-transforming gene (PTTG), as an early change in pituitary tumorigenesis, is also dependent on the cell cycle; PTTG expression is low at the G1/S border, gradually increases during the S phase, peaks at the G2/M, and is attenuated as the cells enter G1 [65]. The details on cell-cycle dysregulation in a human pituitary adenoma have been reviewed [64,66-68].

The pathway analysis of our pituitary adenoma nitroproteomic data clearly revealed the cell-cycle G2/M DNA damage checkpoint-regulation pathway in human pituitary adenomas (Figure 11). Additional file 2, **Figure S3.4 **shows the canonical pathway of the cell-cycle G2/M DNA damage checkpoint regulation. DEP data clearly demonstrate that the important cell-cycle regulator 14-3-3 protein was down-regulated (44-fold) in pituitary adenomas compared to controls (Additional file 1, **Table S2**). Moreover, our nitroproteomic data demonstrate that a nitrated proteasome could interfere with the functions of the ubiquitin-proteasome system in the regulation of the cell cycle. Thus, oxidative/nitrative stress is also involved in the cell-cycle dyregulation in human pituitary adenomas. Furthermore, those components that regulate the cell cycle could be the novel targets for the development of an effective pituitary adenoma therapy; for example, the proteasome inhibitors can induce apoptosis in growth hormone-and prolactin-secreting rat pituitary tumor cells through a blocking of the cell cycle at the G2/M transition [69].

### MAPK signaling abnormality

Mitogen-activated protein kinase (MAPK) signaling pathways play prominent roles in the between- and within-cell communications in normal cells and cancer cells [70]. Those pathways link the extracelluar signals (stimuli such as growth hormone, growth factor, mitogens, cytokines, stress, etc.) to the functional cellular processes such as growth, profliferation, migration, and apoptosis. The basic MAPK pathway is stimulus (mitogens, growth factors, cytokines, stress, etc.) → G-protein (Ras, Rac, Cdc42, Rho) → MAPKKK (Raf, Tpl2, MEKK, MLK, TAK, ASK, TAO) → MAPKK (MEK) → MAPK (ERK, JNK, P38) to generate responses (proliferation, differentiation, apoptosis, and migration). ERKs (extracellular signal-regulated kinases), JNKs (c-Jun N-terminal kinases), and p38-MAPKs are the three main subfamilies of MAPKs. ERK 1/2 are activated by MEK1/2, which are activated by Raf, Ras, and growth factors or mitogens; Raf activity, as the main effector of Ras, is suppressed by cyclic AMP-dependent kinase (PKA) in a normal cell [71]. JNKs are activated by MEK4/7, and p38-MAPKs are activated by MEK3/4/6. The upstream signal of MEK3/4/6/7 is from Rac, Rho, cdc42, cytokines, or stresses. ERKs function in the control of cell division. JNKs are critical regulators of transcription, and have the ability to promote apoptosis; however, the activation of nuclear factor kappa B (NF-κB) signaling can lead to the suppression of apoptosis. JNK and NF-κB signaling often play opposing roles in cancer. The activation of NF-κB is required to suppress JNK-activated apoptosis during tumorigenesis [72,73]. The p38-MAPKs are strongly activated by inflammatory cytokines and environmental stresses, and p38 is required for the expression of TNFα and interleukin-1 during tumor inflammatory responses. p38 can function as a tumor suppressor; a decrease of p38 activity significantly contributes to tumorigenesis [74]. Recent findings show that the cancerous mutations in MAPK pathways frequently affect Ras and B-raf. Ras/Raf mutation-activated pathways are important for cell survival and proliferation, whereas stress-activated pathways such as JNK and p38 largely seem to counteract malignant transformation. The balance and integration among those signal pathways could significantly contribute to tumorigenesis and to any response to drug therapy. The details of MAPK signaling pathways in cancer are reviewed [70,75,76]. The MAPK pathways are emerging as potential therapeutic targets for cancer [77,78], and the development of inhibitors of MAPK pathways has a growing importance in cancer therapy.

The pathway analyses of our pituitary adenoma proteomic data clearly demonstrate that MAPK signaling pathways are involved in pituitary tumorigenesis. The protein-mapping data of pituitary adenomas show that ERK (Figure 1A), NFkB and F-protein (Figure 1B), MAPK, Ras, PKC and PI3K (Figure 1C), and JNK and p38-MAPK (Figure 1E) are the key nodes in their pathway networks. The comparative proteomic data show that Ras, ERK, JNK, p38-MAPK and Akt (Figure 5A), TNF and TGFb1 (Figure 5B), and MAPK and NFkB (Figure 5C) are the key nodes in their pathway networks; and that ERK/MAPK signaling (Figure 6) is the significant canonical pathway in adenomas.

The nitroproteomic data of human pituitary adenomas show that TNF and IL1B (Figure 10A) are the key nodes in their pathway networks; and that p38-MAPK signaling (Figure 11) is the significant canonical pathway that participates in an oxidative-stress response in an adenoma. The nitroproteomic data of human pituitary controls show that TGFb1 (Figure 10B) is the key node in its pathway network. Moreover, the PKA type I beta regulatory subunit is nitrated in human pituitary adenomas (Figure 10A; Additional file 1, **Table S3**), and that tyrosine nitration occurs within the dimerization region [4]; those nitrations could interfere with dimerization and affect PKA activity to suppress Raf activity. Figure 9 shows the canonical pathway of ERK/MAPK signaling, and Figure 13 the canonical pathway of p38 MAPK signaling. Studies have demonstrated the altered Gs and adenylate cyclase activity in human GH-secreting pituitary adenomas [79], Gsα and Giα mutations in clinically nonfunctioning pituitary adenomas [80], and an H-ras mutation in a single aggressive prolactinoma or metastases from pituitary carcinomas [81]. Recent studies demonstrate that an overexpression of B-Raf mRNA and protein is a feature of nonfunctional pituitary adenomas; that overactivity highlights an overactivity of the Ras-B-Raf-MAP kinase pathway to promote pituitary tumorigenesis [82], and that the low levels of Raf kinase inhibitory protein (RKIP) in a GH-pituitary adenoma correlate with poor clinical response to somatostatin analog therapy because RKIP can bind to and inhibit Raf1 kinase to attenuate MAPK signaling [83]. The antiproliferative effect of somatostatin analogs involves the upregulation of p27 and downregulation of the MAPK pathway in human somatotrophinomas [84]. Furthermore, studies demonstrate that dopamine induces an anti-proliferative effect and cell death via the dopamine D2 receptors, by means of the p38 MAPK and ERK pathways that involve oxidative stress, in pituitary tumor cells [85]. Those data confirm that ERK-MAPK and p38-MAPK signaling pathways significantly function in human pituitary adenomas.

#### Strength and limitation

The strength of this study is (i) that, towards our long-term goals to clarify the molecular mechanism that are involved in pituitary adenoma pathogenesis and to discover tumor biomarkers, a series of human pituitary proteomic expression data (protein-mapping data, comparative proteomic data, and nitroprotemic data) were established; four important significant signal pathway networks that were derived from those proteomic expression data were discovered, including mitochondrial dysfunction, oxidative stress, cell-cycle dysregulation, and the MAPK signaling system; knowledge of those signal pathway networks will provide important clues and clear directions for our next step, an in-depth investigation of pituitary adenomas, for the discovery of tumor biomarkers, and for the development of efficacious therapeutic targets and drugs; (ii) that all protein data were confirmed with a "gold standard" tandem mass spectrometry-based amino acid sequence; and comparative proteomic data were confirmed with comparative transcriptomic data [2,3]; (iii) that signal pathway networks derived from protein-mapping data provide the baseline data; comparative proteomic data that are involved in pathway networks reveal the protein expression change in the pathway networks to clarify the role of pathway networks in the pituitary pathogenesis; and nitroproteomic data reveal the role of oxidative stress in signal pathways that are related to pituitary pathogenesis.

We realize a potential limitation of this study - a normal pituitary is an admixture of at least six pituitary cell types, whereas pituitary adenomas are generally an expanded clone of a single cell type, as described in our previous publication [86]. This factor is a common problem with any human post-surgical tissue study. Enrichment of a single cell type of pituitary cells (such as with laser-capture microdissection, LCM) in our next in-depth investigation would be an effective method to resolve that potential limitation when the LCM-sensitivity problem is overcome for pituitary protein analysis.

#### Statistical consideration and biological significance

The objective of this study is to discover significant signal pathways or pathway networks from pituitary adenoma protein-mapping data, comparative proteomic data, and nitroproteomic data. The Fisher's exact test that is contained in the IPA program was used to uncover any statistically significant pathways or networks with a significance level of 0.05. For those four protein datasets in this study: we identified 37 significant canonical pathways and 6 pathway networks derived from our protein-mapping dataset, 9 significant canonical pathways and 3 pathway networks derived from our comparative proteomic dataset, 12 significant canonical pathways and 1 pathway network derived from our qualitative nitroproteomic dataset in adenomas, and 12 significant canonical pathways and 1 pathway network derived from our qualitative nitroproteomic dataset in controls.

No multiple-test correlation and significance level of 0.01 or 0.001 was used for this study based on two reasons: (1) a multiple-test correlation and significance level of 0.01 or 0.001 are more stringent criteria. Although those two parameters can reduce the probability of false positives, they also result in the loss of any biologically meaningful information. For example, if we use the significance level of 0.001 [or -log (0.001) = 3], then there will be 7 statistically significant canonical pathways (Figure 2) and 4 significant toxicological events (Figure 3) that derived from protein-mapping data; no significant canonical pathways (Figure 6) and no significant toxicological events (Figure 7) from comparative proteomics data; 1 significant canonical pathway (Figure 11) and 1 significant toxicological event (Figure 12) from adenoma nitroproteomic data; and 10 significant canonical pathways (Figure 14) from normal pituitary nitroproteomic data. In fact, many biologically significant DEPs (Additional file 1, **Table S2**) are derived from important pathways. Also, in Figure 11, more stringent criteria simply result in a significant canonical pathway - hepatic cholestasis; however, this pathway does not have much biologically meaning for pituitary adenomas. On the other hand, the canonical pathways p38 MAPK signaling, cell-cycle G2/M DNA damage-checkpoint regulation, and protein-ubiquitination pathways (Figure 11) were recognized as statistically significant with a significance level of 0.05, which can be reasonably linked to the real pituitary adenoma biological processes (described above). (2) Any statistical result is only a reference for biological significance. A statistically significant result must be reasonably interpreted with corresponding biological processes to decide its biological significance. Some statistically significant results would not have any real biological meaning. A typical example is that hemoglobin is often identified as statistically significant between tumor and control tissues; however, it cannot be concluded as biologically meaningful for a pituitary adenoma because its statistical significance probably derived from blood contamination. The canonical pathway hepatic cholestasis described above is another example. Moreover, for some cases, there might not be any statistical significance, but those proteins still have biological significance. For example, some genes have only a small change without any significant difference at the gene level; however, that small change at the gene level could lead to an amplified change on the protein level. As a biologist, this finding is still an interesting result. Therefore, when one uses a statistically significant pathway and network, one must carefully determine whether it is biologically relevant or whether the result really just occurs only by chance.

Based on those statistical considerations, those statistically significant pathways and networks that were generated from the Fisher's exact test with a significance level of 0.05 were reasonably explained within the pituitary adenoma biological system. Therefore, four important biological systems were discovered for pituitary adenomas, including mitochondrial dysfunction, oxidative stress, cell-cycle dysregulation, and the MAPK signaling abnormality. These four biological systems provide important clues and a clear direction for our next in-depth studies of pituitary adenomas.

## Conclusions

This present study clarifies pathway networks that function in pituitary adenomas. The results demonstrate that mitochondria dysfunctions, oxidative stress, cell-cycle dysregulation, and the MAPK-signaling system are significantly associated with pituitary adenoma pathogenesis. Further experimental investigation is required to elucidate the biological consequences of those pathway networks and their relevance to the pathogenic mechanisms of pituitary adenoma. Those data could provide biomarkers, and could lead to the development of novel efficacious targets to treat pituitary adenomas.

## Methods

### Patients and tumor characterization

For protein-mapping analysis [1], the pituitary adenoma tissue (n = 1; from a black male 54-year-old) from the Memphis Regional Medical Center was used. During surgery, the tissue was removed, frozen immediately in liquid nitrogen, and stored (-80°C) until analysis.

For comparative proteomics analysis [2,3], 15 pituitary tumors and 8 normal pituitary glands were used (Additional file 1, **Table S5**). Pituitary tumors (n = 15) were obtained from patients at the Emory University Hospital during transsphenoidal surgery. All tumors were microdissected and removed with a surgical microscope, rinsed in sterile saline, snap-frozen in liquid nitrogen, and stored (-80°C) until analysis. Each tumor fragment was confirmed independently by a neuropathologist as being homogenous and unadulterated by histology and immunohistochemistry prior to proteomics analysis. Eight control pituitary glands were obtained from the Memphis regional Medical Center (n = 7) and the National Disease Research Interchange (n = 1).

For nitroproteomics analysis [4-6], the clinically nonfunctional human pituitary adenoma tissue (n = 1) [4] and the normal pituitary post-mortem tissue (n = 1) [5,6] were used. The pituitary adenoma tissue (n = 1; from a white male 39-year-old) was obtained from the University of Tennessee Baptist Hospital (Memphis, TN, USA); immunohistochemical studies showed that tumor cells were negative for the expression of FSH, LH, GH, PRL, TSH, or ACTH. During surgery, the tumor tissue was removed, frozen immediately in liquid nitrogen, and stored (-80°C) until analysis. The control pituitary tissue (n = 1; from a male 45-year-old, drowning) was obtained from the Memphis Regional Medical Center.

### Experimental datasets

The experimental datasets that were analyzed in this study derived from our published human pituitary adenoma proteomic data: (i) a protein-mapping dataset that includes 111 proteins that were identified with two-dimensional gel electrophoresis (2DGE) and MS [1] (Additional file 1, **Table S1**), (ii) a comparative proteomic dataset that includes 56 DEP's that were identified with 2D gel-based comparative proteomics [2,3] (Additional file 1, **Table S2**), (iii) a nitroproteomic dataset that includes nine nitroproteins, and three non-nitrated proteins that interacted with nitroproteins from a pituitary adenoma [4], that were identified with nitrotyrosine immunoaffinity enrichment and tandem mass spectrometry (MS/MS) (Additional file 1, **Table S3**), and eight nitroproteins from a pituitary control that were identified with 2DGE-based nitrotyrosine Western blots and MS/MS [5,6] (Additional file 1, **Table S4**).

### Ingenuity pathway analysis

The SwissProt accession number and gene name were used as the identifiers of each proteomic dataset. Each dataset was saved as an Excel file. Each proteomic dataset with identifier (.xls file) was input into the IPA analysis system http://www.ingenuity.com with the Core analysis platform. For the pituitary adenoma protein-mapping data and nitroprotein data, their Swiss-Prot accession numbers in the Excel format were input to the IPA data upload workflow. For the pituitary adenoma comparative proteomic data, the Swiss-Prot Accession numbers and the corresponding fold-change data in the Excel format were input to the IPA data upload workflow. The IPA system will automatically search the matched Gene/molecules, and will generate a two-dimensional table-style format to show which protein was mapped in the system for next-step analysis, and to show the unmapped proteins. The unmapped protein's Swiss-Prot accession number will be converted to the corresponding gene name by searching the ExPASy (Expert Protein Analysis System) proteomics server http://www.expasy.org. All Swiss-Prot accession numbers in combination with gene names were input to the IPA data upload workflow to generate the final mapped list for next-step analysis.

The dataset, including mapped IDs (protein and gene), was saved, and automatically generated five subdatasets, including the All IDs (= all input IDs), Unmapped IDs (without the matched molecules in the IPA system, unmapped IDs will not enter the next-step pathway analysis), Mapped IDs (match the corresponding molecules, and recognize the duplicate IDs), Network-eligible IDs (= Mapped IDs - Duplicated IDs), and Functions/Pathways/List-eligible IDs. For the duplicate IDs for the same protein/gene, the identifier with the highest fold-change was used in the pathway analysis; or, the first instance of the protein/gene was used in the pathway analysis in the absence of an expression value such as mapping proteomic data and nitroprotein data. Each subdataset contained ID, notes, molecules, description, location, type of biofunction, and drugs (Additional file 1, **Table S1-S4**); and the fold-change (Additional file 1, **Table S2**). The name of each molecule (gene; protein) appears in the pathway network nodes.

The Network-eligible IDs proceeded into the pathway network analysis by comparing the network-eligible molecules (genes; proteins) with the Ingenuity Pathways Analysis Knowledge Base (IPAKB). IPAKB is a curated database that contains (i) numerous scientific findings (n = ~2.2 million; February 13, 2009) that are extracted from hundreds of thousands of journal articles, textbooks, and other data sources, and (ii) many canonical pathways (n = 235; February 13, 2009) that are constructed from those scientific findings [87]. The significance (p-values) of the association between the dataset and the canonical pathway was measured by comparing the number of use-specific proteins of interest that participate in a given pathway to the total number of occurrences of these genes in all pathway annotations that are stored in the IPAKB. A Fisher's exact test was used to calculate the p-value to determine the probability that the association between the genes in the dataset and the canonical pathway is explained only by chance. The level of statistical significance was set to p < 0.05. Each Pathway analysis generated the top networks, biofunctions/Tox functions, and top canonical pathways with a statistical significance (p < 0.05). A toxicity pathway is defined as a canonical pathway that is significantly associated with toxicity lists that are functional gene groupings based on critical biological processes and key toxicological responses; and those toxicity lists describe adaptive, defensive, or reparative responses to xenobiotic insult, and could be used to understand biological responses.

## Abbreviations

ACTH: adrenocorticotropic hormone; ARE: antioxidant-response element; CDK: cyclin-dependent kinase; CDKI: cyclin-dependent kinase inhibitor; DEP's: differentially expressed proteins; 2DGE: two-dimensional gel electrophoresis; EpRE: electrophile-responsive element; ERK: extracellular signal-regulated kinase; FSH: follicle-stimulating hormone, or follitropin; FSHRH: follicle-stimulating hormone-releasing hormone; GH: growth hormone; IPA: Ingenuity Pathways Analysis; IPAKB: Ingenuity Pathways Analysis Knowledge Base; JNK: c-Jun N-terminal kinase; Keap1: Kelch-like ECH-associated protein 1; LH: luteinizing hormone, or lutropin; LHRH: luteinizing hormone-releasing hormone; Maf: musculoaponeurotic fibrosarcoma; MAPK: mitogen-activated protein kinase; MAPKK: MAPK kinase; MAPKKK: MAPKK kinase; MS: mass spectrometry; MS/MS: tandem mass spectrometry; NF-κB: nuclear factor kappa B; NO: nitric oxide; Nrf2: nuclear factor E2-related factor-2; PKA: cyclic AMP-dependent kinase; PRL: prolactin; PTTG: pituitary tumor-transforming gene; RKIP: raf kinase inhibitory protein; RNS: reactive nitrogen species; ROS: reactive oxygen species; SOD: superoxide dismutase; TSH: thyrotropin-stimulating hormone, or thyrotropin.

## Competing interests

The authors declare that they have no competing interests.

## Authors' contributions

XZ conceived the study design, performed experiments, analyzed the data, and wrote the manuscript. DMD oversaw the experimental results, and reviewed and modified the manuscript. All the authors have read and approved the final manuscript.

## Pre-publication history

The pre-publication history for this paper can be accessed here:

http://www.biomedcentral.com/1755-8794/3/13/prepub

## Supplementary Material

Additional file 1 **Supplementary Tables**. This file contains supplementary tables S1-S5. **Supplementary Table S1 **shows the protein-mapping data identified from a pituitary adenoma tissue with two-dimensional gel electrophoresis (2DGE) and mass spectrometry. **Supplementary Table S2 **shows comparative proteomic data identified from human pituitary adenoma tissues with 2DGE-based comparative proteomics. **Supplementary Table S3 **shows nitroproteins, and proteins that interact with nitroproteins, identified from a human pituitary adenoma tissue with nitrotyrosine affinity enrichment and tandem mass spectrometry. **Supplementary Table S4 **shows nitroproteins identified from human pituitary post-mortem control tissue with 2DGE-based nitrotyrosine Western Blot and tandem mass spectrometry. **Supplementary Table S5 **shows clinical and pathological characteristics of pituitary adenomas and controls used in comparative proteomics study. Click here for file

Additional file 2 **Supplementary Figures**. This file contains supplementary figures S1-S4. **Supplementary Figure S1 **shows significant canonical pathways that are involved in human pituitary adenoma mapping proteomic data. **Supplementary Figure S2 **shows significant canonical pathways that are involved in human pituitary adenoma comparative proteomic data. **Supplementary Figure S3 **shows significant canonical pathways that are involved in human pituitary adenoma nitroproteomics data. **Supplementary Figure S4 **shows significant canonical pathways that are involved in human pituitary control nitroproteomics data. Click here for file
