# Supplementary material for: Signaling pathway networks mined from human pituitary adenoma proteomics data
Source: BMC Med Genomics. 2010 Apr 28;3:13. doi: 10.1186/1755-8794-3-13 (PMC2884164; doi:10.1186/1755-8794-3-13)
Supplement: Additional file 1 — Supplementary Tables. This file contains supplementary tables S1-S5. Supplementary Table S1 shows the protein-mapping data identified from a pituitary adenoma tissue with two-dimensional gel electrophoresis (2DGE) and mass spectrometry. Supplementary Table S2 shows comparative proteomic data identified from human pituitary adenoma tissues with 2DGE-based comparative proteomics. Supplementary Table S3 shows nitroproteins, and proteins that interact with nitroproteins, identified from a human pituitary adenoma tissue with nitrotyrosine affinity enrichment and tandem mass spectrometry. Supplementary Table S4 shows nitroproteins identified from human pituitary post-mortem control tissue with 2DGE-based nitrotyrosine Western Blot and tandem mass spectrometry. Supplementary Table S5 shows clinical and pathological characteristics of pituitary adenomas and controls used in comparative proteomics study. [file 1755-8794-3-13-S1.PDF]

Additional File 1
Supplementary Tables

Supplementary Table S1

Proteins identified from a pituitary adenoma tissue with two-dimensional gel electrophoresis and mass spectrometry

ID	Notes	Molecules	Description	Location	Function	Drugs
P04217		A1BG	alpha-1-B glycoprotein	Extracellular Space	other	
P32391		ACTR3	ARP3 actin-related protein 3 homolog (yeast)	Plasma Membrane	other	
P30566	D	ADSL	adenylosuccinate lyase	Cytoplasm	enzyme	
P30566	D	ADSL	adenylosuccinate lyase	Cytoplasm	enzyme	
P15121		AKR1B1	aldo-keto reductase family 1, member B1 (aldose reductase)	Cytoplasm	enzyme	sorbinil
O43488		AKR7A2	aldo-keto reductase family 7, member A2 (aflatoxin aldehyde reductase)	Cytoplasm	enzyme	
P02768	D	ALB	albumin	Extracellular Space	transporter	
P02768	D	ALB	albumin	Extracellular Space	transporter	
P02768	D	ALB	albumin	Extracellular Space	transporter	
P02768	D	ALB	albumin	Extracellular Space	transporter	
P04083		ANXA1	annexin A1	Plasma Membrane	other	
P07355		ANXA2	annexin A2	Plasma Membrane	other	
P09525		ANXA4	annexin A4	Plasma Membrane	other	
P08758	D	ANXA5	annexin A5	Plasma Membrane	other	
P08758	D	ANXA5	annexin A5	Plasma Membrane	other	
P02743		APCS	amyloid P component, serum	Extracellular Space	other	
P02647	D	APOA1	apolipoprotein A-I	Extracellular Space	transporter	
P02647	D	APOA1	apolipoprotein A-I	Extracellular Space	transporter	
P06576		ATP5B	ATP synthase, H+ transporting, mitochondrial F1 complex, beta polypeptide	Cytoplasm	transporter	
O75947		ATP5H (includes EG:10476)	ATP synthase, H+ transporting, mitochondrial F0 complex, subunit d	Cytoplasm	transporter	

P30043	BLVRB	biliverdin reductase B (flavin reductase (NADPH))	Cytoplasm	enzyme	
P01024	C3	complement component 3	Extracellular Space	peptidase	
P00915	CA1 (includes EG:759)	carbonic anhydrase I	Cytoplasm	enzyme	methazolamide, hydrochlorothiazide, acetazolamide, trichloromethiazide, dorzolamide, chlorothiazide, dorzolamide/timolol, brinzolamide, chlorthalidone, benzthiazide, sulfacetamide, topiramate
P27797	CALR	calreticulin	Cytoplasm	transcription regulator	
P04632	CAPNS1	calpain, small subunit 1	Cytoplasm	peptidase	
P04040	CAT	catalase	Cytoplasm	enzyme	
P35520	CBS	cystathionine-beta-synthase	Cytoplasm	enzyme	
P51946	CCNH	cyclin H	Nucleus	transcription regulator	
P49368	CCT3	chaperonin containing TCP1, subunit 3 (gamma)	Cytoplasm	other	
P29373	CRABP2	cellular retinoic acid binding protein 2	Cytoplasm	transporter	
P32321	DCTD	dCMP deaminase	Unknown	enzyme	
O00559	EBAG9	estrogen receptor binding site associated, antigen, 9	Cytoplasm	other	
P30084	ECHS1	enoyl Coenzyme A hydratase, short chain, 1, mitochondrial	Cytoplasm	enzyme	
P10126	EEF1A1	eukaryotic translation elongation factor 1 alpha 1	Cytoplasm	translation regulator	
P26641	EEF1G	eukaryotic translation elongation factor 1 gamma	Cytoplasm	translation regulator	
P06733	ENO1	enolase 1, (alpha)	Cytoplasm	transcription regulator	
P09104	ENO2	enolase 2 (gamma, neuronal)	Cytoplasm	enzyme	
P30040	ERP29	endoplasmic reticulum protein 29	Cytoplasm	transporter	

P00742		F10	coagulation factor X	Extracellular Space	peptidase	antihemophilic factor, dalteparin, heparin, coagulation Factor VIIa, enoxaparin, coagulation factor IX, rivaroxaban, deligoparin, idraparinux, tifacogin, RPR 120844, RPR 208566, DPC 423, fondaparinux
Q9NRD0		FBXO8	F-box protein 8	Unknown	other	
P02679	D	FGG	fibrinogen gamma chain	Extracellular Space	other	thrombin
P02679	D	FGG	fibrinogen gamma chain	Extracellular Space	other	thrombin
P02679	D	FGG	fibrinogen gamma chain	Extracellular Space	other	thrombin
P02679	D	FGG	fibrinogen gamma chain	Extracellular Space	other	thrombin
P02679	D	FGG	fibrinogen gamma chain	Extracellular Space	other	thrombin
P02679	D	FGG	fibrinogen gamma chain	Extracellular Space	other	thrombin
P02679	D	FGG	fibrinogen gamma chain	Extracellular Space	other	thrombin
P02679	D	FGG	fibrinogen gamma chain	Extracellular Space	other	thrombin
P02679	D	FGG	fibrinogen gamma chain	Extracellular Space	other	thrombin
P02679	D	FGG	fibrinogen gamma chain	Extracellular Space	other	thrombin
P02679	D	FGG	fibrinogen gamma chain	Extracellular Space	other	thrombin
P02679	D	FGG	fibrinogen gamma chain	Extracellular Space	other	thrombin
P02679	D	FGG	fibrinogen gamma chain	Extracellular Space	other	thrombin
P02679	D	FGG	fibrinogen gamma chain	Extracellular Space	other	thrombin
P02679	D	FGG	fibrinogen gamma chain	Extracellular Space	other	thrombin
P02679	D	FGG	fibrinogen gamma chain	Extracellular Space	other	thrombin
P02679	D	FGG	fibrinogen gamma chain	Extracellular Space	other	thrombin
P02679	D	FGG	fibrinogen gamma chain	Extracellular Space	other	thrombin
Q14192		FHL2	four and a half LIM domains 2	Nucleus	other	
Q13451		FKBP5	FK506 binding protein 5	Nucleus	enzyme	
P02794		FTH1	ferritin, heavy polypeptide 1	Cytoplasm	enzyme	
P02792		FTL	ferritin, light polypeptide	Cytoplasm	other	
P06241		FYN	FYN oncogene related to SRC, FGR, YES	Plasma Membrane	kinase	dasatinib
P50395		GDI2	GDP dissociation inhibitor 2	Cytoplasm	other	
P01241		GH1	growth hormone 1	Extracellular Space	cytokine	

P09471	D	GNAO1	guanine nucleotide binding protein (G protein), alpha activating activity polypeptide O	Plasma Membrane	enzyme
P29777	D	GNAO1	guanine nucleotide binding protein (G protein), alpha activating activity polypeptide O	Plasma Membrane	enzyme
P11016		GNB2	guanine nucleotide binding protein (G protein), beta polypeptide 2	Plasma Membrane	enzyme
P16520		GNB3	guanine nucleotide binding protein (G protein), beta polypeptide 3	Plasma Membrane	enzyme
P06396	D	GSN	gelsolin (amyloidosis, Finnish type)	Extracellular Space	other
P06396	D	GSN	gelsolin (amyloidosis, Finnish type)	Extracellular Space	other
P28161		GSTM2	glutathione S-transferase M2 (muscle)	Cytoplasm	enzyme
P78417	D	GSTO1	glutathione S-transferase omega 1	Cytoplasm	enzyme
P78417	D	GSTO1	glutathione S-transferase omega 1	Cytoplasm	enzyme
P09211		GSTP1	glutathione S-transferase pi 1	Cytoplasm	enzyme
P55084		HADHB	hydroxyacyl-Coenzyme A dehydrogenase/3 ketoacyl-Coenzyme A thiolase/enoyl-Coenzyme A hydratase (trifunctional protein), beta subunit	Cytoplasm	enzyme
P30443	D	HLA-A	major histocompatibility complex, class I, A	Plasma Membrane	transmembrane receptor
P30455	D	HLA-A	major histocompatibility complex, class I, A	Plasma Membrane	transmembrane receptor
P07910		HNRNPC	heterogeneous nuclear ribonucleoprotein C (C1/C2)	Nucleus	other
P02790	D	HPX	hemopexin	Extracellular Space	transporter
P02790	D	HPX	hemopexin	Extracellular Space	transporter
Q99714		HSD17B10	hydroxysteroid (17-beta) dehydrogenase 10	Cytoplasm	enzyme
Q00613	D	HSF1	heat shock transcription factor 1	Nucleus	transcription regulator
Q00613	D	HSF1	heat shock transcription factor 1	Nucleus	transcription regulator
P11021		HSPA5	heat shock 70kDa protein 5 (glucose-regulated protein, 78kDa)	Cytoplasm	other
P11142	D	HSPA8	heat shock 70kDa protein 8	Cytoplasm	enzyme
P11142	D	HSPA8	heat shock 70kDa protein 8	Cytoplasm	enzyme
P04792		HSPB1	heat shock 27kDa protein 1	Cytoplasm	other

O75874	D	IDH1	isocitrate dehydrogenase 1 (NADP+), soluble	Cytoplasm	enzyme
O75874	D	IDH1	isocitrate dehydrogenase 1 (NADP+), soluble	Cytoplasm	enzyme
P50213		IDH3A	isocitrate dehydrogenase 3 (NAD+) alpha	Cytoplasm	enzyme
Q9P0V2		IMMT	inner membrane protein, mitochondrial (mitofilin)	Cytoplasm	other
Q9UBX7		KLK11	kallikrein-related peptidase 11	Extracellular Space	peptidase
P08727		KRT19	keratin 19	Cytoplasm	other
P35527		KRT9	keratin 9 (epidermolytic palmoplantar keratoderma)	Cytoplasm	other
P07195		LDHB	lactate dehydrogenase B	Cytoplasm	enzyme
O75198		LOC390688	CDC37-like	Unknown	other
P41279	D	MAP3K8	mitogen-activated protein kinase kinase kinase 8	Cytoplasm	kinase
P41279	D	MAP3K8	mitogen-activated protein kinase kinase kinase 8	Cytoplasm	kinase
P40926		MDH2	malate dehydrogenase 2, NAD (mitochondrial)	Cytoplasm	enzyme
P16475	D	MYL6	myosin, light chain 6, alkali, smooth muscle and non-muscle	Cytoplasm	other
P24572	D	MYL6	myosin, light chain 6, alkali, smooth muscle and non-muscle	Cytoplasm	other
P24844		MYL9 (includes EG:10398)	myosin, light chain 9, regulatory	Cytoplasm	other
P54920		NAPA	N-ethylmaleimide-sensitive factor attachment protein, alpha	Cytoplasm	transporter
O75489		NDUFS3	NADH dehydrogenase (ubiquinone) Fe-S protein 3, 30kDa (NADH-coenzyme Q reductase)	Cytoplasm	enzyme
P22392		NME2	non-metastatic cells 2, protein (NM23B) expressed in	Nucleus	kinase
O75665		OFD1	oral-facial-digital syndrome 1	Plasma Membrane	other
O14753	D	OVOL1	ovo-like 1(Drosophila)	Nucleus	transcription regulator
O14753	D	OVOL1	ovo-like 1(Drosophila)	Nucleus	transcription regulator

P07237		P4HB	procollagen-proline, 2-oxoglutarate 4-dioxygenase (proline 4-hydroxylase), beta polypeptide	Cytoplasm	enzyme	
Q29459		PAFAH1B2	platelet-activating factor acetylhydrolase, isoform Ib, beta subunit 30kDa	Cytoplasm	enzyme	
Q15365		PCBP1 (includes EG:5093)	poly(rC) binding protein 1	Nucleus	translation regulator	
P30101		PDIA3	protein disulfide isomerase family A, member 3	Cytoplasm	peptidase	
P18669		PGAM1	phosphoglycerate mutase 1 (brain)	Cytoplasm	phosphatase	
O95336		PGLS	6-phosphogluconolactonase	Cytoplasm	enzyme	
P35232		PHB (includes EG:5245)	prohibitin	Nucleus	transcription regulator	
Q00169		PITPNA	phosphatidylinositol transfer protein, alpha	Cytoplasm	transporter	
Q9NTG1		PKDREJ	polycystic kidney disease (polycystin) and REJ homolog (sperm receptor for egg jelly homolog, sea urchin)	Unknown	ion channel	
P05092	D	PPIA	peptidylprolyl isomerase A (cyclophilin A)	Cytoplasm	enzyme	N-methyl-4-Ile-cyclosporin
P05092	D	PPIA	peptidylprolyl isomerase A (cyclophilin A)	Cytoplasm	enzyme	N-methyl-4-Ile-cyclosporin
P32119	D	PRDX2	peroxiredoxin 2	Cytoplasm	enzyme	
P32119	D	PRDX2	peroxiredoxin 2	Cytoplasm	enzyme	
P30041		PRDX6	peroxiredoxin 6	Cytoplasm	enzyme	
O43422		PRKRIR	protein-kinase, interferon-inducible double stranded RNA dependent inhibitor, repressor of (P58 repressor)	Nucleus	other	
Q03527		PSMC1	proteasome (prosome, macropain) 26S subunit, ATPase, 1	Nucleus	peptidase	
Q06323		PSME1	proteasome (prosome, macropain) activator subunit 1 (PA28 alpha)	Cytoplasm	other	
Q9UL46		PSME2	proteasome (prosome, macropain) activator subunit 2 (PA28 beta)	Cytoplasm	peptidase	

P09455		RBP1	retinol binding protein 1, cellular	Extracellular Space	transporter	
P05121		SERPINE1	serpin peptidase inhibitor, clade E (nexin, plasminogen activator inhibitor type 1), member 1	Extracellular Space	other	drotrecogin alfa
Q13435		SF3B2	splicing factor 3b, subunit 2, 145kDa	Nucleus	other	
P00441		SOD1	superoxide dismutase 1, soluble (amyotrophic lateral sclerosis 1 (adult))	Cytoplasm	enzyme	
P30626		SRI	sorcin	Cytoplasm	transporter	
Q9P2R7	D	SUCLA2	succinate-CoA ligase, ADP-forming, beta subunit	Cytoplasm	enzyme	
Q9P2R7	D	SUCLA2	succinate-CoA ligase, ADP-forming, beta subunit	Cytoplasm	enzyme	
P02787		TF	transferrin	Extracellular Space	transporter	
P00938		TPI1	triosephosphate isomerase 1	Cytoplasm	enzyme	
P06753	D	TPM3	tropomyosin 3	Cytoplasm	other	
P06753	D	TPM3	tropomyosin 3	Cytoplasm	other	
P07226		TPM4	tropomyosin 4	Cytoplasm	other	
P09936		UCHL1	ubiquitin carboxyl-terminal esterase L1 (ubiquitin thiolesterase)	Cytoplasm	peptidase	
Q99536		VAT1	vesicle amine transport protein 1 homolog (T. californica)	Plasma Membrane	transporter	
P45880		VDAC2	voltage-dependent anion channel 2	Cytoplasm	ion channel	
P12956		XRCC6	X-ray repair complementing defective repair in Chinese hamster cells 6 (Ku autoantigen, 70kDa)	Nucleus	enzyme	
P31946	D	YWHAB	tyrosine 3-monooxygenase/tryptophan 5-monooxygenase activation protein, beta polypeptide	Cytoplasm	other	
P31946	D	YWHAB	tyrosine 3-monooxygenase/tryptophan 5-monooxygenase activation protein, beta polypeptide	Cytoplasm	other	
P42655		YWHAE	tyrosine 3-monooxygenase/tryptophan 5-monooxygenase activation protein, epsilon polypeptide	Cytoplasm	other	
P27348		YWHAQ (includes EG:10971)	tyrosine 3-monooxygenase/tryptophan 5-monooxygenase activation protein, theta polypeptide	Cytoplasm	other	

P08107	U	Heat shock 70 kDa protein 1
gi2460318	U	RNA-binding protein regulatory subunit
gi14249382	U	Hypothetical protein MGC15429
P02023	U	Hemoglobin beta chain
gi12805429	U	Similar to DJ-1 protein
P38607	U	Vacuolar ATP synthase catalytic subunit A, osteoclast isoform
P10990	U	Actin 15A

Note: D = duplicate. U = unmapped. All IDs = 154. Mapped IDs = 147. Unmapped IDs = 7. Network eligible IDs = 106.

Supplementary Table S2

Differentially Expressed Proteins Identified From Human Pituitary Adenoma tissues with 2DGE-based Comparative Proteomics

ID	Notes	Molecules	Fold-Change	Description	Location	Function	Drugs
P49753		ACOT2	5.8	acyl-CoA thioesterase 2	Cytoplasm	enzyme	
P15121		AKR1B1	14.6	aldo-keto reductase family 1, member B1 (aldose reductase)	Cytoplasm	enzyme	orbinil
P02647		APOA1	-3.2	apolipoprotein A-I	Extracellular Space	transporter	
P06576		ATP5B	5.0	ATP synthase, H+ transporting, mitochondrial F1 complex, beta polypeptide	Cytoplasm	transporter	
P47756	D	CAPZB	6.5	capping protein (actin filament) muscle Z-line, beta	Cytoplasm	other	
CAPZB	D	CAPZB	6.5	capping protein (actin filament) muscle Z-line, beta	Cytoplasm	other	
P13987		CD59	-9.5	CD59 molecule, complement regulatory protein	Plasma Membrane	other	
P12110		COL6A2	-14.7	collagen, type VI, alpha 2	Extracellular Space	other	collagenase
P14854		COX6B1	9.3	cytochrome c oxidase subunit Vib polypeptide 1 (ubiquitous)	Cytoplasm	enzyme	
Q14894		CRYM	-35.3	crystallin, mu	Cytoplasm	enzyme	
CSN1S1		CSN1S1 (includes EG:1446)	-17.8	casein alpha s1	Extracellular Space	other	
P07108		DBI	4.3	diazepam binding inhibitor (GABA receptor modulator, acyl-Coenzyme A binding protein)	Cytoplasm	other	
Q9UHL4		DPP7	-9.3	dipeptidyl-peptidase 7	Cytoplasm	peptidase	
Q14259		ERH	5.0	enhancer of rudimentary homolog (Drosophila)	Nucleus	other	
P30040		ERP29	4.6	endoplasmic reticulum protein 29	Cytoplasm	transporter	

P00742		F10	-83.0	coagulation factor X	Extracellular Space	peptidase	antihemophilic factor, dalteparin, heparin, coagulation Factor VIIa, enoxaparin, coagulation factor IX, rivaroxaban, deligoparin, idraparinux, tifacogin, RPR 120844, RPR 208566, DPC 423, fondaparinux
P06241		FYN	3.9	FYN oncogene related to SRC, FGR, YES	Plasma Membrane	kinase	dasatinib
P31150		GDI1	9.4	GDP dissociation inhibitor 1	Cytoplasm	other	
P01241	D	GH1	-180.6	growth hormone 1	Extracellular Space	cytokine	
P01241	D	GH1	-13.3	growth hormone 1	Extracellular Space	cytokine	
P01241	D	GH1	-13.3	growth hormone 1	Extracellular Space	cytokine	
P01241	D	GH1	-20.2	growth hormone 1	Extracellular Space	cytokine	
P01241	D	GH1	-14.1	growth hormone 1	Extracellular Space	cytokine	
P01241	D	GH1	-128.3	growth hormone 1	Extracellular Space	cytokine	
P01241	D	GH1	-14.8	growth hormone 1	Extracellular Space	cytokine	
P01241	D	GH1	-48.1	growth hormone 1	Extracellular Space	cytokine	
P01241	D	GH1	-17.3	growth hormone 1	Extracellular Space	cytokine	
P01241	D	GH1	-16.9	growth hormone 1	Extracellular Space	cytokine	
P01241	D	GH1	-88.8	growth hormone 1	Extracellular Space	cytokine	
P01241	D	GH1	-27.8	growth hormone 1	Extracellular Space	cytokine	
P01241	D	GH1	-51.2	growth hormone 1	Extracellular Space	cytokine	
P01241	D	GH1	-32.1	growth hormone 1	Extracellular Space	cytokine	
P01241	D	GH1	-478.6	growth hormone 1	Extracellular Space	cytokine	

P01241	D	GH1	-13.4	growth hormone 1	Extracellular Space	cytokine	
P01241	D	GH1	-161.0	growth hormone 1	Extracellular Space	cytokine	
P01241	D	GH1	-37.0	growth hormone 1	Extracellular Space	cytokine	
P01242		GH2	-14.1	growth hormone 2	Extracellular Space	other	
Q04760	D	GLO1	10.2	glyoxalase I	Cytoplasm	enzyme	
Q04760	D	GLO1	8.3	glyoxalase I	Cytoplasm	enzyme	
P09471	D	GNAO1	9.5	guanine nucleotide binding protein (G protein), alpha activating activity polypeptide O	Plasma Membrane	enzyme	
P29777	D	GNAO1	9.5	guanine nucleotide binding protein (G protein), alpha activating activity polypeptide O	Plasma Membrane	enzyme	
P36969		GPX4	-26.1	glutathione peroxidase 4 (phospholipid hydroperoxidase)	Cytoplasm	enzyme	
P28161		GSTM2	4.2	glutathione S-transferase M2 (muscle)	Cytoplasm	enzyme	
HBB	D	HBB (includes EG:3043)	-3.1	hemoglobin, beta	Cytoplasm	transporter	
HBB	D	HBB (includes EG:3043)	-99.4	hemoglobin, beta	Cytoplasm	transporter	
P02081		HBD	-3.1	hemoglobin, delta	Cytoplasm	transporter	
P14625		HSP90B1	-11.0	heat shock protein 90kDa beta (Grp94), member 1	Cytoplasm	other	17-dimethylaminoethylamino-17-demethoxygeldanamycin, IPI-504
P04792		HSPB1	-5.1	heat shock 27kDa protein 1	Cytoplasm	other	
Q9UJY1		HSPB8	-3.7	heat shock 22kDa protein 8	Cytoplasm	kinase	
O75874		IDH1	8.7	isocitrate dehydrogenase 1 (NADP+), soluble	Cytoplasm	enzyme	
P24592		IGFBP6	-20.9	insulin-like growth factor binding protein 6	Extracellular Space	other	
IGLC1		IGLC1	-32.1	immunoglobulin lambda constant 1 (Mcg marker)	Cytoplasm	other	

IGLC3		IGLC3	-32.1	immunoglobulin lambda constant 3 (Kern-Oz+ marker)	Unknown	other
P08779		KRT16	-28.8	keratin 16 (focal non-epidermolytic palmoplantar keratoderma)	Cytoplasm	other
Q86U44		METTL3	-8.3	methyltransferase like 3	Nucleus	enzyme
Q99542		MMP19	3.1	matrix metallopeptidase 19	Extracellular Space	peptidase
P12524		MYCL1	-17.8	v-myc myelocytomatosis viral oncogene homolog 1, lung carcinoma derived (avian)	Nucleus	transcription regulator
O00217		NDUFS8	5.2	NADH dehydrogenase (ubiquinone) Fe-S protein 8, 23kDa (NADH-coenzyme Q reductase)	Cytoplasm	enzyme
P20774		OGN	-38.0	osteoglycin	Extracellular Space	growth factor
Q08752		PPID	5.1	peptidylprolyl isomerase D (cyclophilin D)	Cytoplasm	enzyme
Q00007		PPP2R2A	-8.2	protein phosphatase 2 (formerly 2A), regulatory subunit B, alpha isoform	Cytoplasm	phosphatase
P01236	D	PRL	-99.9	prolactin	Extracellular Space	cytokine
P01236	D	PRL	-9.7	prolactin	Extracellular Space	cytokine
P01236	D	PRL	-26.2	prolactin	Extracellular Space	cytokine
P01236	D	PRL	-20.1	prolactin	Extracellular Space	cytokine
P01236	D	PRL	-36.7	prolactin	Extracellular Space	cytokine
P01236	D	PRL	-33.6	prolactin	Extracellular Space	cytokine
O76038		SCGN	-6.6	secretagogin, EF-hand calcium binding protein	Cytoplasm	other
Q9UI15		TAGLN3	5.6	transgelin 3	Unknown	other
P21980		TGM2	-17.1	transglutaminase 2 (C polypeptide, protein-glutamine-gamma-glutamyltransferase)	Cytoplasm	enzyme
Q8IWU9		TPH2	10.6	tryptophan hydroxylase 2	Unknown	enzyme
O14530		TXNDC9	-11.4	thioredoxin domain containing 9	Unknown	other
P08670		VIM	5.5	vimentin	Cytoplasm	other
P27348		YWHAQ (includes EG:10971)	-43.5	tyrosine 3-monooxygenase/tryptophan 5-monooxygenase activation protein, theta polypeptide (14-3-3 protein tau)	Cytoplasm	other
Q14584		ZNF266	7.3	zinc finger protein 266	Nucleus	other

IL15-S21AA	U	P40933-2	-2.7	Splice isoform IL15-S21AA of interleukin-15
P01620	U		-32.1	Ig kappa chain V-III region SIE
IGLC2	U	P01842	-32.1	Ig lambda chain C regions
P01621	U		-32.1	Ig Kappa chain V-III region NG9
P04433	U		-32.1	Ig kappa chain V-III region VG
HBB2	U	P18988	-3.1	Hemoglobin beta-2 chain (PANLE)
gi 1066765	U		-176.7	Hemoglobin beta unit variant
HBB2	U	P18988	-99.4	Hemoglobin beta-2 chain (PANLE)
P01935	U		-99.4	Hemoglobin alpha-3 chain (PANTR)
P01968	U		-20.2	Hemoglobin alpha-2 chain (BOSMU)
HBB2	U	P18988	-20.2	Hemoglobin beta-2 chain (PANLE)

Note: D = duplicate. U = unmapped. All IDs = 86. Mapped IDs = 75. Unmapped IDs = 11. Network eligible IDs = 47.

Supplementary Table S3

Nitroproteins, and Proteins That Interact With Nitroproteins, Identified From Human Pituitary Adenoma Tissue With Nitrotyrosine Affinity Enrichment and Tandem Mass Spectrometry

ID	Notes	Molecules	Description	Location	Function	Drugs
Q13017		ARHGAP5	Rho GTPase activating protein 5	Cytoplasm	enzyme	
P59901	nY; NIE	LILRA4	leukocyte immunoglobulin-like receptor, subfamily A (with TM domain), member 4	Plasma Membrane	other	
O94892	nY; NIE	ZNF432	zinc finger protein 432	Nucleus	other	
P31321	nY	PRKAR1B	protein kinase, cAMP-dependent, regulatory, type I, beta	Cytoplasm	kinase	
O95470	nY	SGPL1	sphingosine-1-phosphate lyase 1	Cytoplasm	enzyme	
Q15027	nY	CENTB1	centaurin, beta 1	Nucleus	other	
P25787	nY	PSMA2	proteasome (prosome, macropain) subunit, alpha type, 2	Cytoplasm	peptidase	
Q9UHA7	nY	IL1F6	interleukin 1 family, member 6 (epsilon)	Extracellular Space	cytokine	
Q8IUC4	nY	RHPN2	rhophilin, Rho GTPase binding protein 2	Cytoplasm	other	
O43187	Y	IRAK2	interleukin-1 receptor-associated kinase 2	Plasma Membrane	kinase	
Q9C0E4	Y	GRIP2	glutamate receptor interacting protein 2	Plasma Membrane	other	
P62988	Y	UBB	ubiquitin B	Cytoplasm	other	

Note: nY = nitrotyrosine. Y = tyrosine. All IDs = 12. Mapped IDs = 12. Unmapped IDs = 0. Network eligible IDs = 10. NIE = Network illegibile.

Supplementary Table S4

Nitroproteins Identified From Human Pituitary Post-mortem Control Tissue With 2DGE-based Nitrotyrosine Western Blot and Tandem Mass Spectrometry

ID	Notes	Molecules	Description	Location	Function	Drugs
P03996	nY	ACTA2 (includes EG:59)	actin, alpha 2, smooth muscle, aorta	Cytoplasm	other	
P04270	nY	ACTC1	actin, alpha, cardiac muscle 1	Cytoplasm	other	
P12718	nY	ACTG2 (includes EG:72)	actin, gamma 2, smooth muscle, enteric	Cytoplasm	other	
P24071	nY	FCAR	Fc fragment of IgA, receptor for	Plasma Membrane	other	
Q8IWL3	nY	HSCB	HscB iron-sulfur cluster co-chaperone homolog	Cytoplasm	other	
Q6TCH7	nY	PAQR3	progestin and adipoQ receptor family member III	Cytoplasm	other	
Q13237	nY	PRKG2	protein kinase, cGMP-dependent, type II	Cytoplasm	kinase	
P25787	nY	PSMA2	proteasome (prosome, macropain) subunit, alpha type, 2	Cytoplasm	peptidase	
O60641	nY	SNAP91	synaptosomal-associated protein, 91kDa homolog	Plasma Membrane	other	
P52823	nY	STC1	stanniocalcin 1	Extracellular Space	kinase	

Note: nY = nitrotyrosine. All IDs = 10. Mapped IDs = 10. Unmapped IDs = 0. Network eligible IDs = 9.

Supplementary Table S5

Clinical and Pathological Characteristics of Pituitary Adenomas and Controls Used in Comparative Proteomics Study

Patient	Sex, Age	Clinical features/Tumor size	Immunohistochemistry	Reference
T164	M, 35	Nonfunctional, visual loss, 3 x 3.5 x 4 cm. Particial hypopituitarism	Neg.	[2]
T219	M, 68	Nonfunctional, 1.9 x 2.3 x 2.2 cm, invasion of the right cavernous sinus	Neg.	[2]
T165	M, 56	Nonfunctional, 3.2 cm in diameter craniocaudal	LH 2+	[2]
T208	F, 47	Nonfunctional, 2 x 2 x 2 cm	LH 1-2+	[2]
T237	F, 40	Nonfunctional, right cavernous sinus extension	LH 2+	[2]
T57	F, 59	Nonfunctional, 2 x 3 cm	FSH 1+	[2]
T77	M, 67	Nonfunctional, 2 x 2.2 x 2.4 cm, questionable cavernous sinus	FSH 2+	[2]
T89	M, 62	Nonfunctional, 2 x 2.3 x 2.3 cm	FSH 2+	[2]
T65	F, 54	Nonfunctional, 4 x 4 x 4 cm, cavernous sinus invasion	FSH 1+, LH 2+	[2]
T138	M, 60	Nonfunctional, 2.9 x 3.1 x 3.5 cm	FSH 2+, LH 2+	[2]
T185	M, 66	Nonfunctional, 2.8 x 2 x 2.4 cm, bilateral cavernous sinus invasion	FSH 2-3+, LH 2-3+	[2]
T87	M, 48	Prolactinoma with calcification	PRL+	[3]
T131	F, 52	Prolactinoma, serum 359 ng/ml, 2.5 x 3.5 x 2.8 cm	PRL 3+	[3]
T192	M, 41	Prolactinoma, serum PRL 1176 ng/ml, 3 x 2.5 x 2.0 cm	PRL 3+	[3]
T273	M, 36	Prolactinoma, serum PRL 1918 ng/ml, 2.0 x 2.1 x 2.5 cm	PRL 3+	[3]

C2	M, 27	Normal	ND	[2, 3]
C3	F, 40	Normal	ND	[2, 3]
C4	M, 45	Normal	ND	[2, 3]
C5	M, 36	Normal	ND	[2, 3]
C7	F, 34	Normal	ND	[2, 3]
C8	F	Normal	ND	[2, 3]
C9	M, 55	Normal	ND	[2, 3]
C10	F, 47	Normal	ND	[2, 3]

Adenomas were graded blindly by a neuropathologist from 0 to 4 for intensity of immunohistochemistry staining for each peptide hormone. Neg. = immunohistochemical stains for ACTH, LH, FSH, PRL, GH, and TSH were negative. LH+ = Nonfunctional (NF) adenoma that expressed leuteinizing hormone, or lutropin; FSH+ = NF that expressed follicle-stimulating hormone, or follitropin; FSH+, LH+ = NF that expressed both follicule-stimulating hormone and leuteinizing hormone; PRL+ = Prolactinoma that expressed prolactin. ND = not tested.
