# Supplementary material for: Signaling pathway networks mined from human pituitary adenoma proteomics data
Source: BMC Med Genomics. 2010 Apr 28;3:13. doi: 10.1186/1755-8794-3-13 (PMC2884164; doi:10.1186/1755-8794-3-13)
Supplement: Additional file 2 — Supplementary Figures. This file contains supplementary figures S1-S4. Supplementary Figure S1 shows significant canonical pathways that are involved in human pituitary adenoma mapping proteomic data. Supplementary Figure S2 shows significant canonical pathways that are involved in human pituitary adenoma comparative proteomic data. Supplementary Figure S3 shows significant canonical pathways that are involved in human pituitary adenoma nitroproteomics data. Supplementary Figure S4 shows significant canonical pathways that are involved in human pituitary control nitroproteomics data. [file 1755-8794-3-13-S2.PPT]

## Slide 1
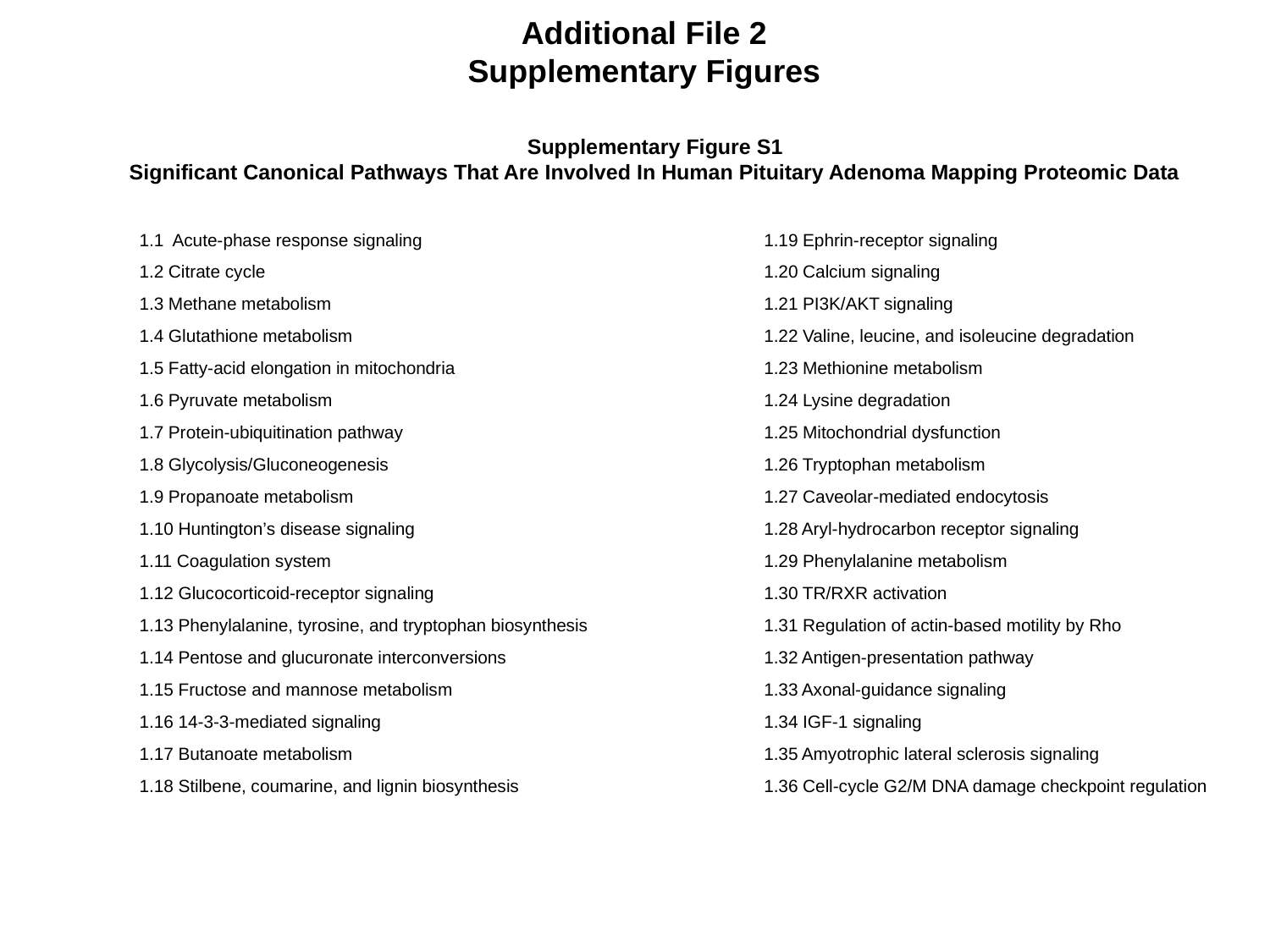

Additional File 2
Supplementary Figures
Supplementary Figure S1
Significant Canonical Pathways That Are Involved In Human Pituitary Adenoma Mapping Proteomic Data
1.1 Acute-phase response signaling
1.2 Citrate cycle
1.3 Methane metabolism
1.4 Glutathione metabolism
1.5 Fatty-acid elongation in mitochondria
1.6 Pyruvate metabolism
1.7 Protein-ubiquitination pathway
1.8 Glycolysis/Gluconeogenesis
1.9 Propanoate metabolism
1.10 Huntington’s disease signaling
1.11 Coagulation system
1.12 Glucocorticoid-receptor signaling
1.13 Phenylalanine, tyrosine, and tryptophan biosynthesis
1.14 Pentose and glucuronate interconversions
1.15 Fructose and mannose metabolism
1.16 14-3-3-mediated signaling
1.17 Butanoate metabolism
1.18 Stilbene, coumarine, and lignin biosynthesis
1.19 Ephrin-receptor signaling
1.20 Calcium signaling
1.21 PI3K/AKT signaling
1.22 Valine, leucine, and isoleucine degradation
1.23 Methionine metabolism
1.24 Lysine degradation
1.25 Mitochondrial dysfunction
1.26 Tryptophan metabolism
1.27 Caveolar-mediated endocytosis
1.28 Aryl-hydrocarbon receptor signaling
1.29 Phenylalanine metabolism
1.30 TR/RXR activation
1.31 Regulation of actin-based motility by Rho
1.32 Antigen-presentation pathway
1.33 Axonal-guidance signaling
1.34 IGF-1 signaling
1.35 Amyotrophic lateral sclerosis signaling
1.36 Cell-cycle G2/M DNA damage checkpoint regulation

## Slide 2
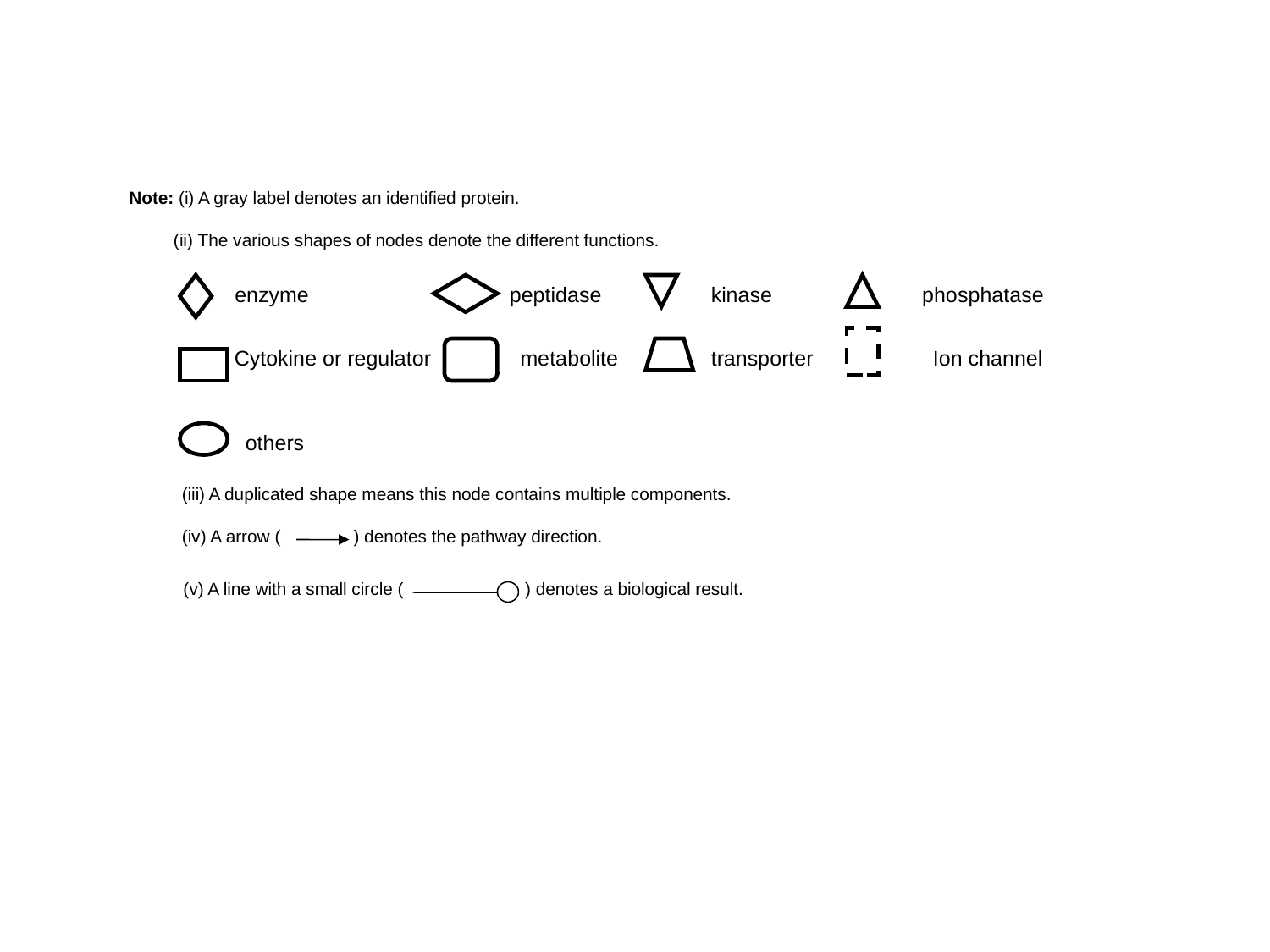

Note: (i) A gray label denotes an identified protein.
 (ii) The various shapes of nodes denote the different functions.
enzyme
peptidase
kinase
phosphatase
Cytokine or regulator
metabolite
transporter
Ion channel
others
(iii) A duplicated shape means this node contains multiple components.
(iv) A arrow ( ) denotes the pathway direction.
(v) A line with a small circle ( ) denotes a biological result.

## Slide 3
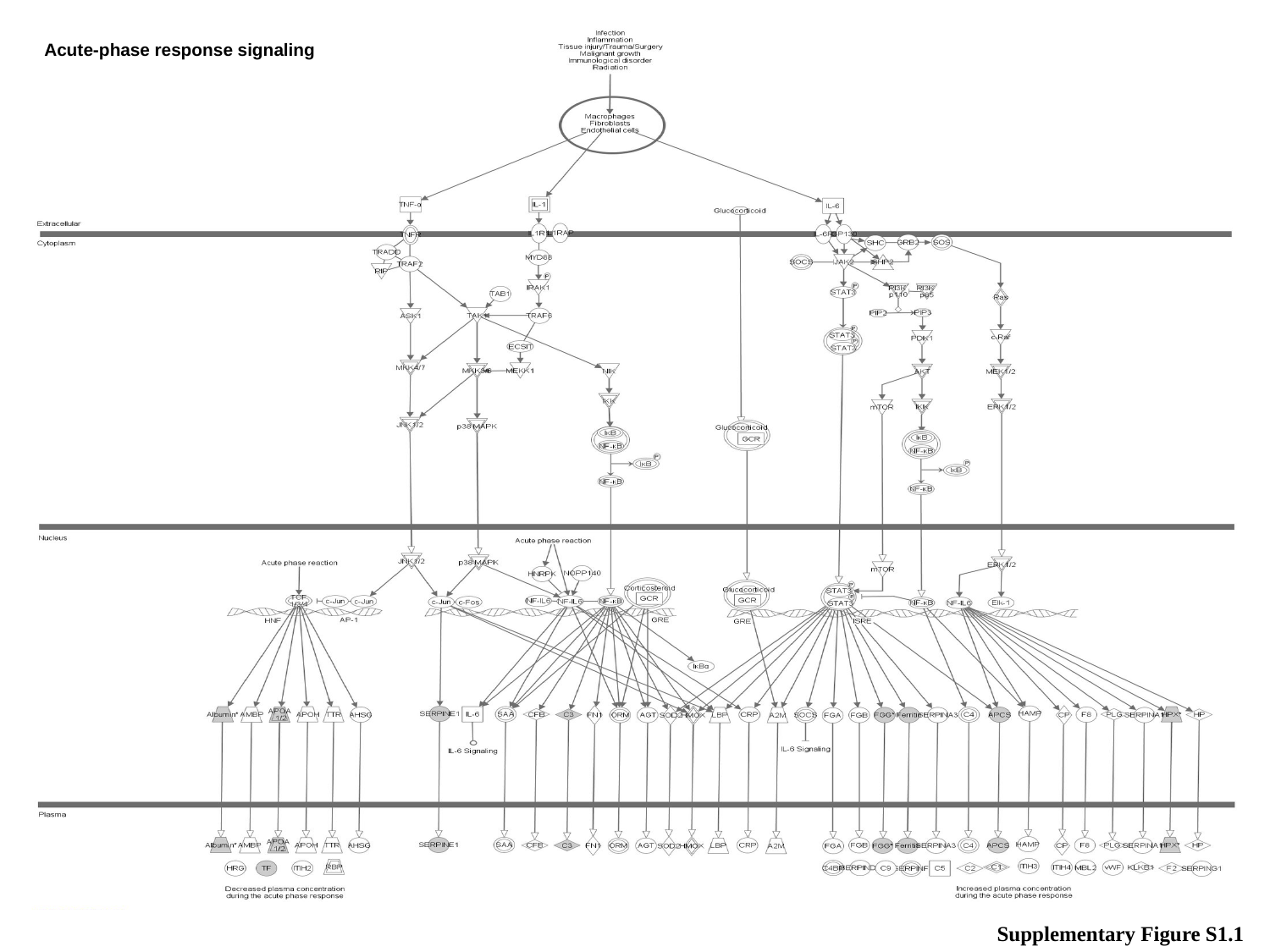

Acute-phase response signaling
Supplementary Figure S1.1

## Slide 4
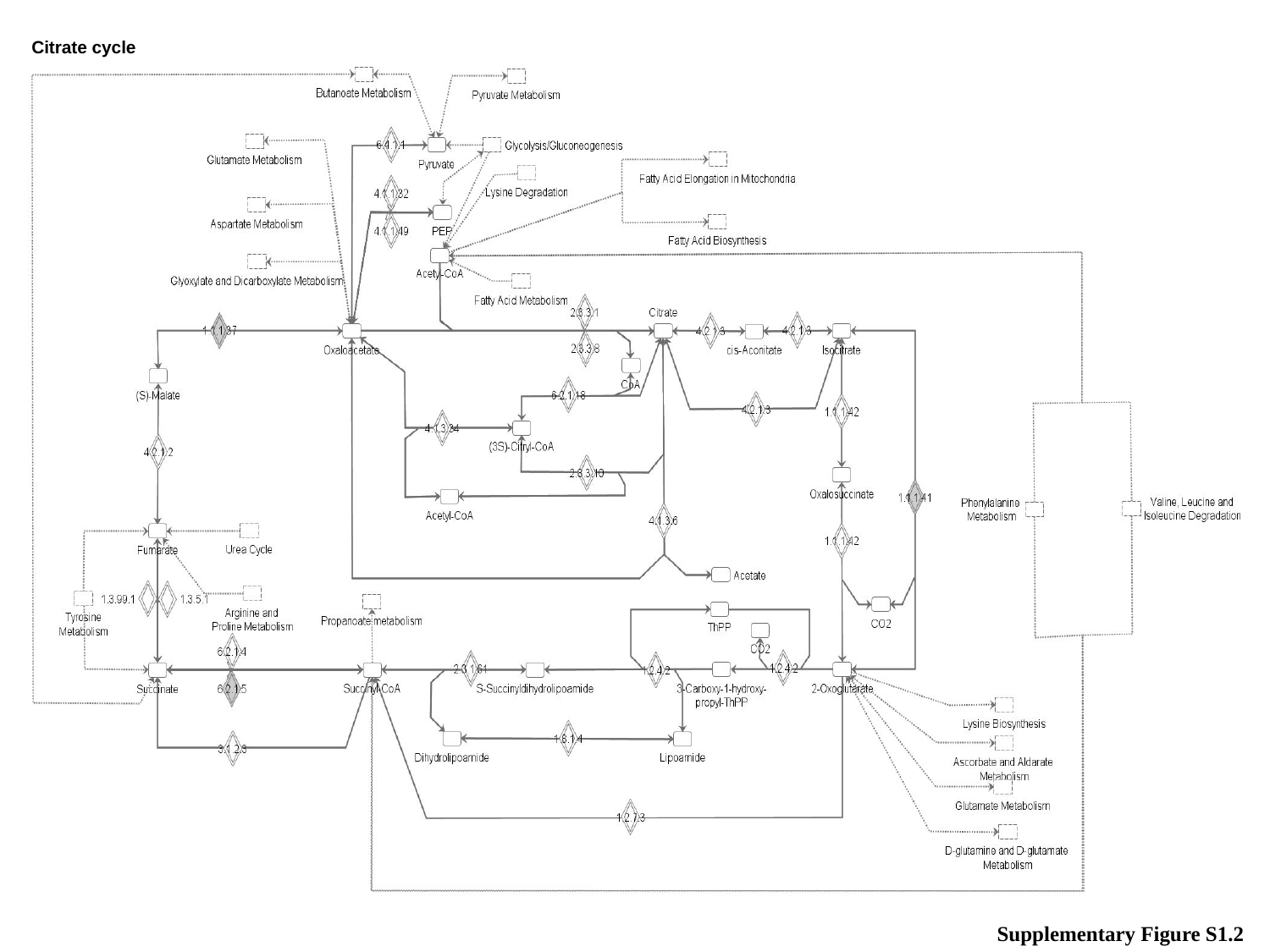

Citrate cycle
Supplementary Figure S1.2

## Slide 5
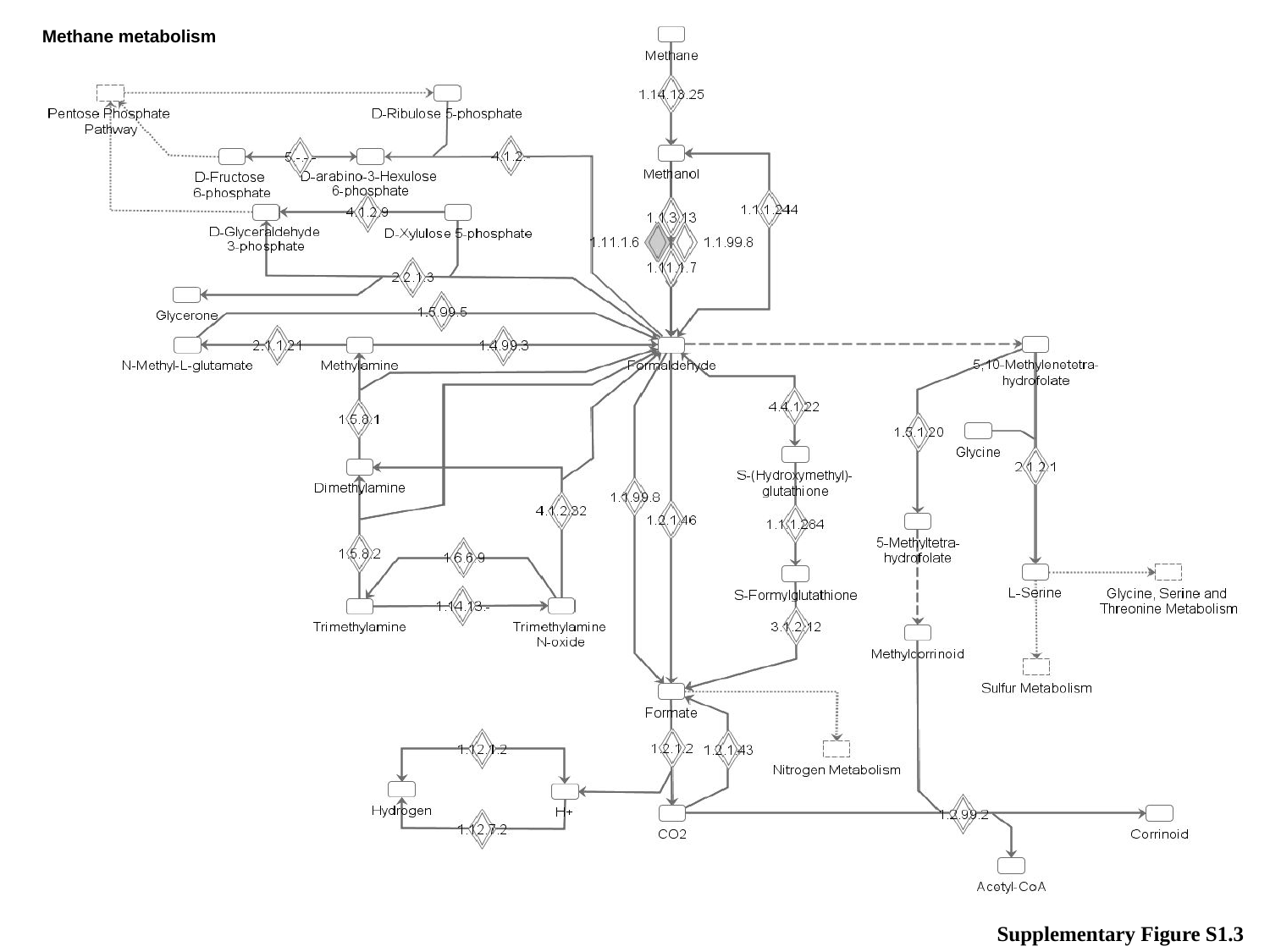

Methane metabolism
Supplementary Figure S1.3

## Slide 6
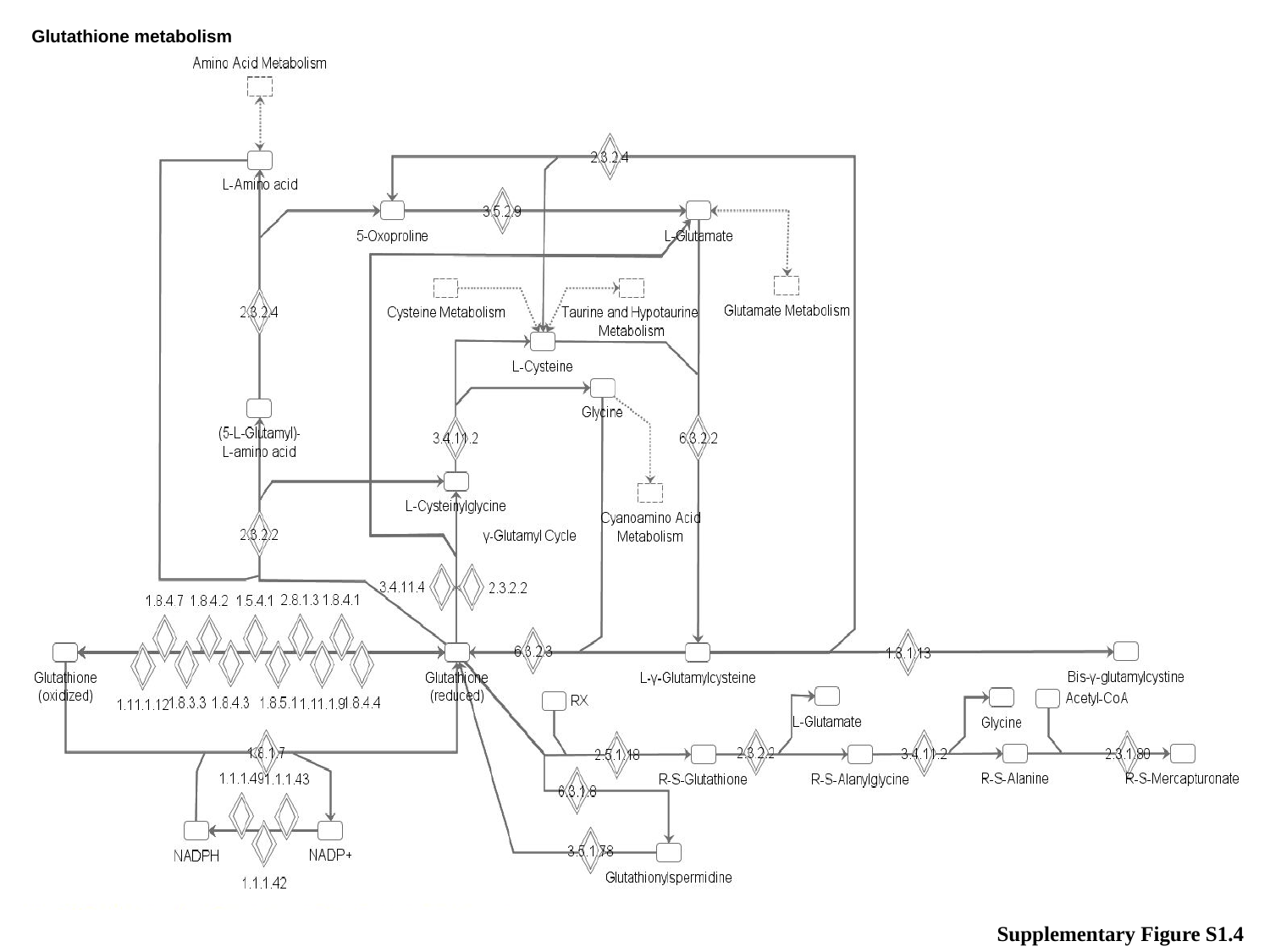

Glutathione metabolism
Supplementary Figure S1.4

## Slide 7
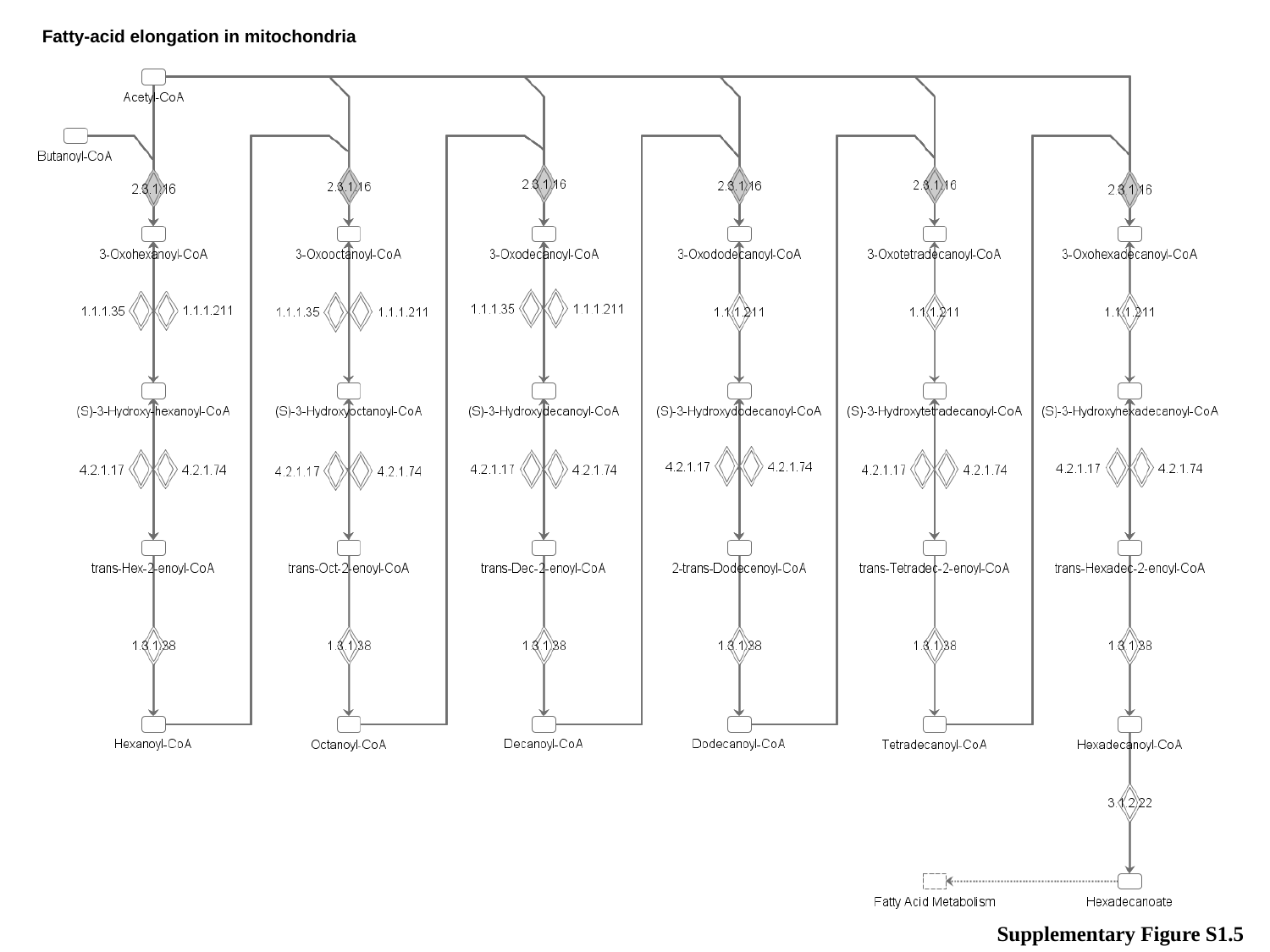

Fatty-acid elongation in mitochondria
Supplementary Figure S1.5

## Slide 8
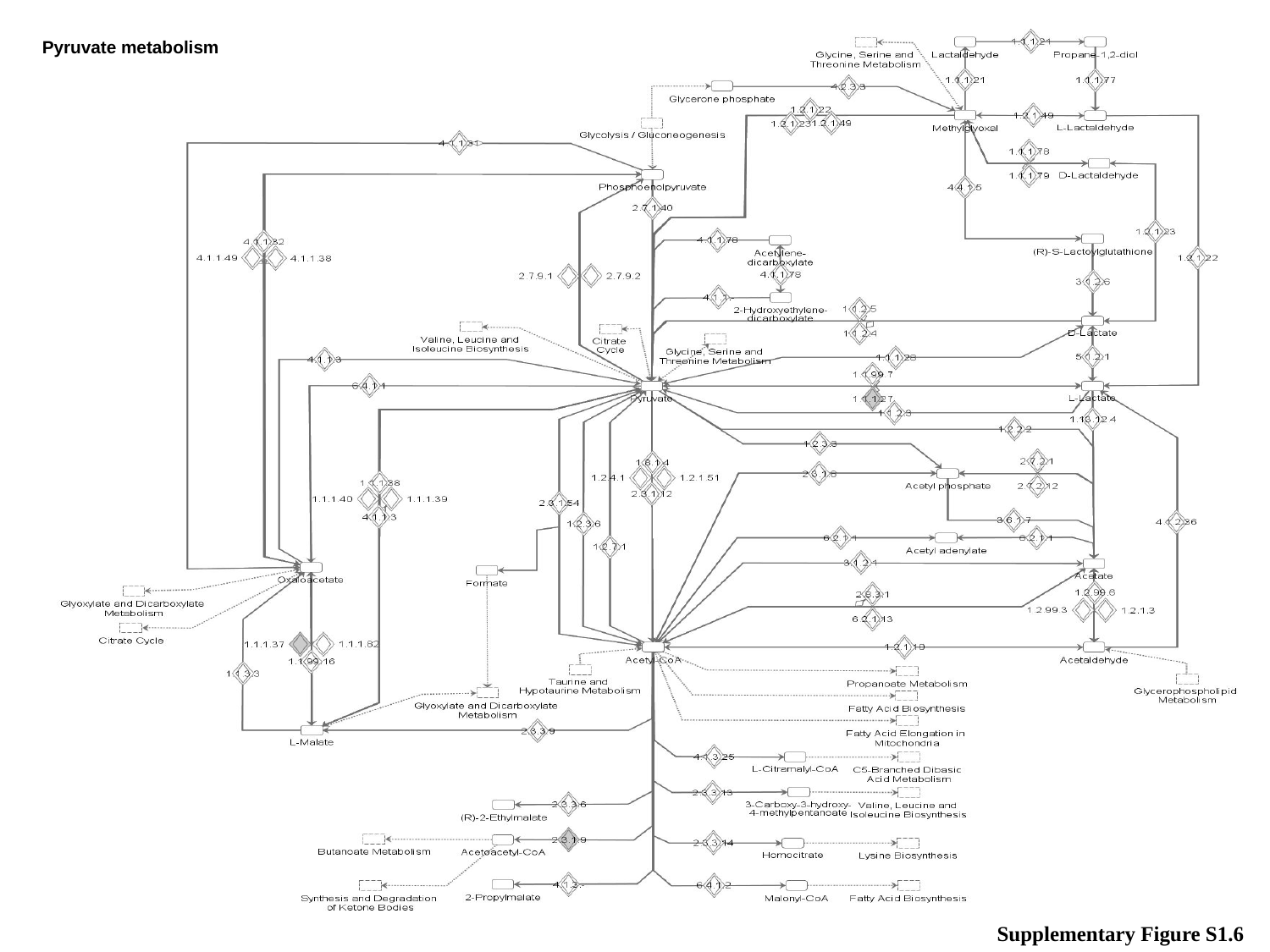

Pyruvate metabolism
Supplementary Figure S1.6

## Slide 9
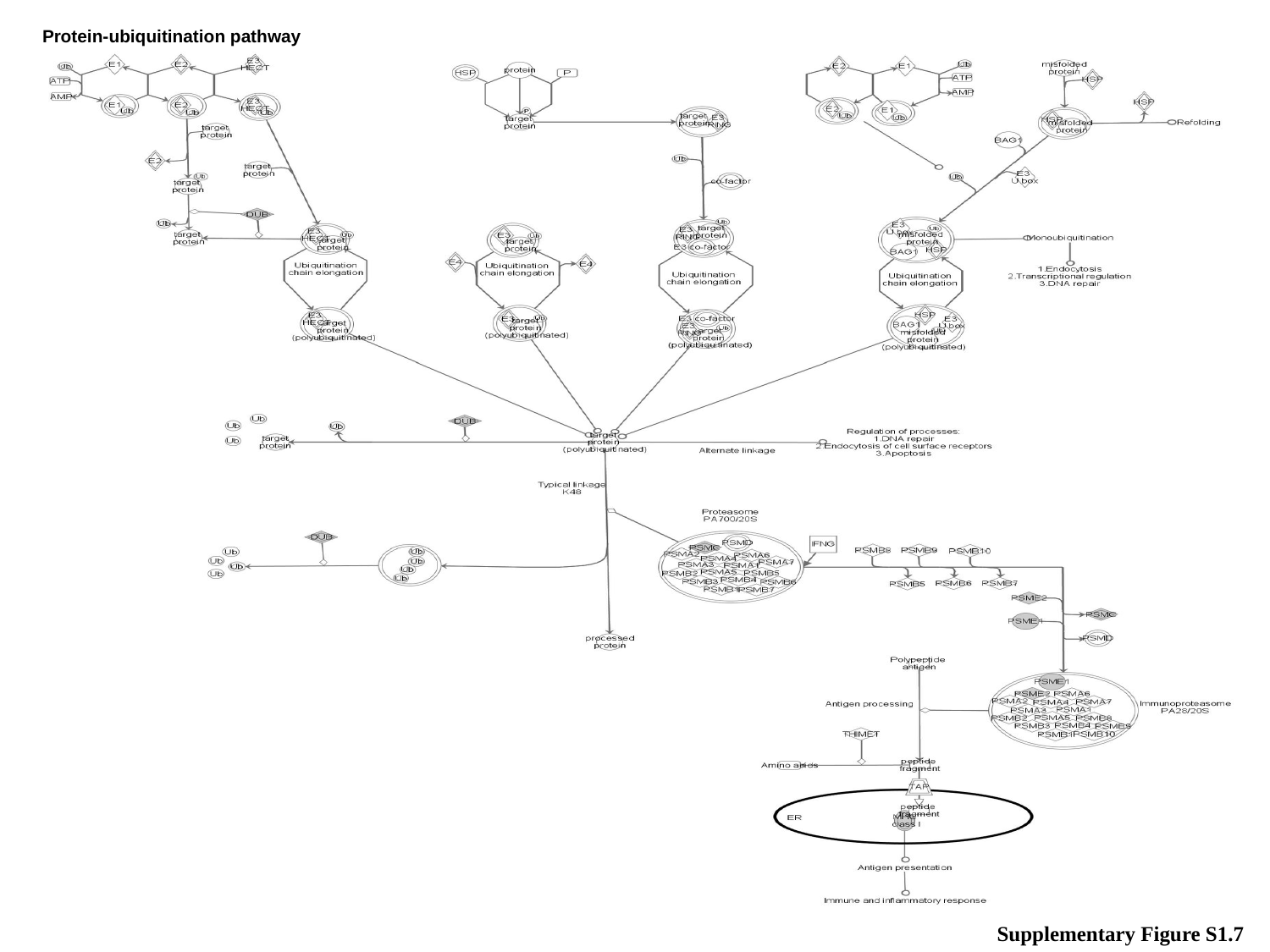

Protein-ubiquitination pathway
Supplementary Figure S1.7

## Slide 10
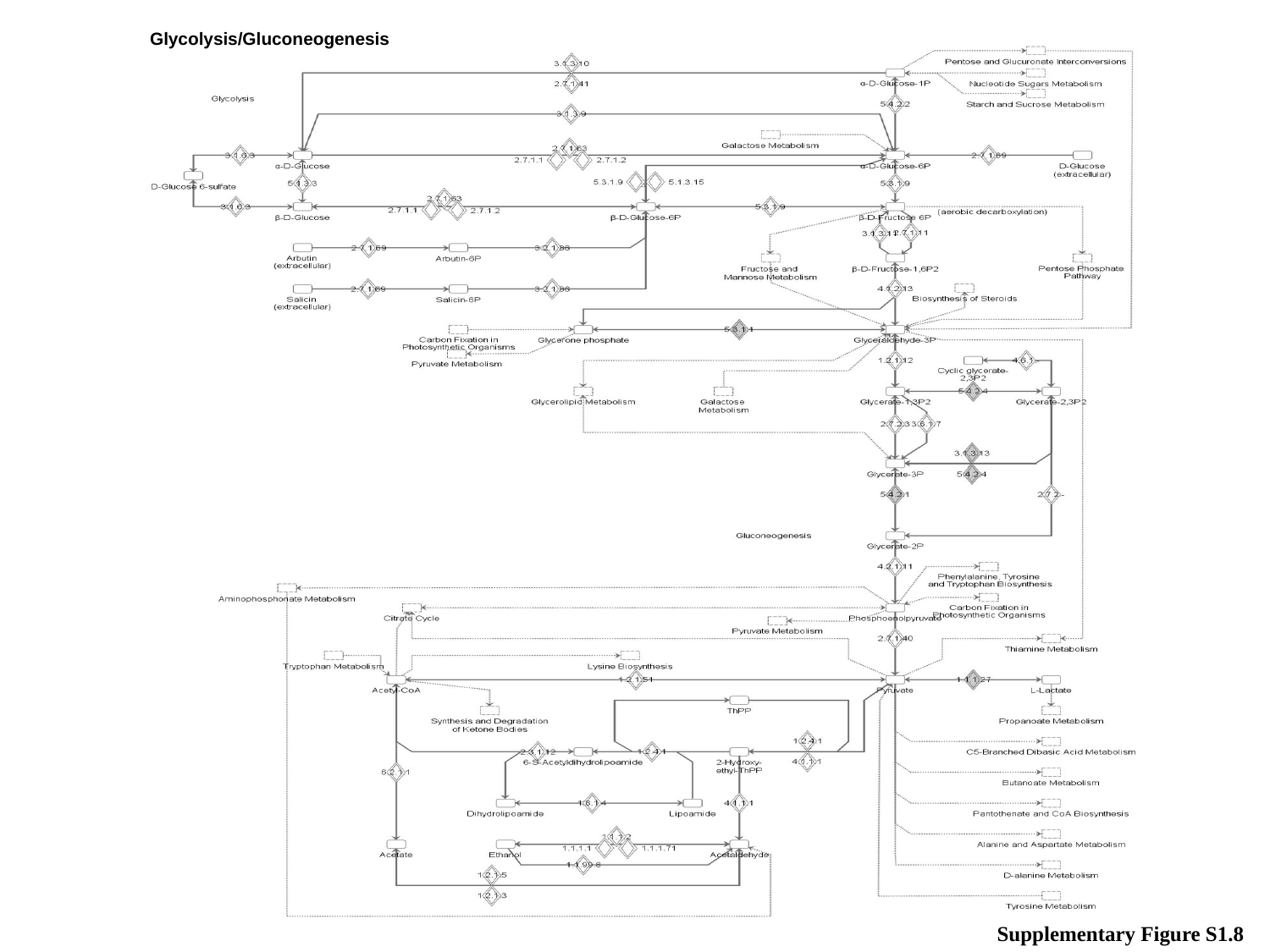

Glycolysis/Gluconeogenesis
Supplementary Figure S1.8

## Slide 11
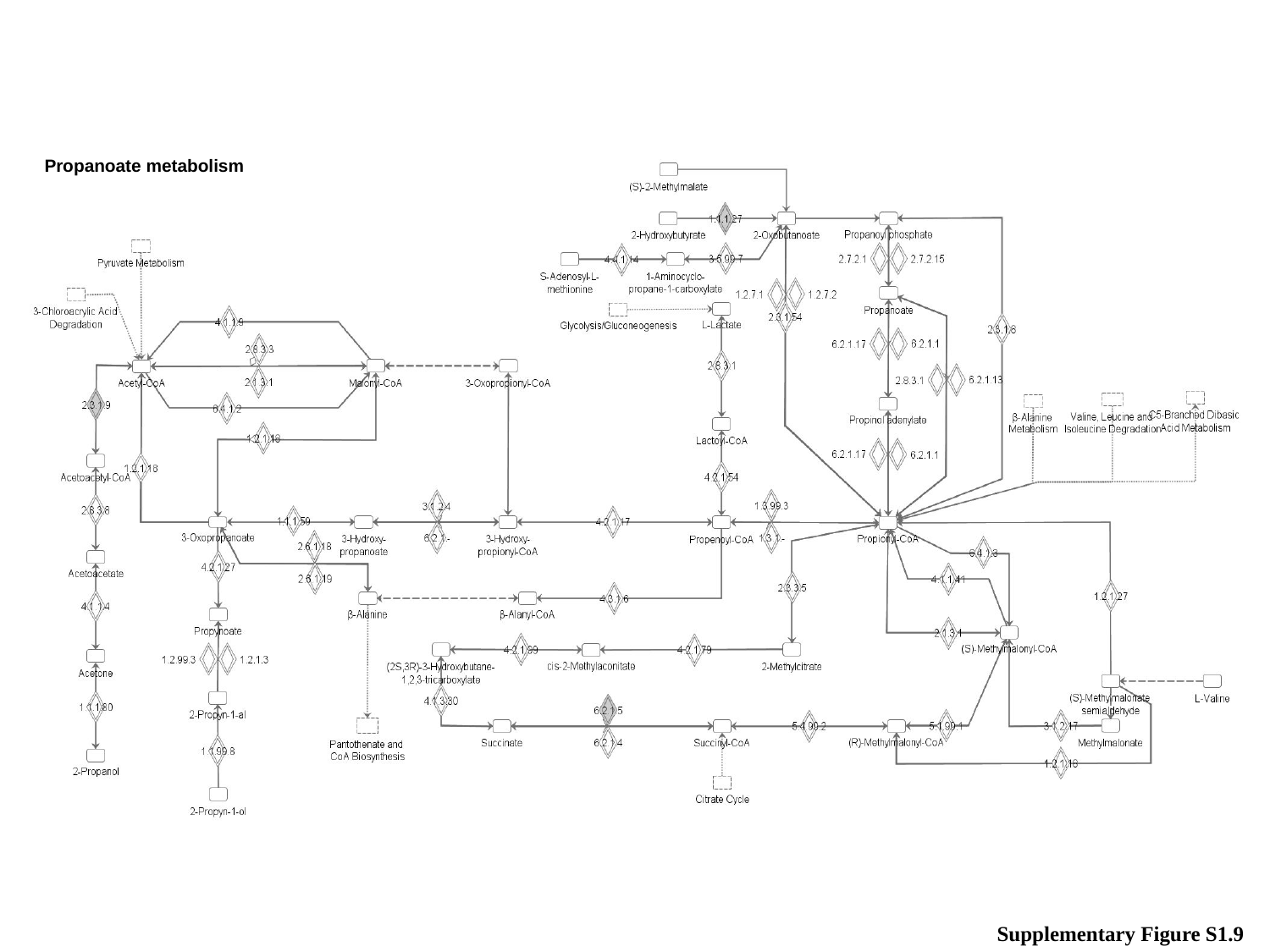

Propanoate metabolism
Supplementary Figure S1.9

## Slide 12
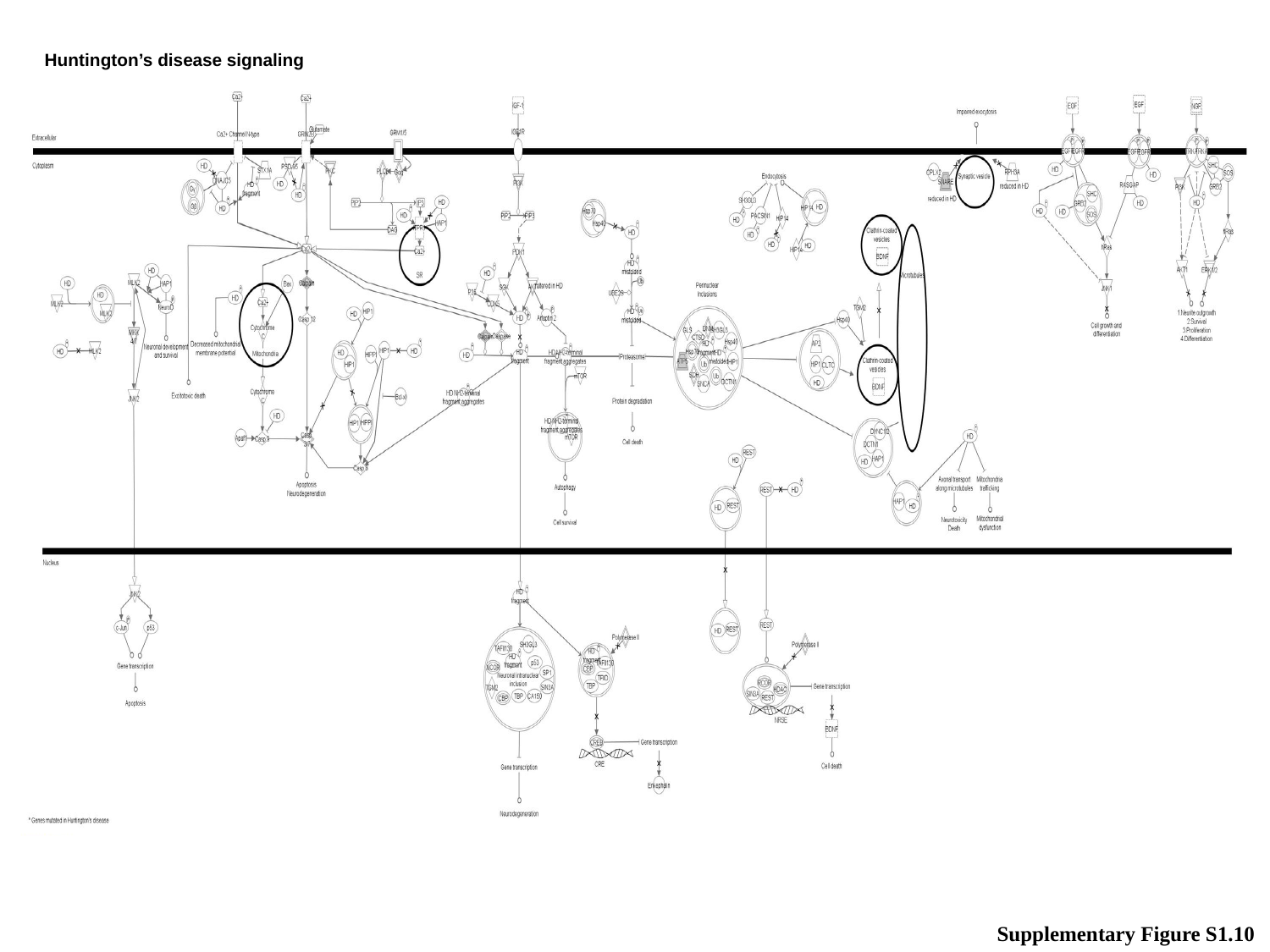

Huntington’s disease signaling
Supplementary Figure S1.10

## Slide 13
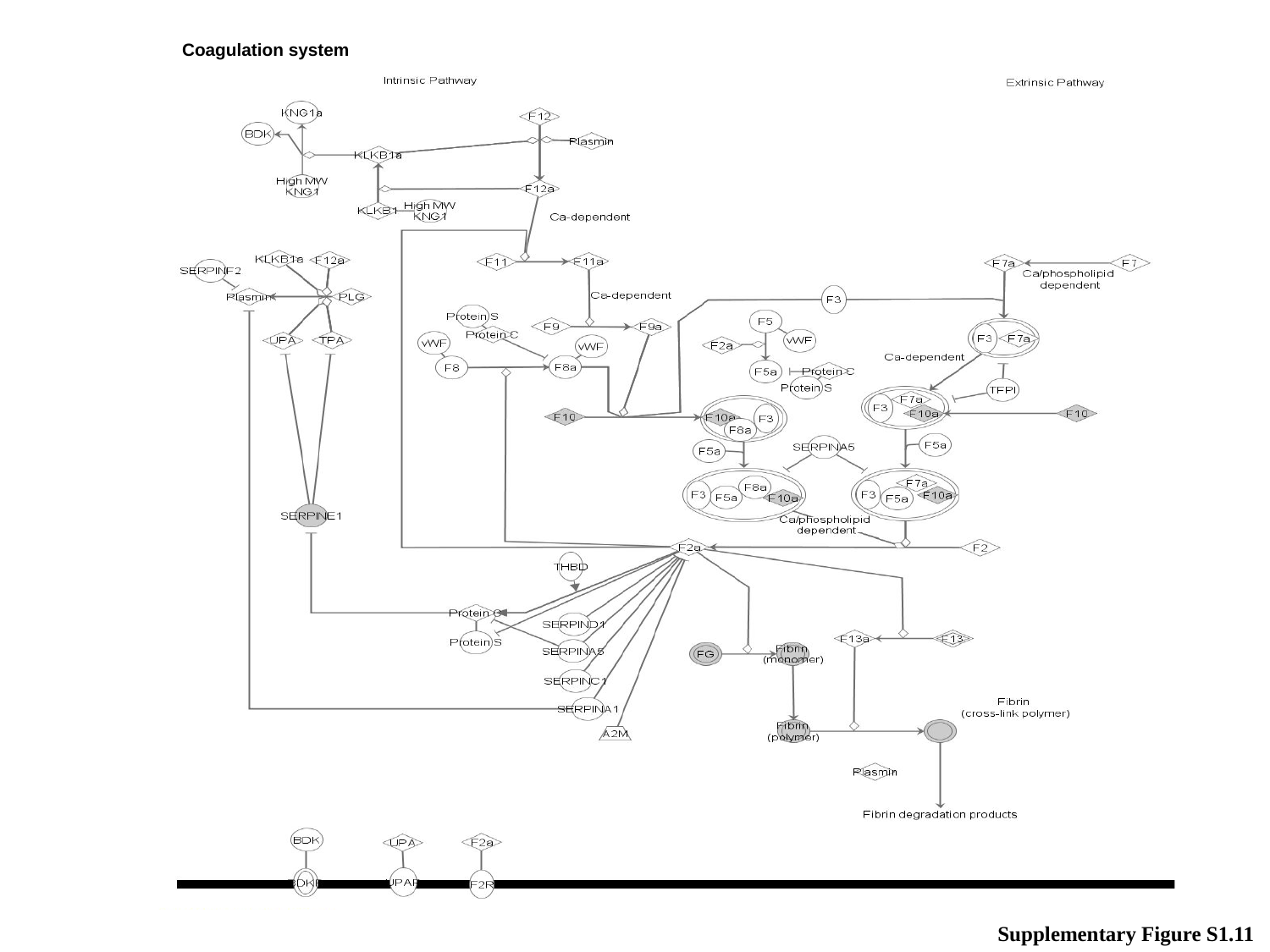

Coagulation system
Supplementary Figure S1.11

## Slide 14
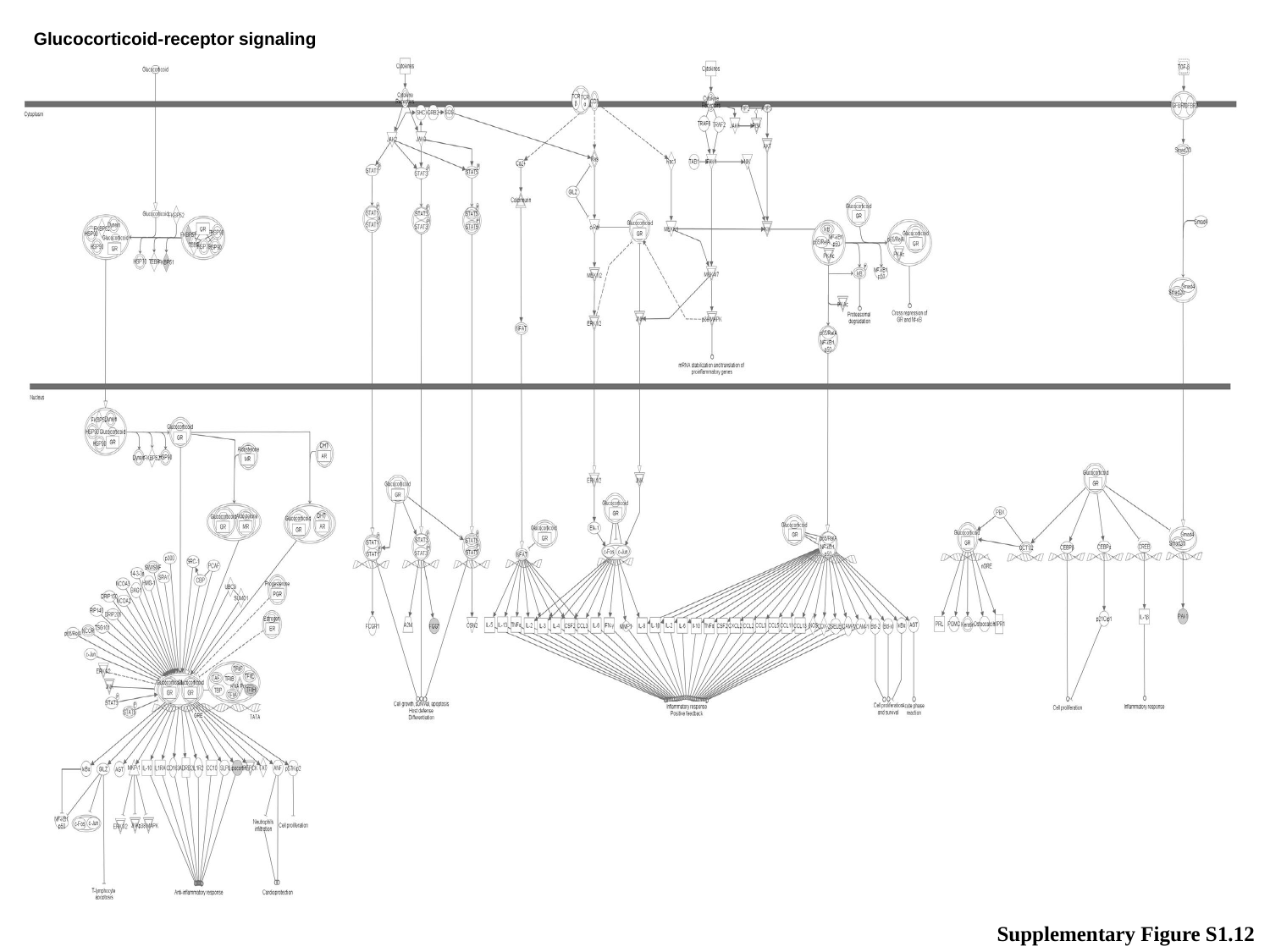

Glucocorticoid-receptor signaling
Supplementary Figure S1.12

## Slide 15
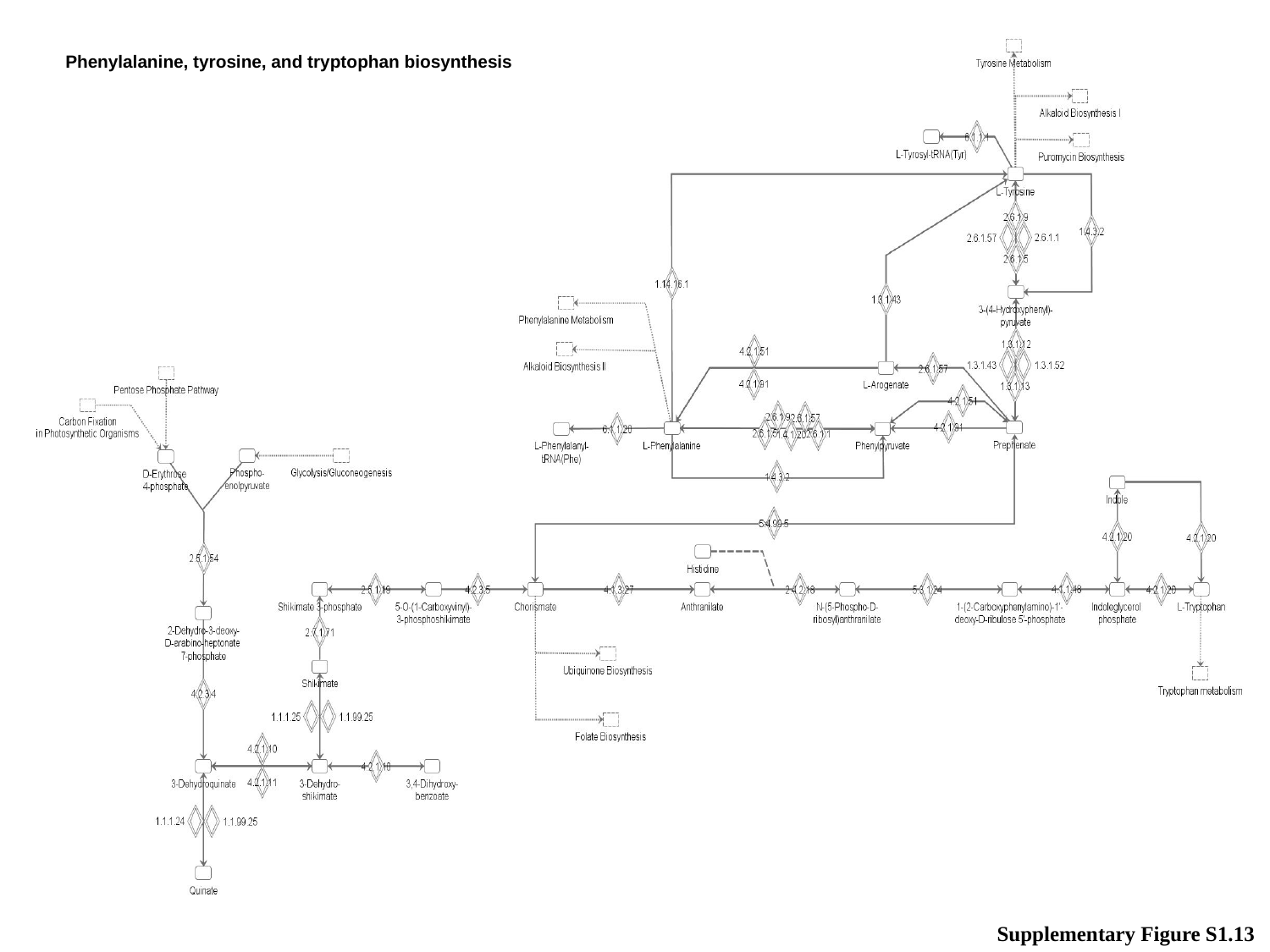

Phenylalanine, tyrosine, and tryptophan biosynthesis
Supplementary Figure S1.13

## Slide 16
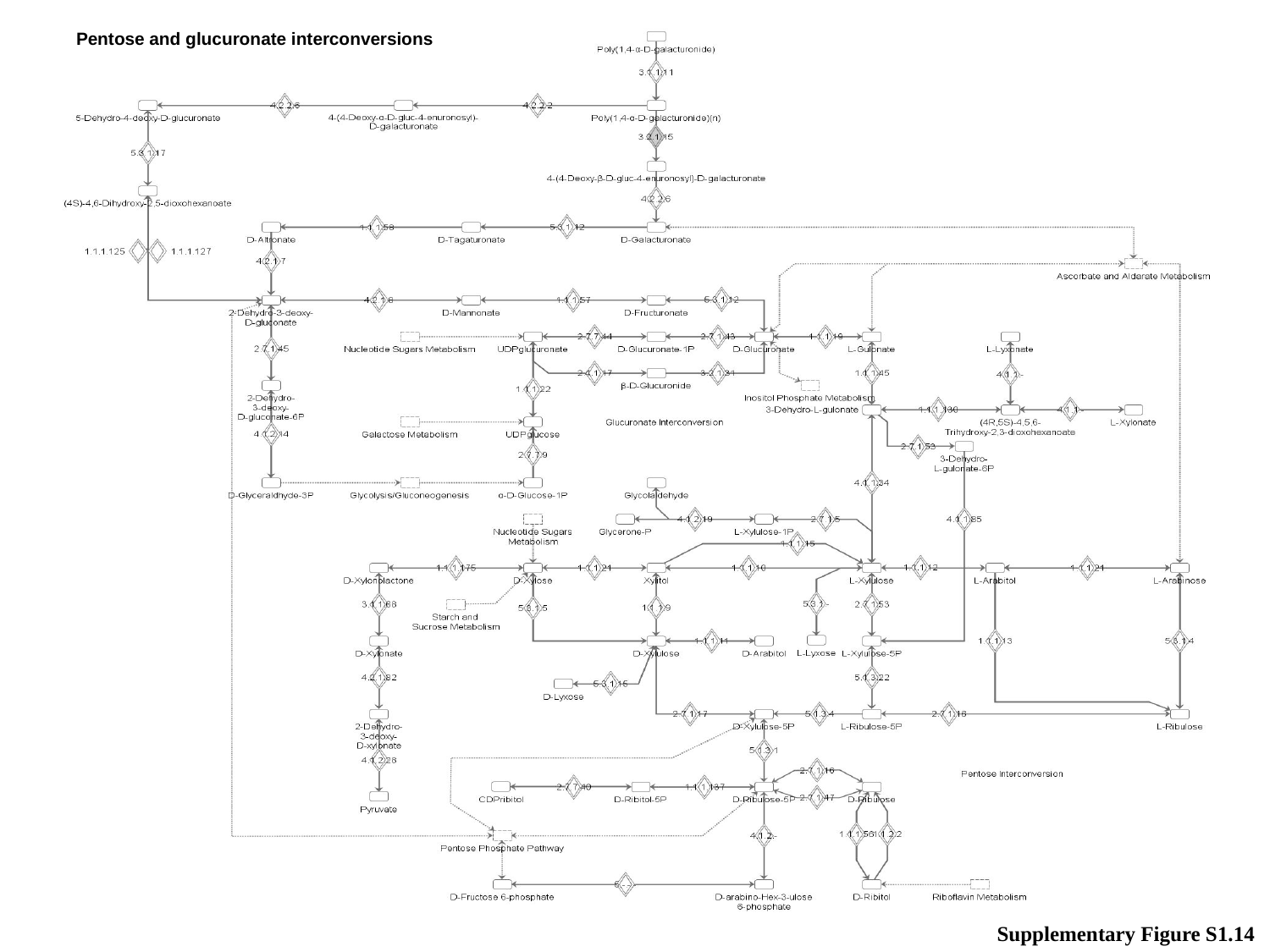

Pentose and glucuronate interconversions
Supplementary Figure S1.14

## Slide 17
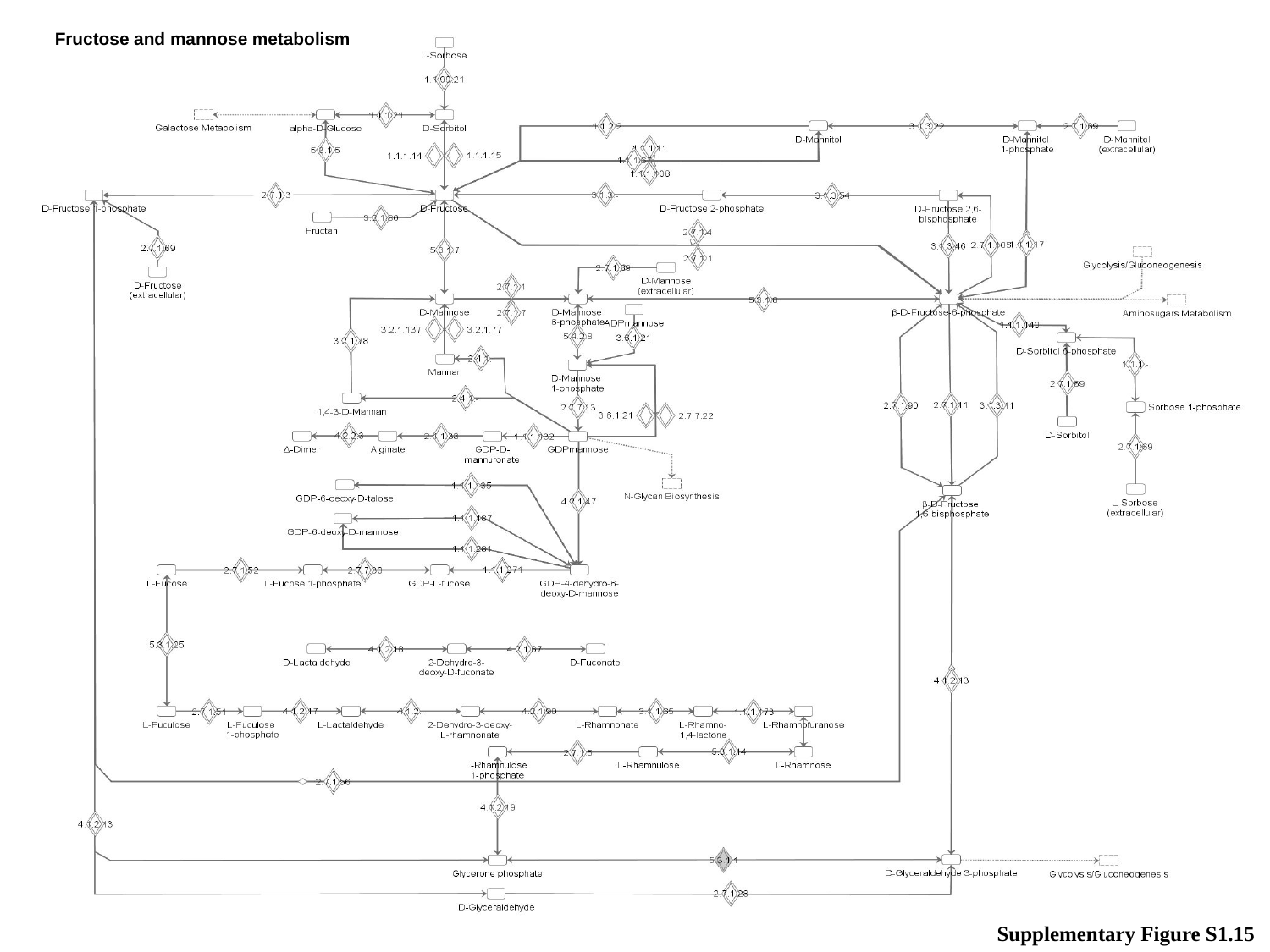

Fructose and mannose metabolism
Supplementary Figure S1.15

## Slide 18
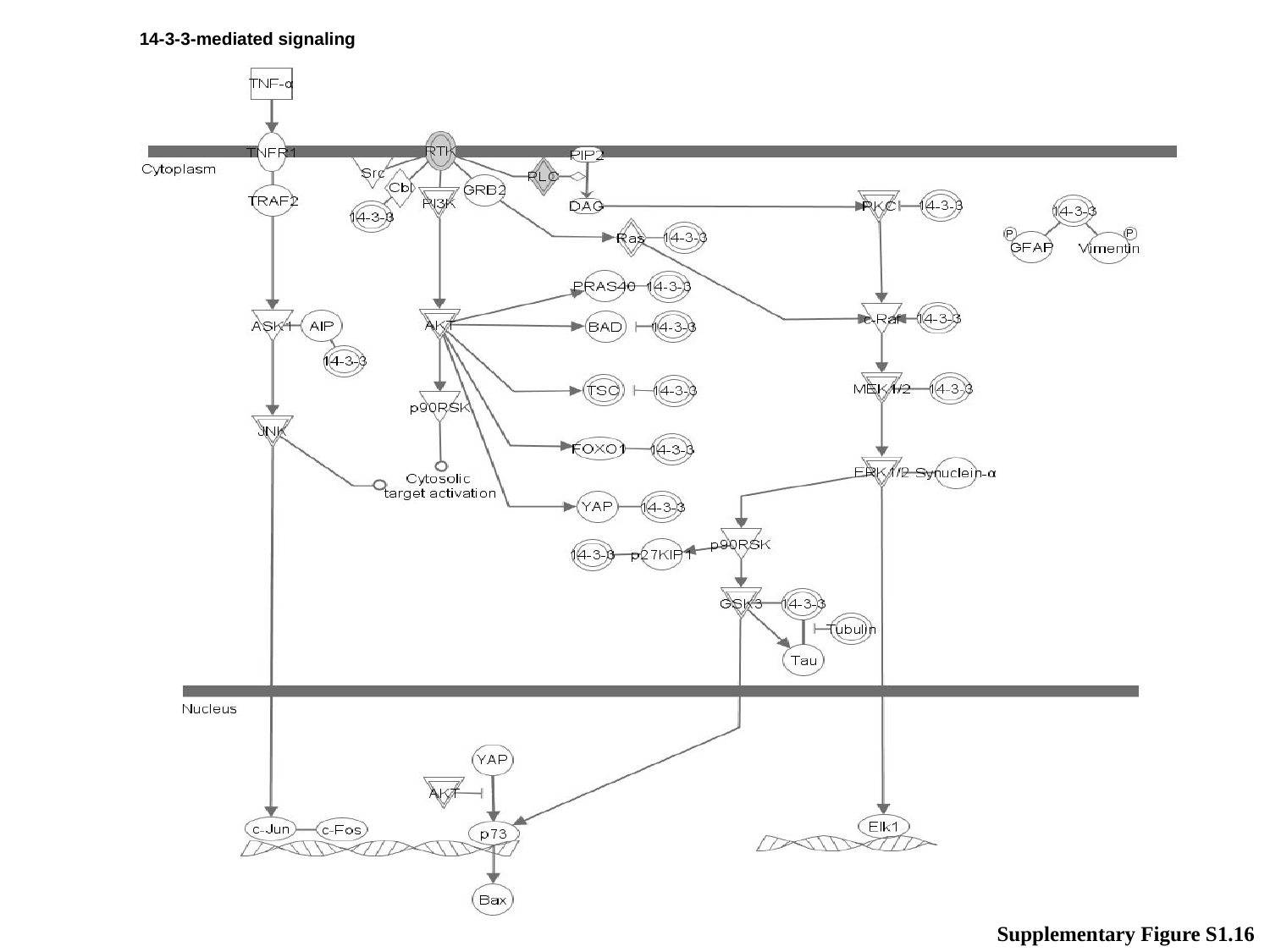

14-3-3-mediated signaling
Supplementary Figure S1.16

## Slide 19
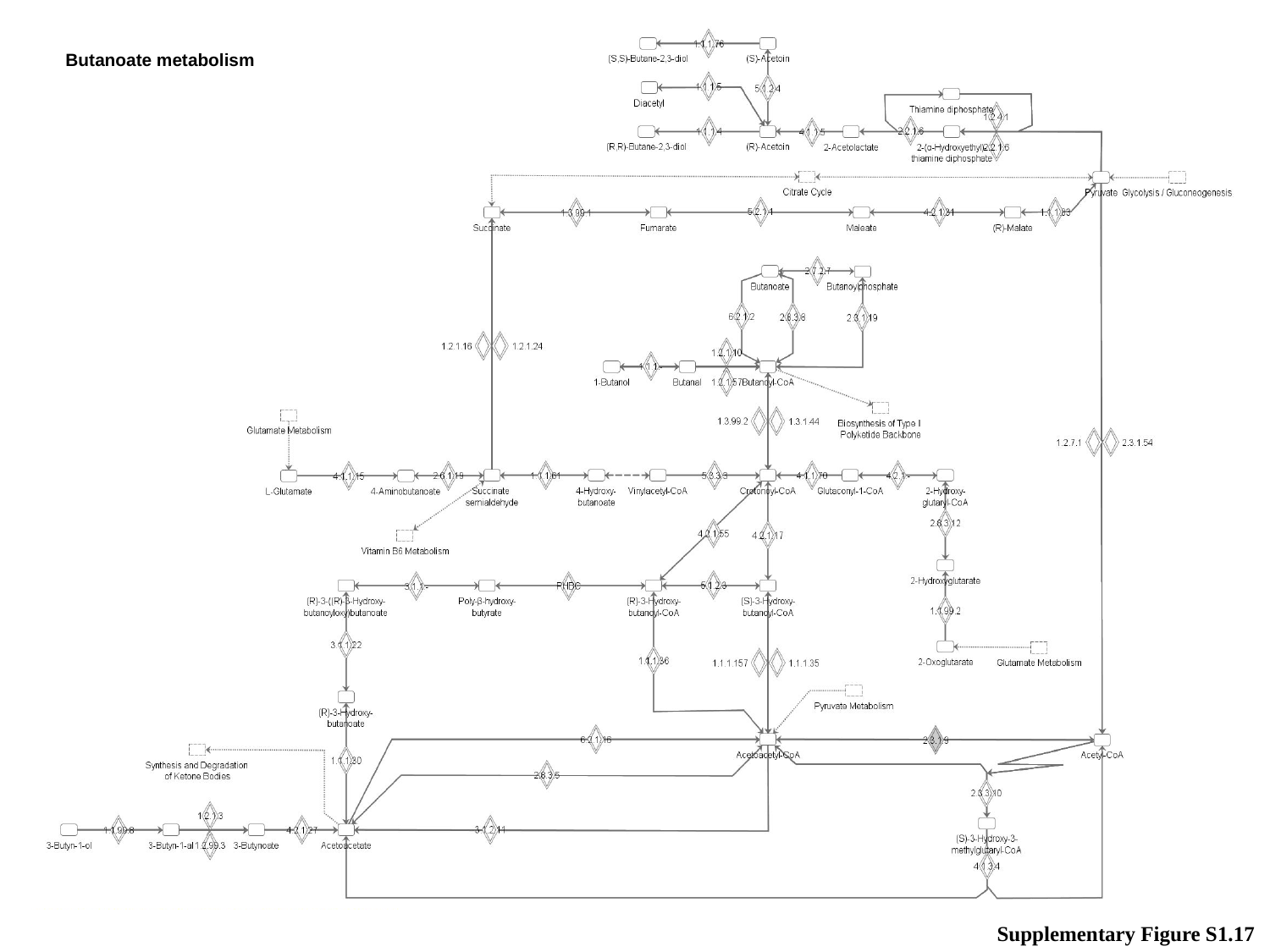

Butanoate metabolism
Supplementary Figure S1.17

## Slide 20
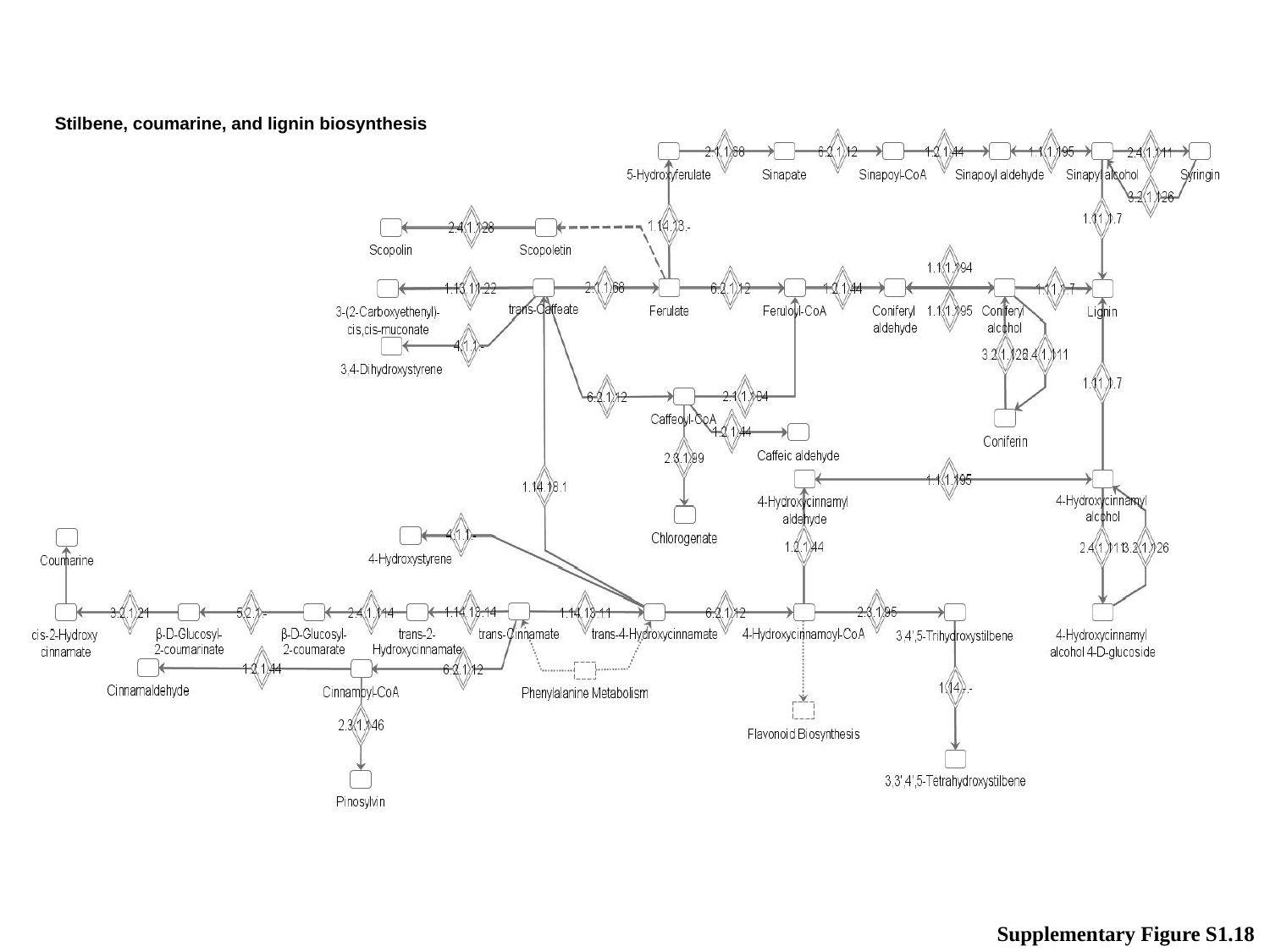

Stilbene, coumarine, and lignin biosynthesis
Supplementary Figure S1.18

## Slide 21
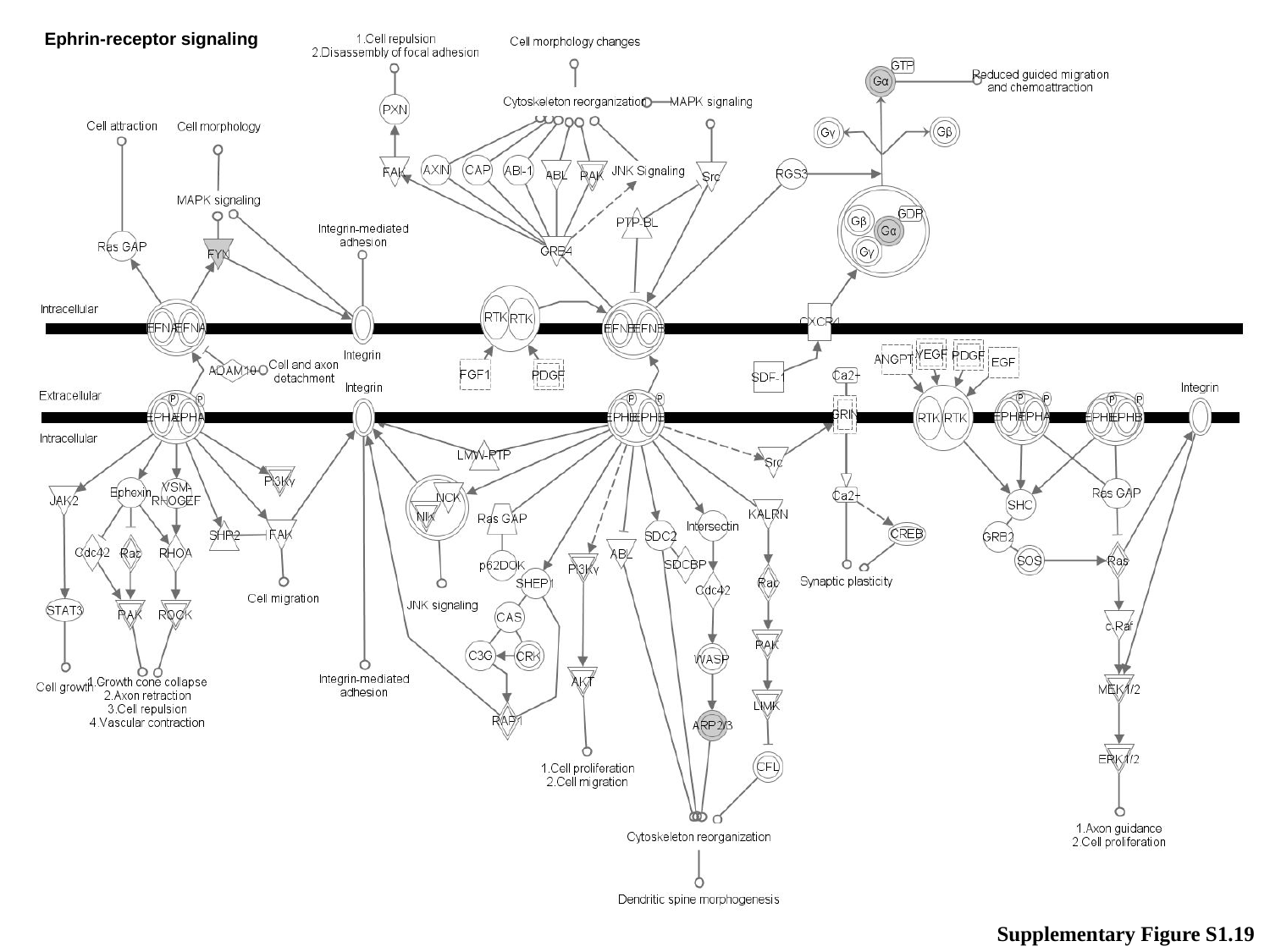

Ephrin-receptor signaling
Supplementary Figure S1.19

## Slide 22
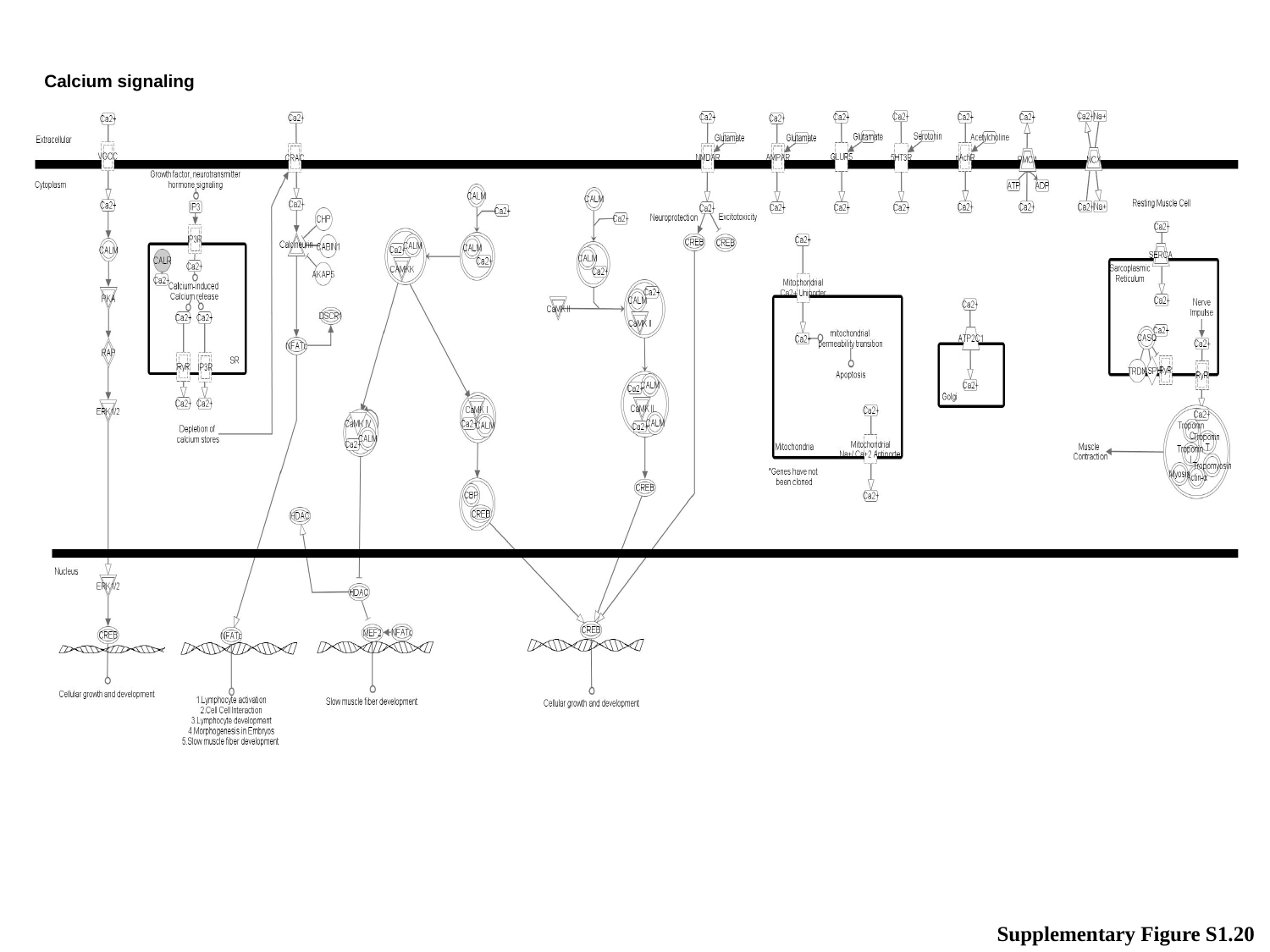

Calcium signaling
Supplementary Figure S1.20

## Slide 23
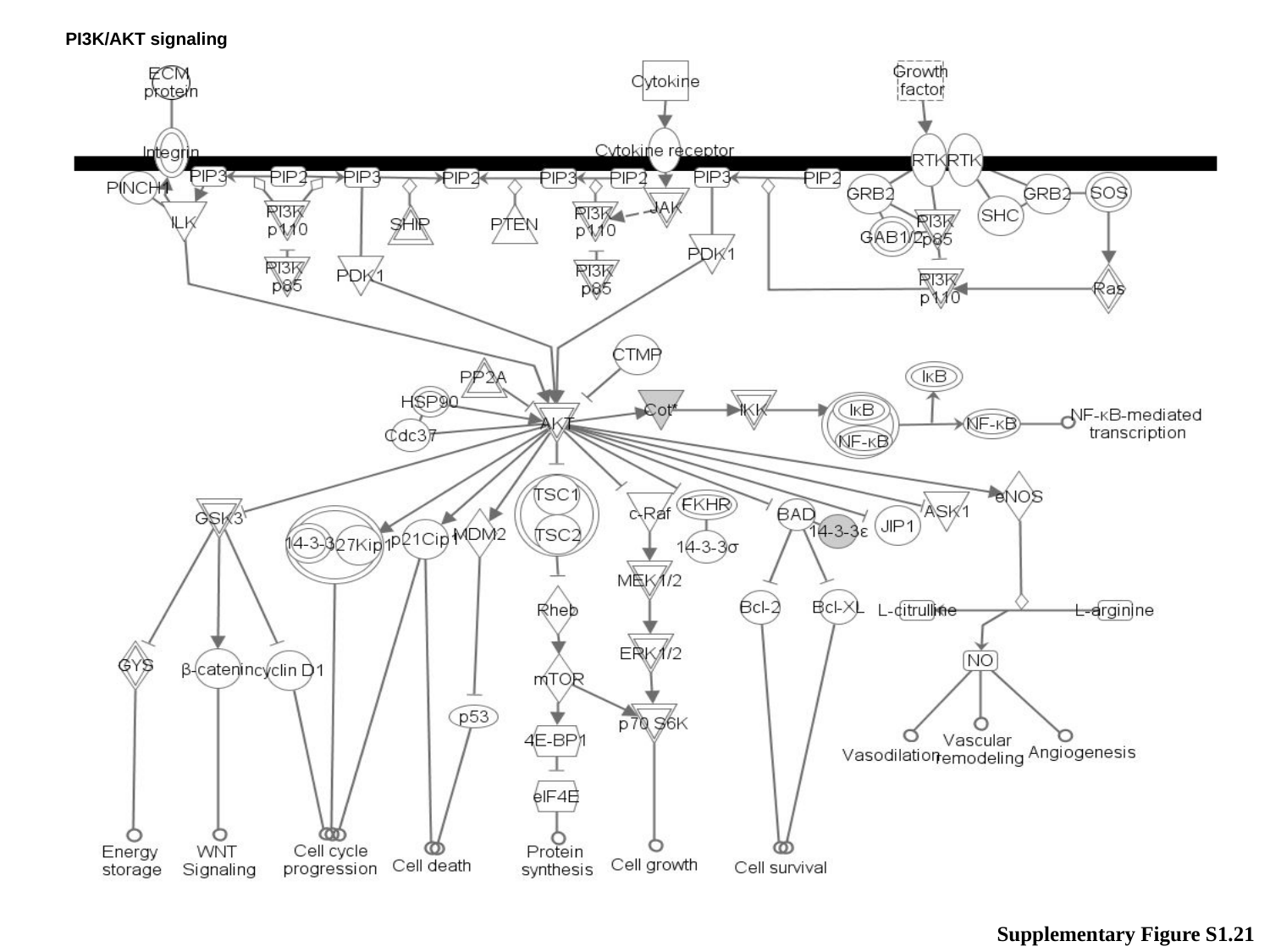

PI3K/AKT signaling
Supplementary Figure S1.21

## Slide 24
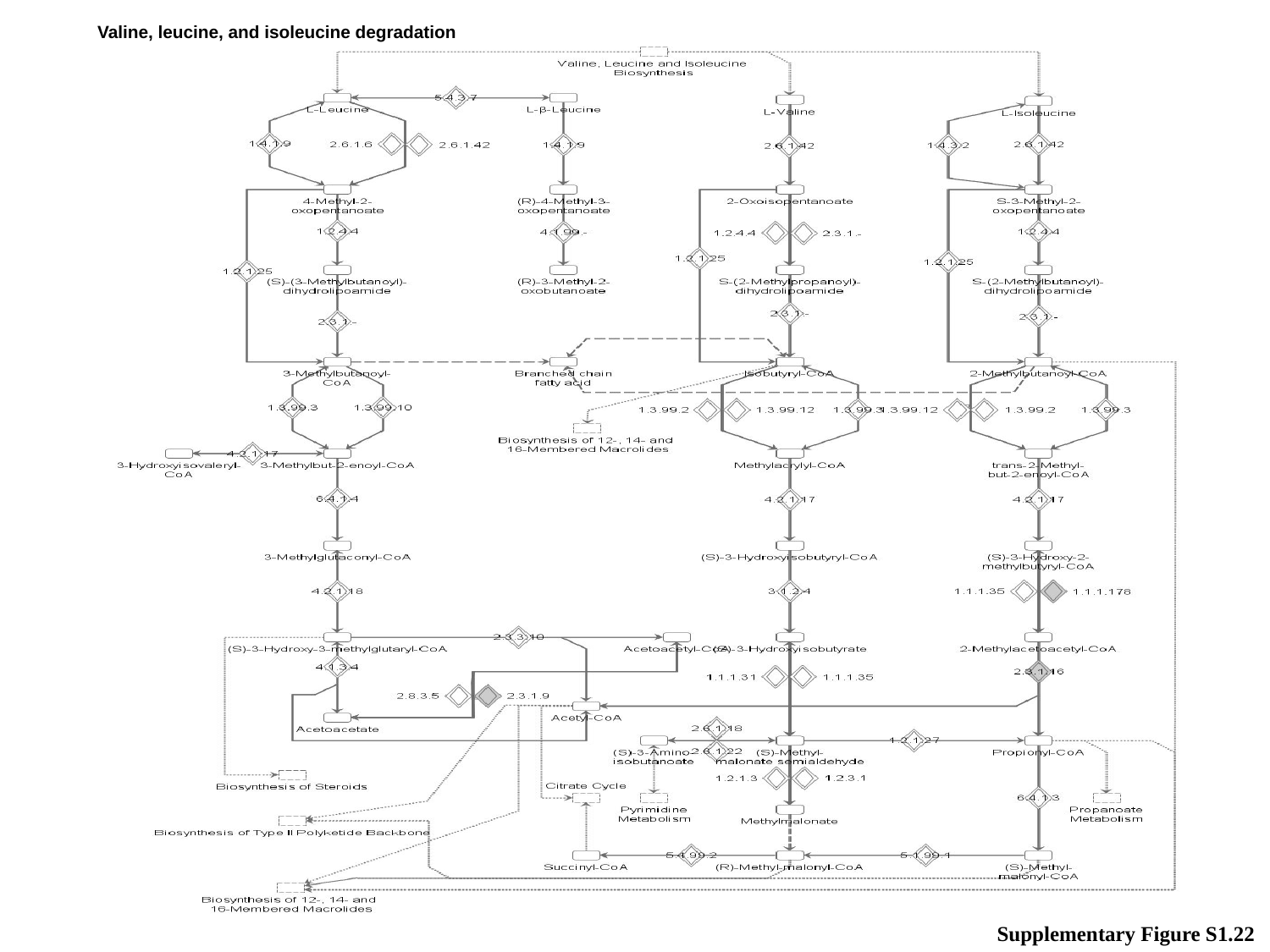

Valine, leucine, and isoleucine degradation
Supplementary Figure S1.22

## Slide 25
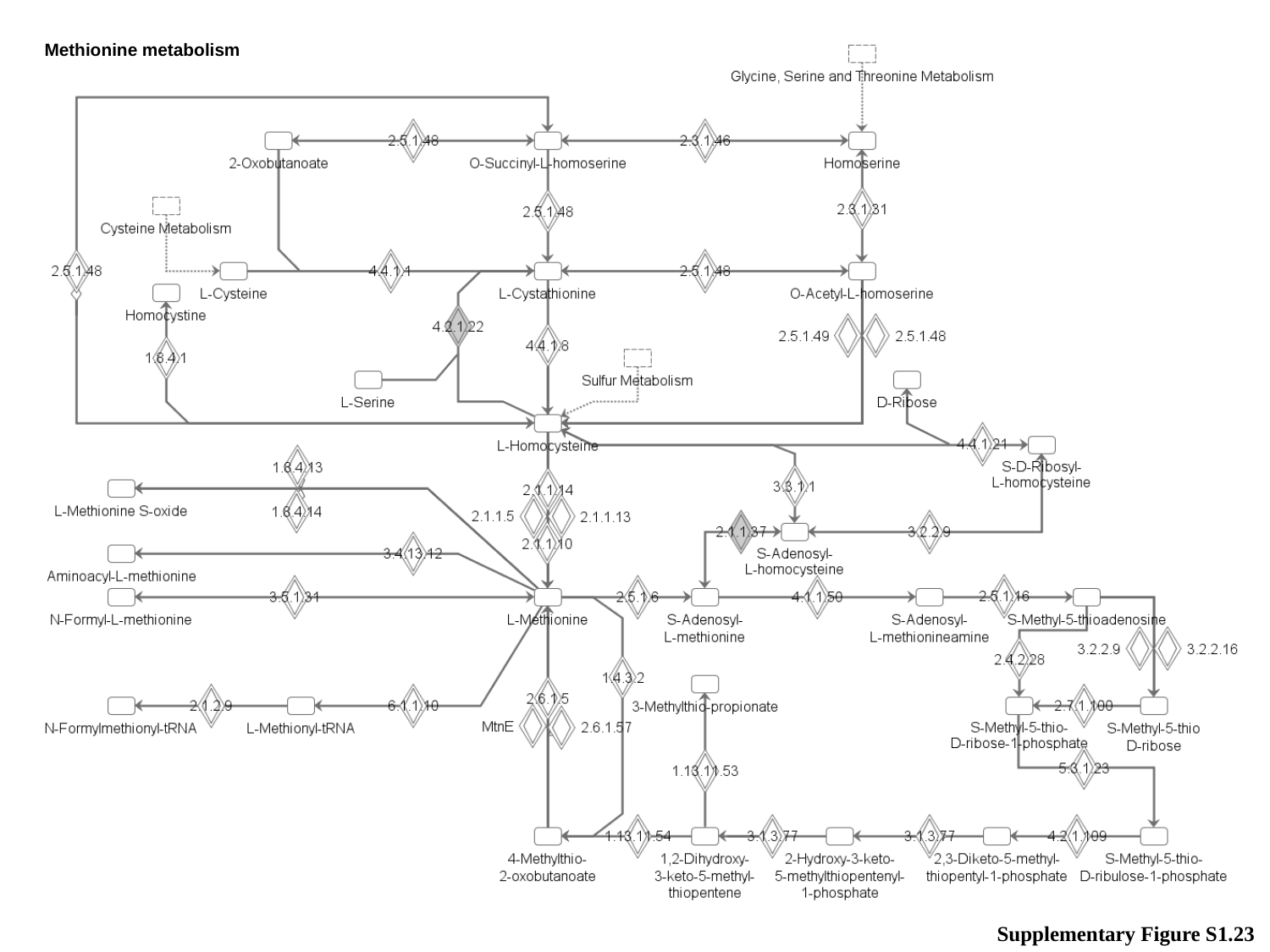

Methionine metabolism
Supplementary Figure S1.23

## Slide 26
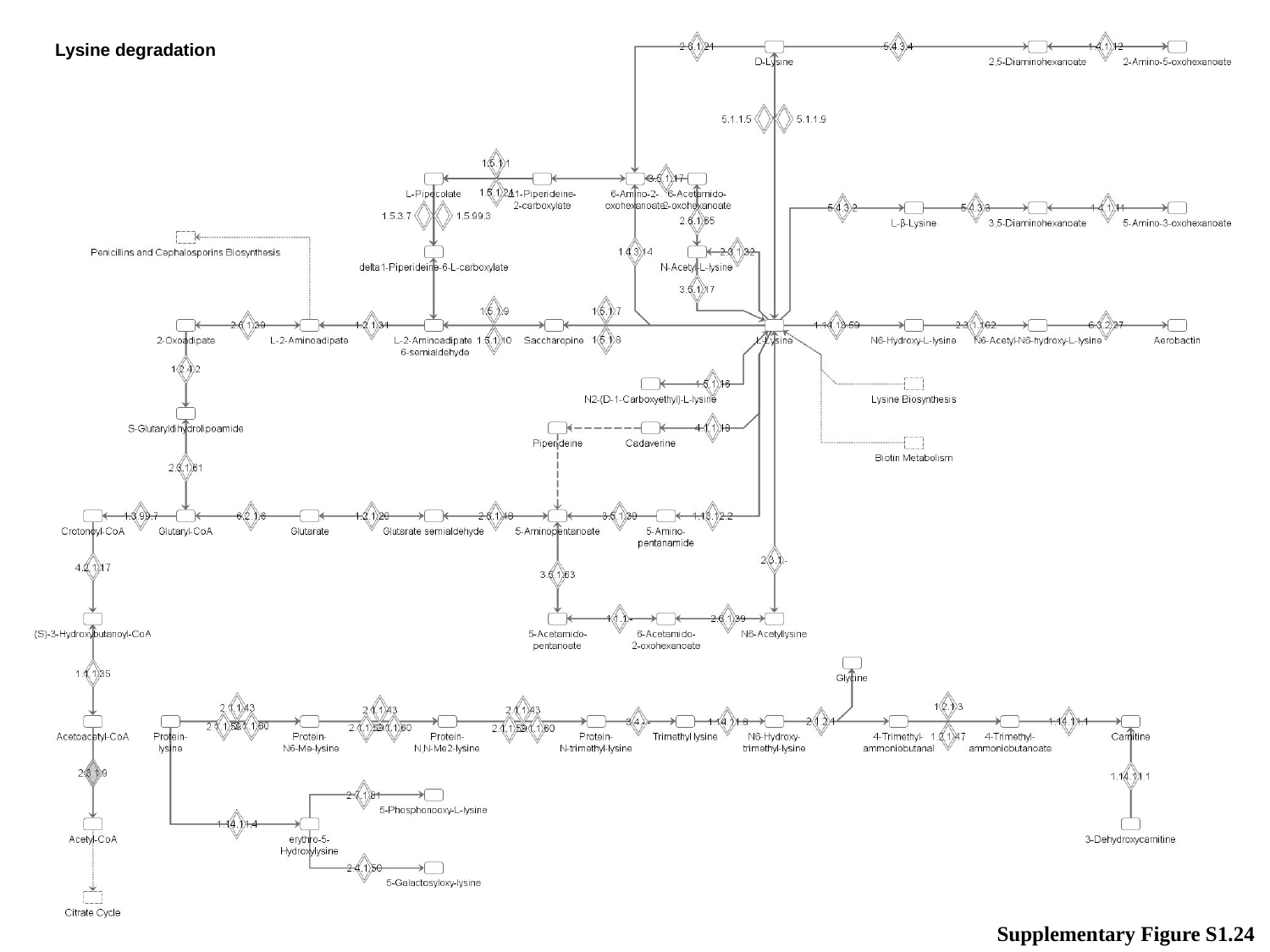

Lysine degradation
Supplementary Figure S1.24

## Slide 27
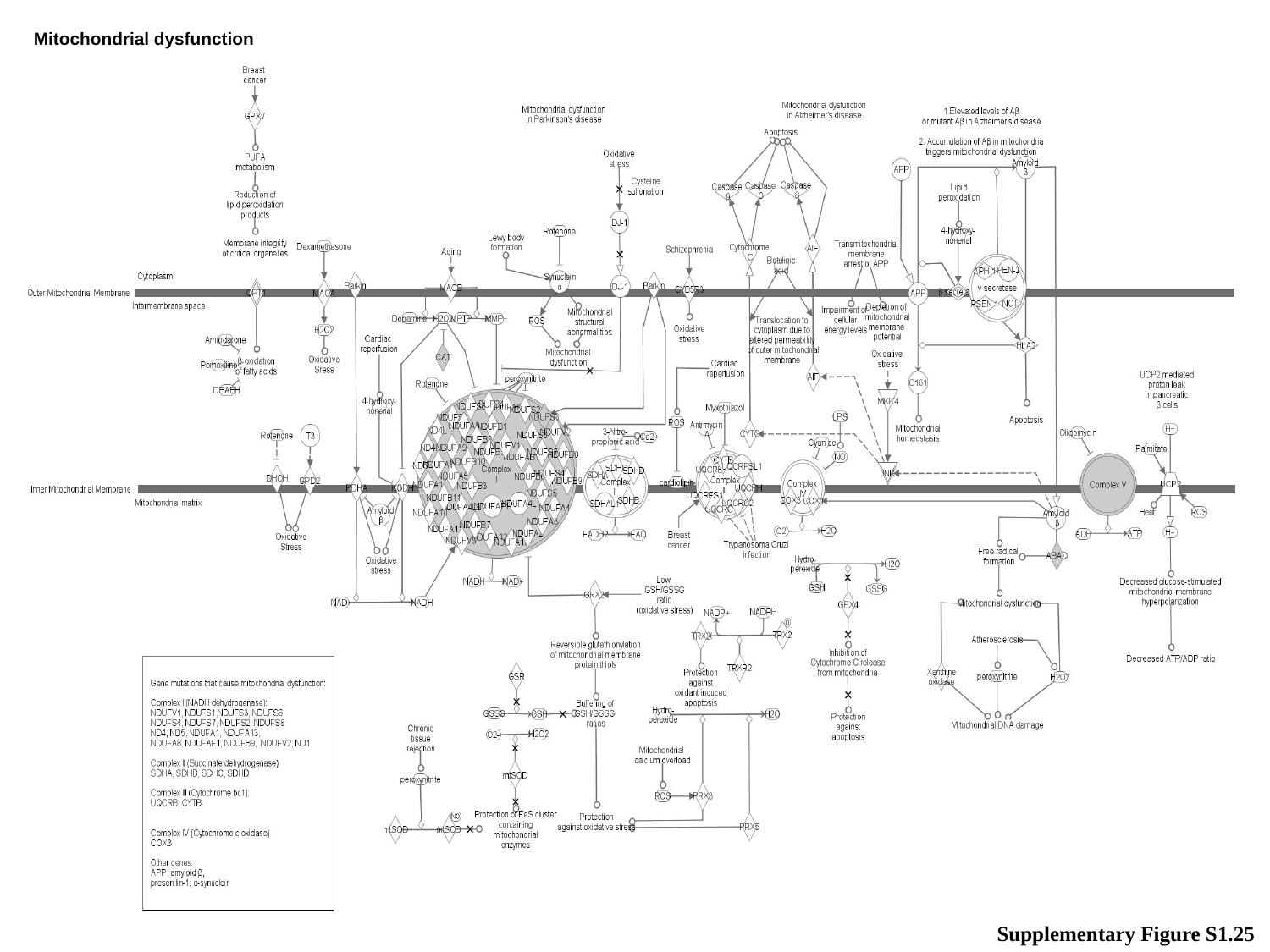

Mitochondrial dysfunction
Supplementary Figure S1.25

## Slide 28
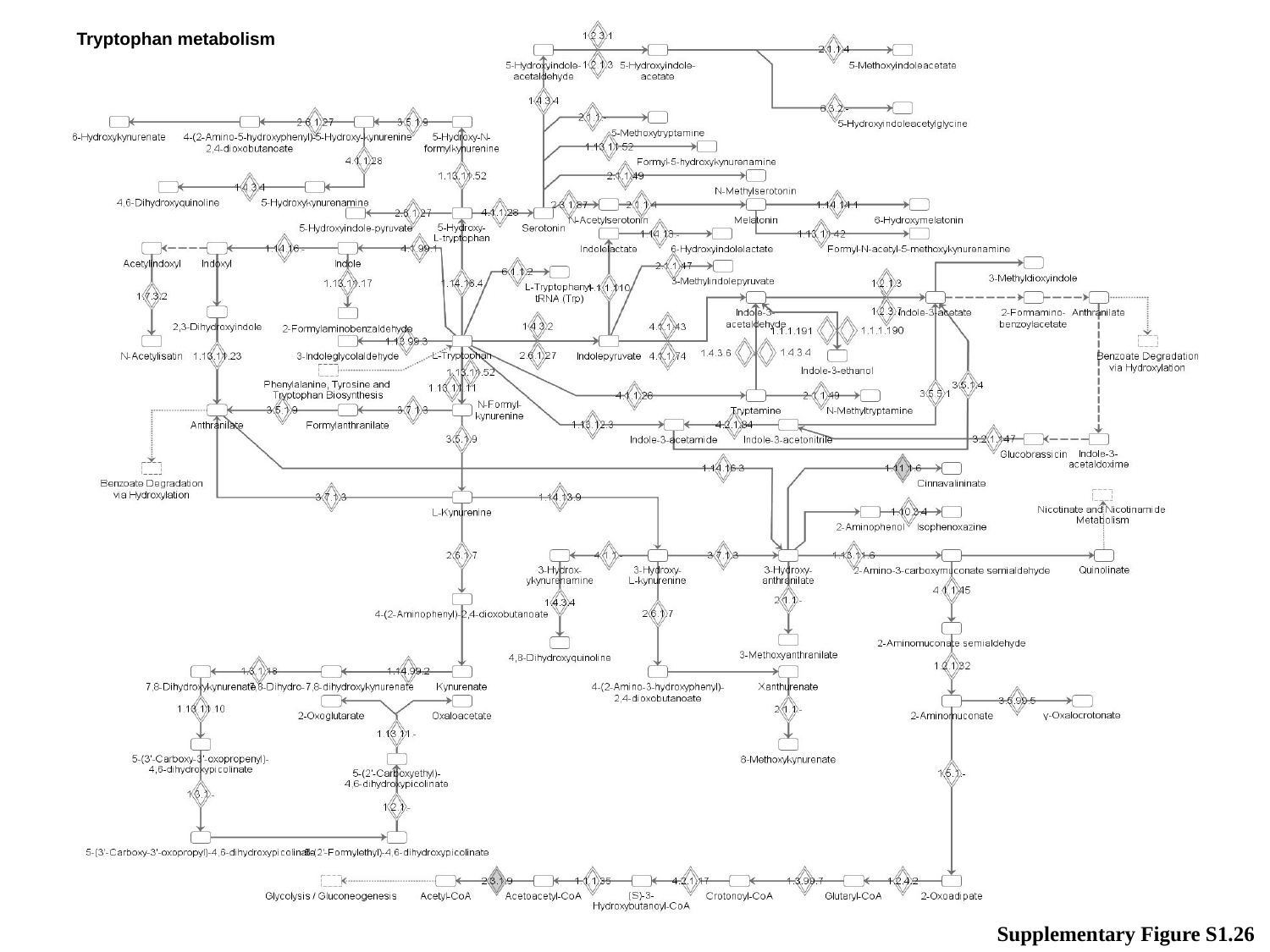

Tryptophan metabolism
Supplementary Figure S1.26

## Slide 29
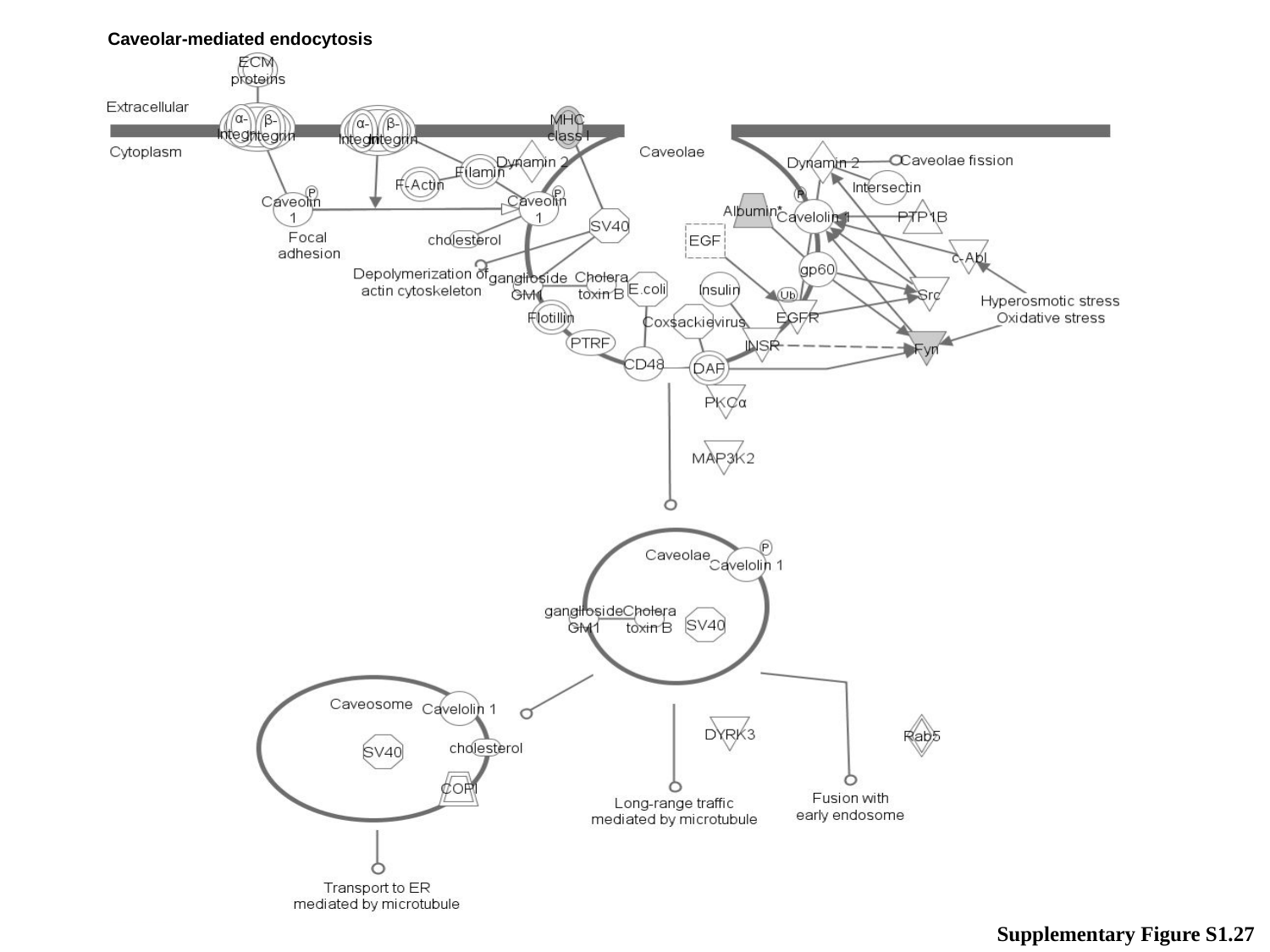

Caveolar-mediated endocytosis
Supplementary Figure S1.27

## Slide 30
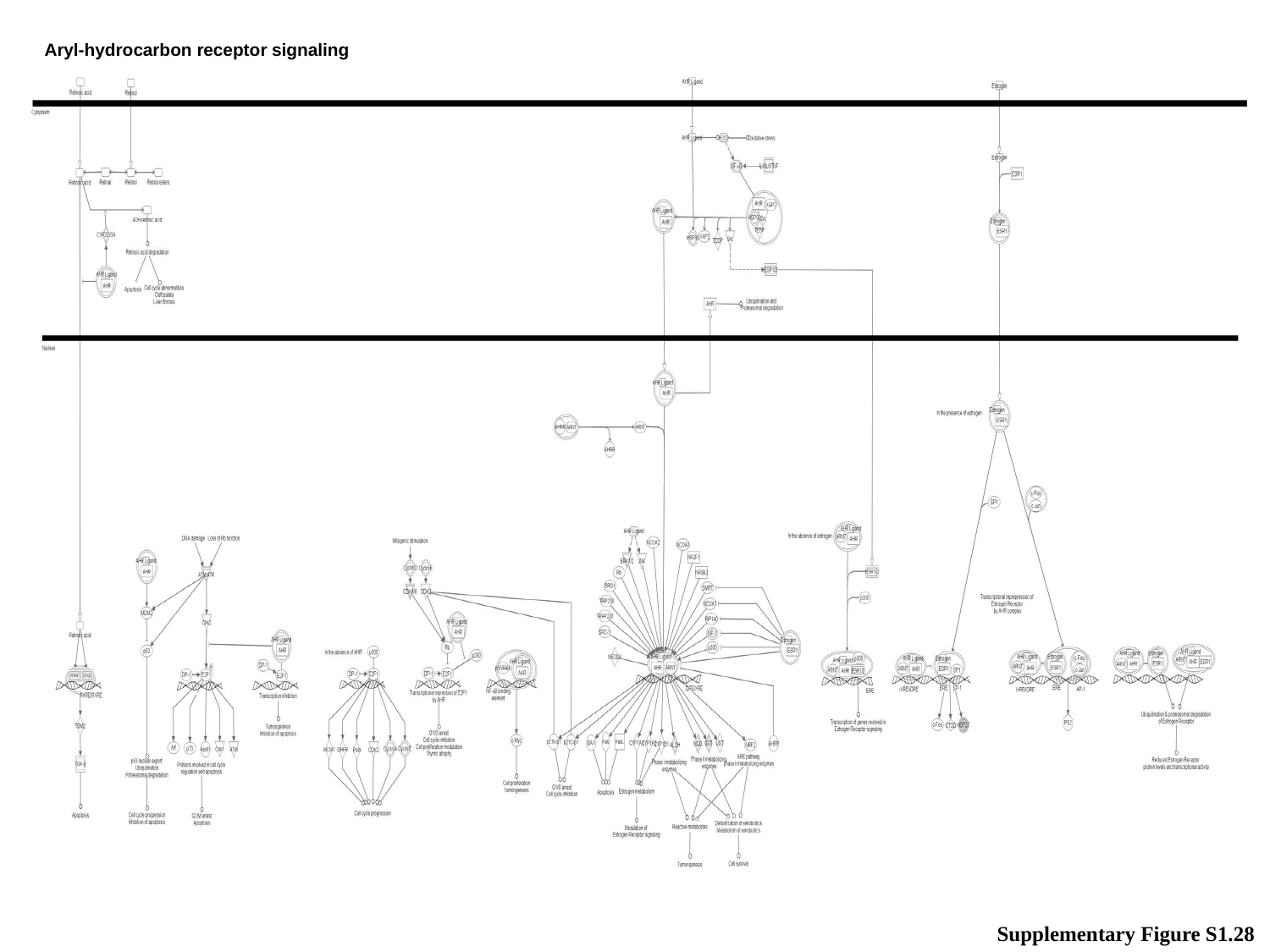

Aryl-hydrocarbon receptor signaling
Supplementary Figure S1.28

## Slide 31
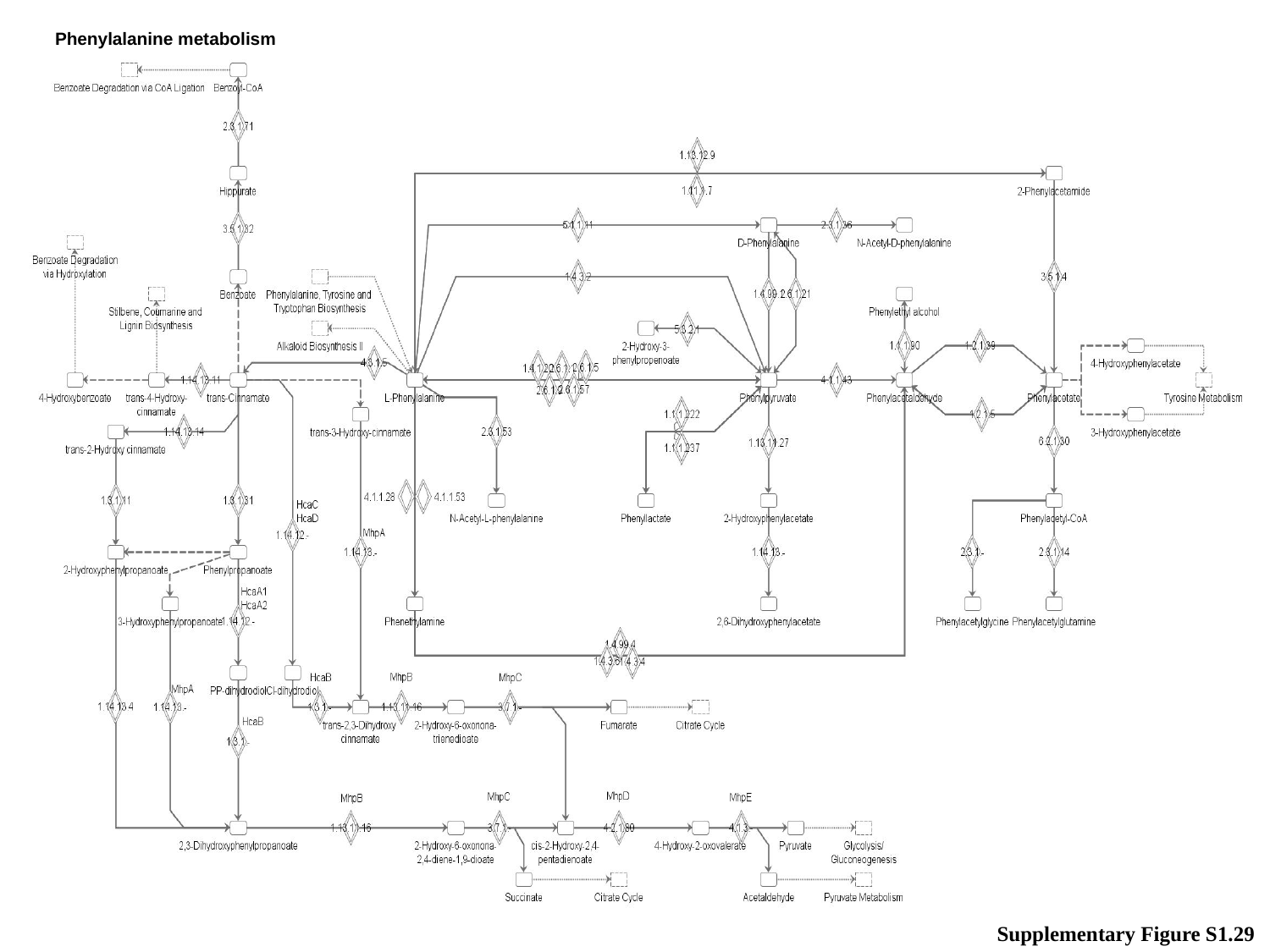

Phenylalanine metabolism
Supplementary Figure S1.29

## Slide 32
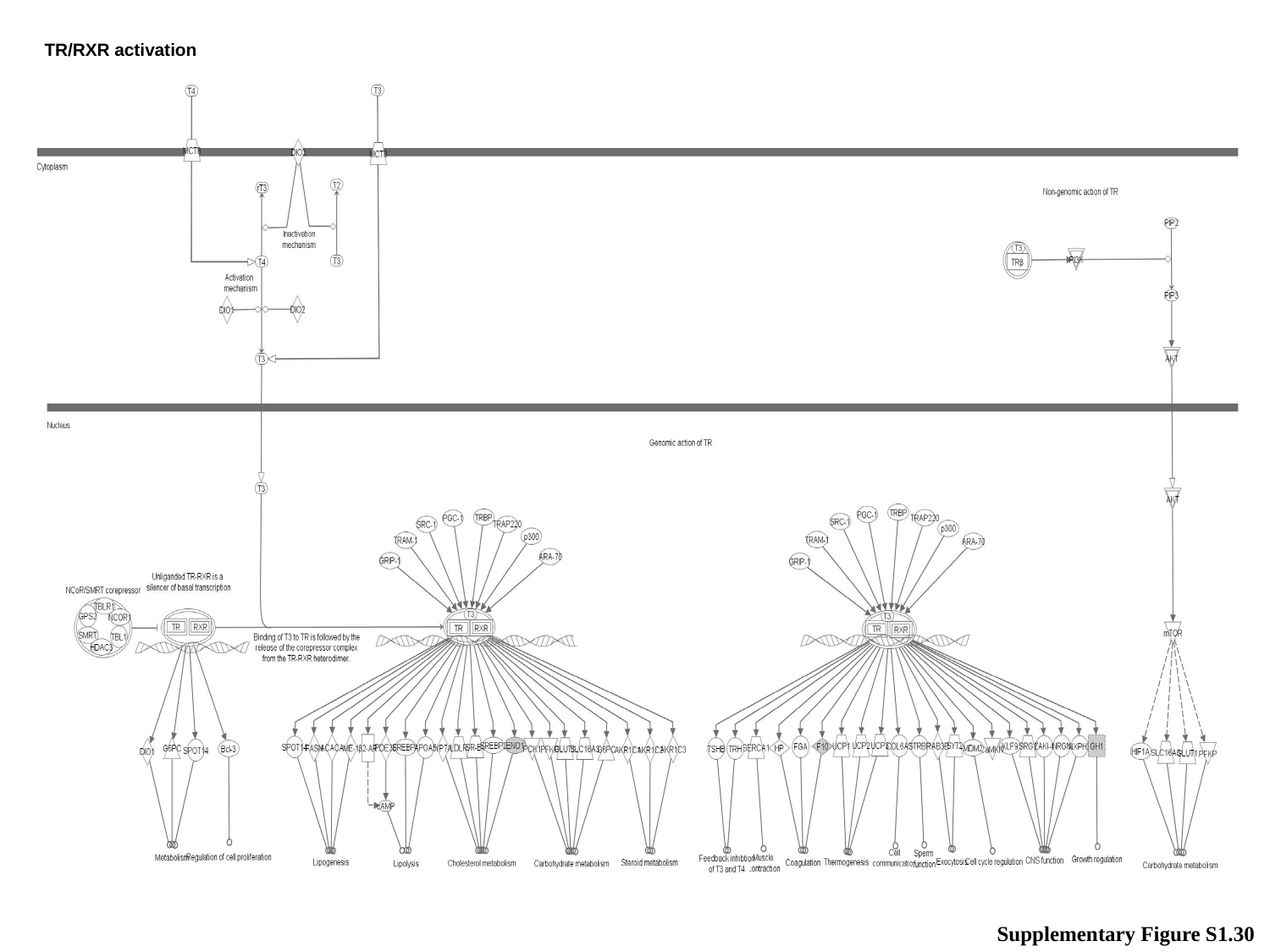

TR/RXR activation
Supplementary Figure S1.30

## Slide 33
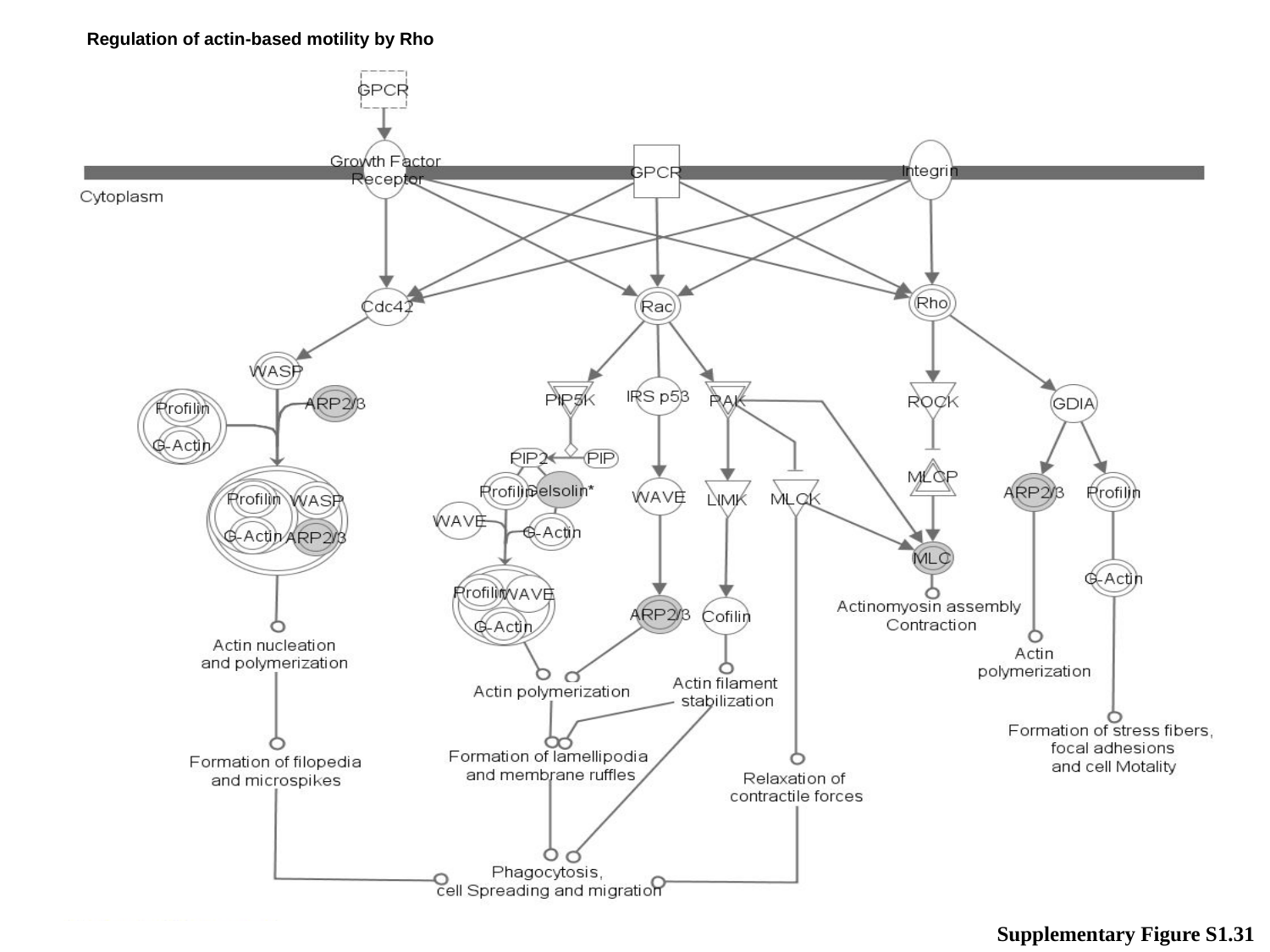

Regulation of actin-based motility by Rho
Supplementary Figure S1.31

## Slide 34
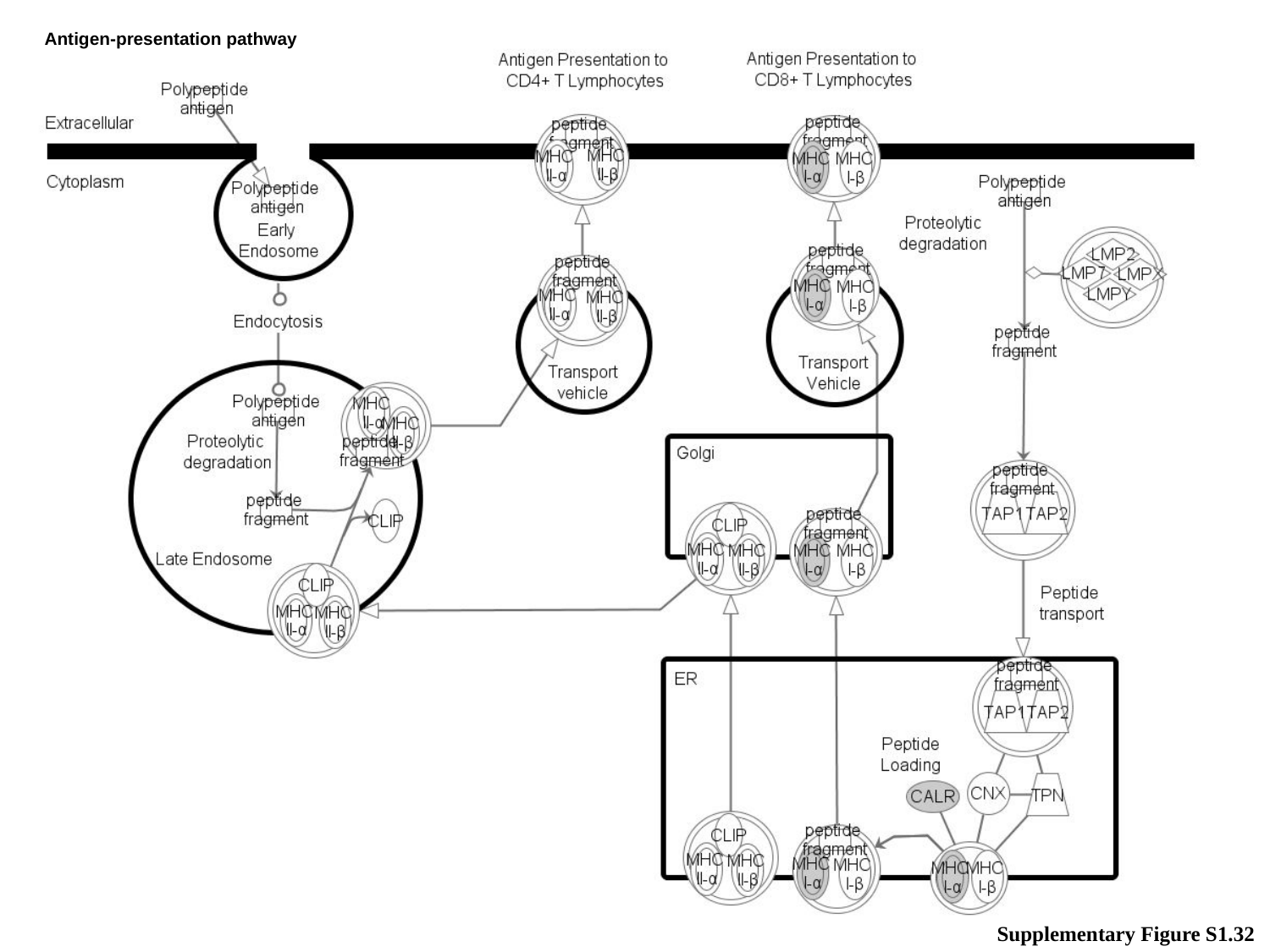

Antigen-presentation pathway
Supplementary Figure S1.32

## Slide 35
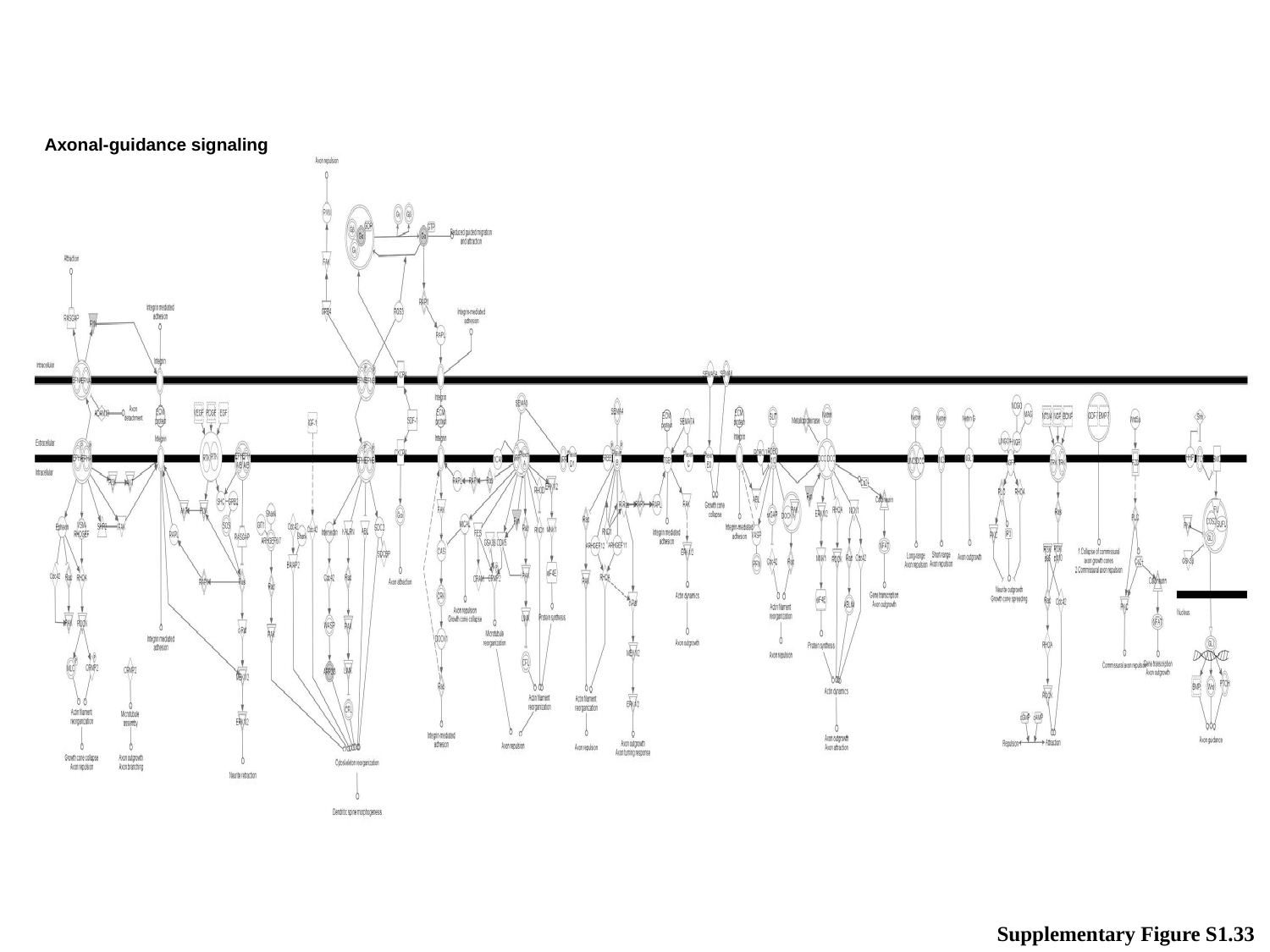

Axonal-guidance signaling
Supplementary Figure S1.33

## Slide 36
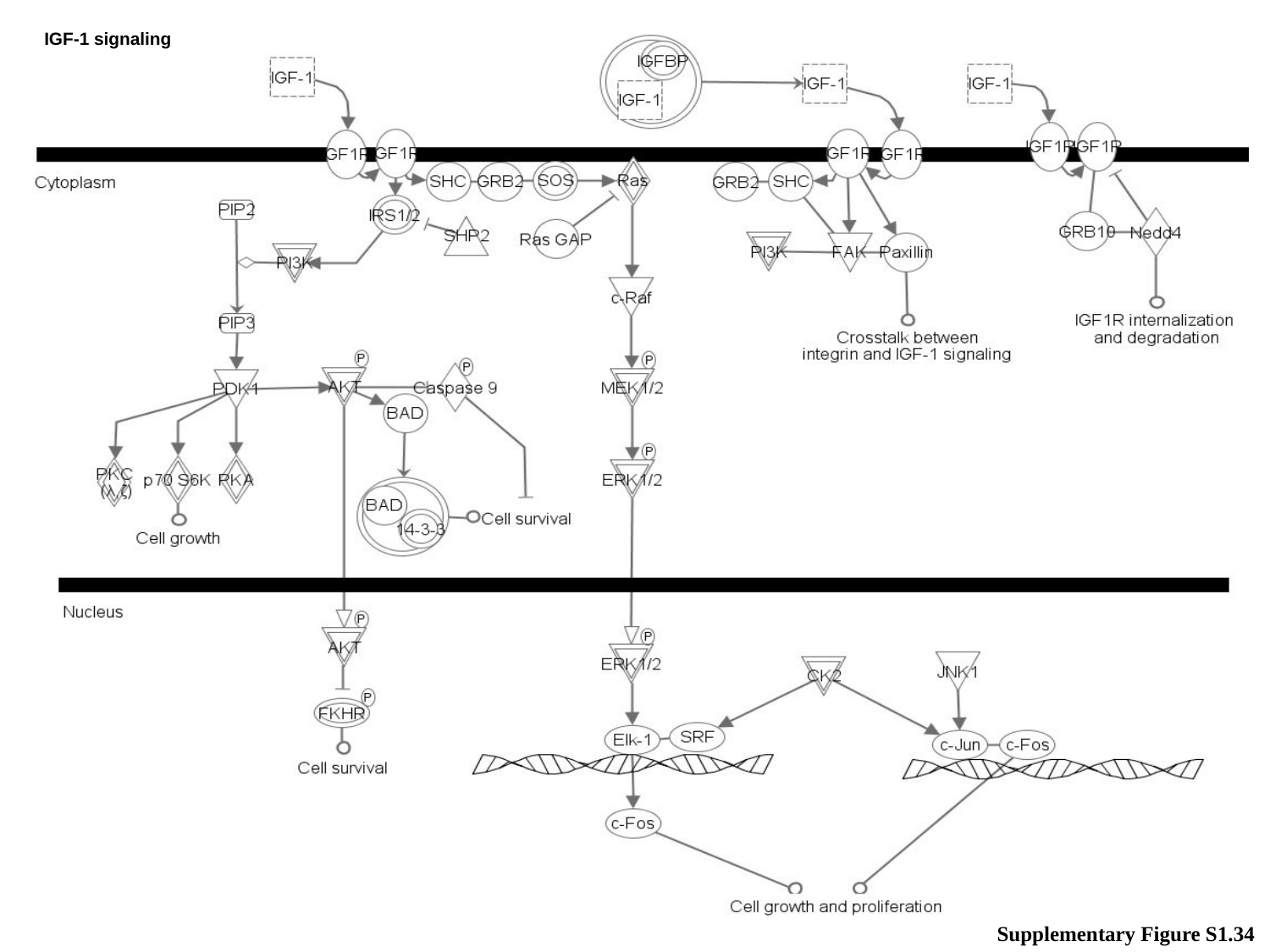

IGF-1 signaling
Supplementary Figure S1.34

## Slide 37
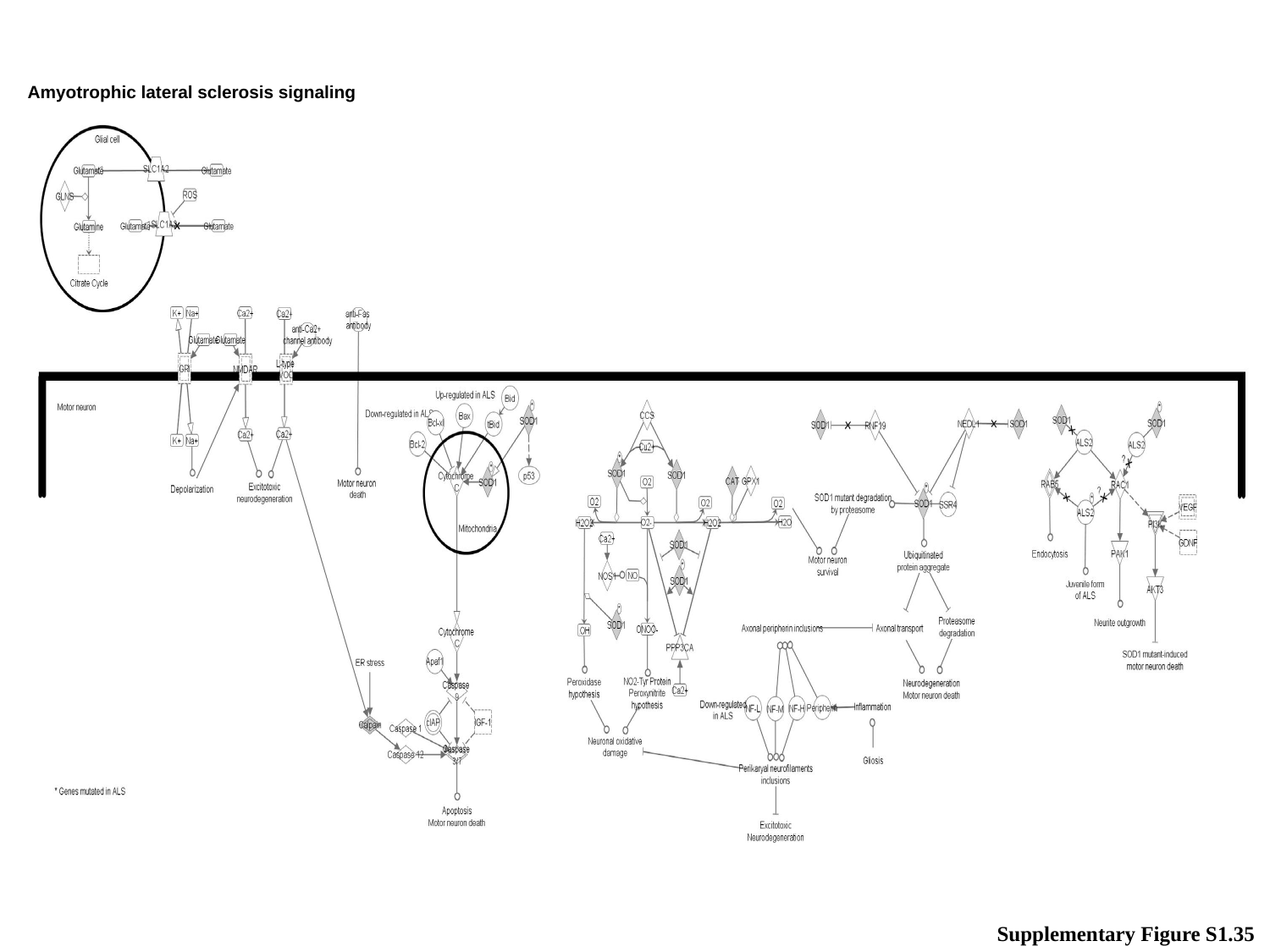

Amyotrophic lateral sclerosis signaling
Supplementary Figure S1.35

## Slide 38
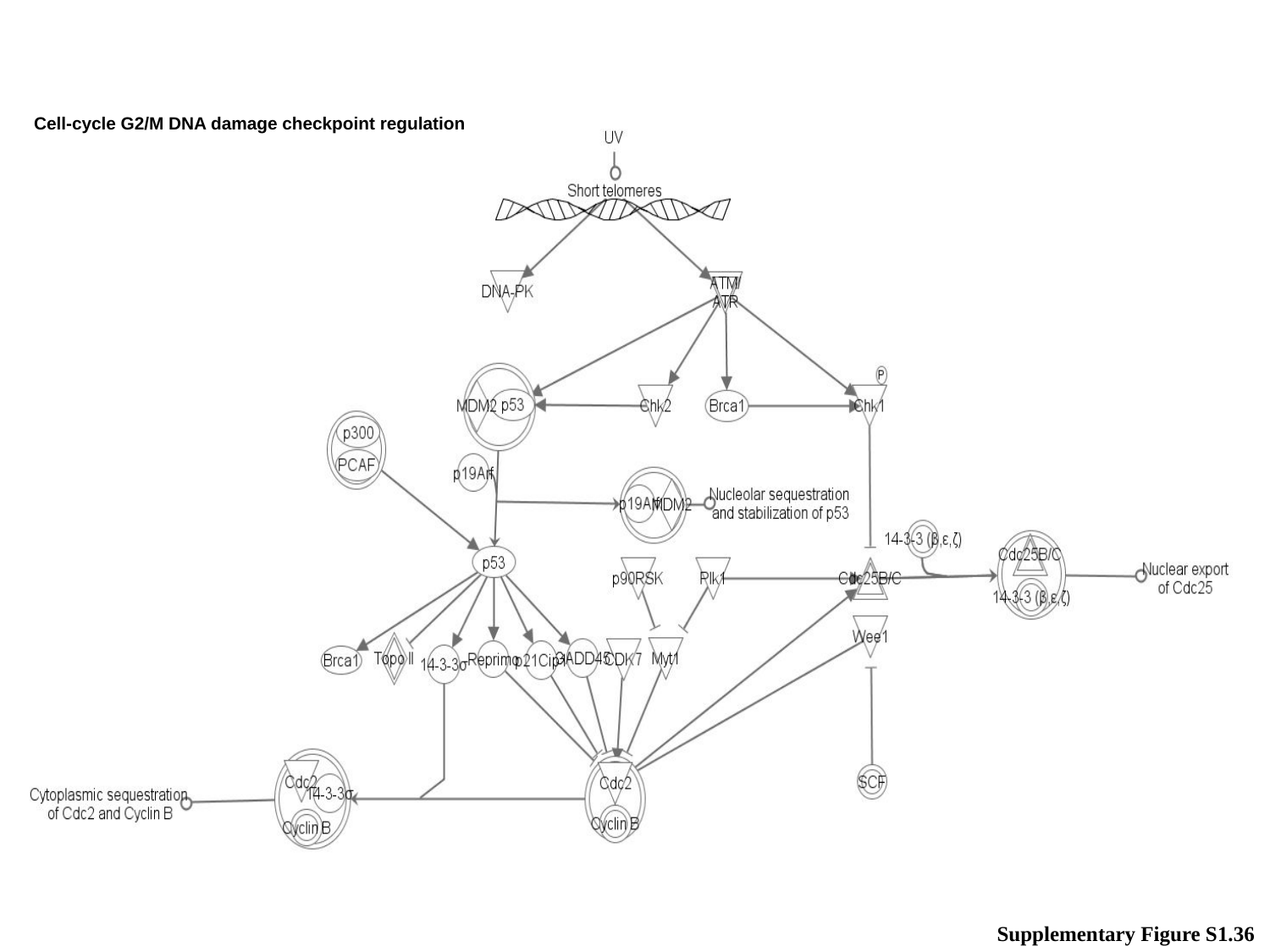

Cell-cycle G2/M DNA damage checkpoint regulation
Supplementary Figure S1.36

## Slide 39
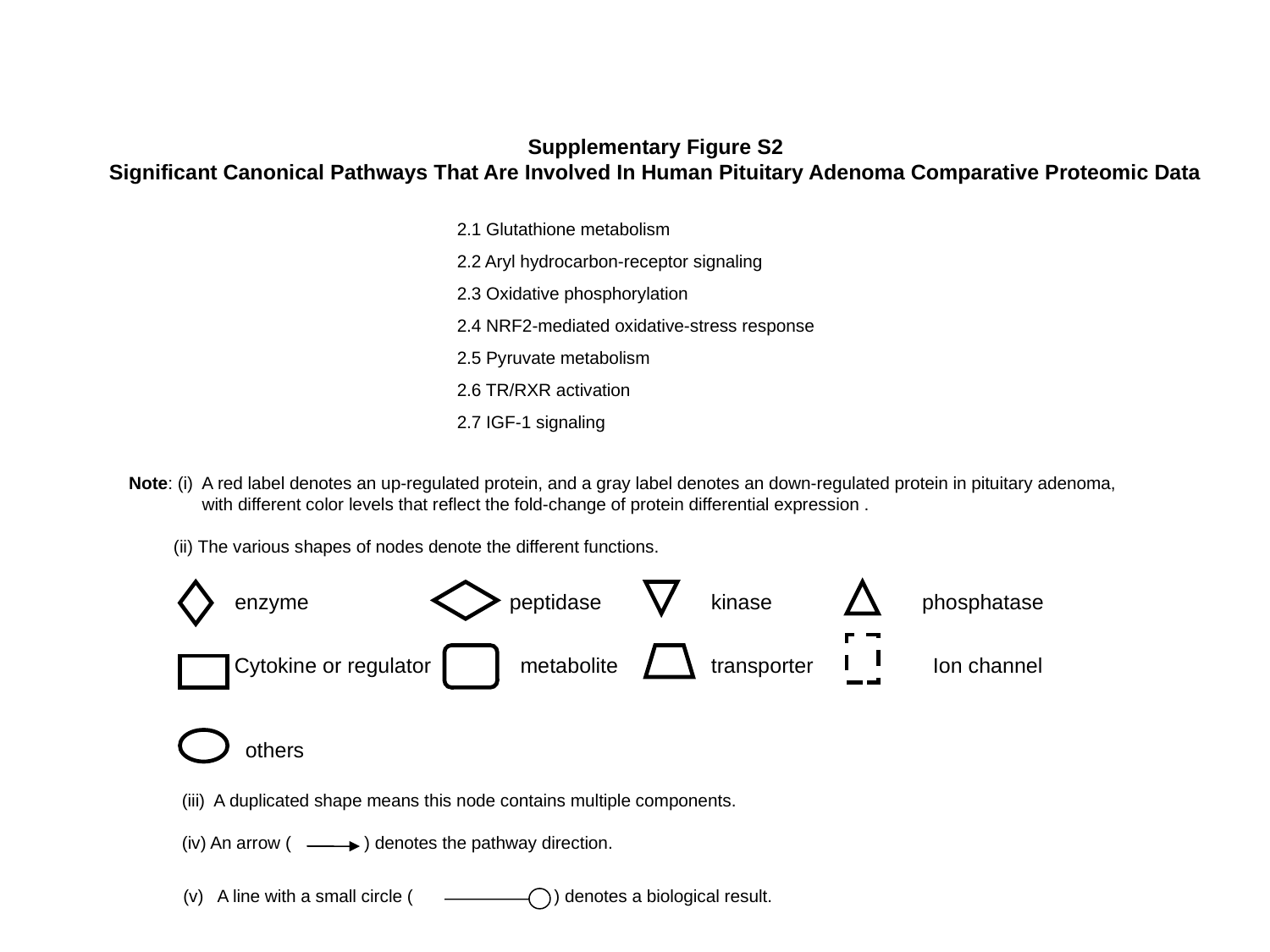

Supplementary Figure S2
Significant Canonical Pathways That Are Involved In Human Pituitary Adenoma Comparative Proteomic Data
2.1 Glutathione metabolism
2.2 Aryl hydrocarbon-receptor signaling
2.3 Oxidative phosphorylation
2.4 NRF2-mediated oxidative-stress response
2.5 Pyruvate metabolism
2.6 TR/RXR activation
2.7 IGF-1 signaling
Note: (i) A red label denotes an up-regulated protein, and a gray label denotes an down-regulated protein in pituitary adenoma,
 with different color levels that reflect the fold-change of protein differential expression .
 (ii) The various shapes of nodes denote the different functions.
enzyme
peptidase
kinase
phosphatase
Cytokine or regulator
metabolite
transporter
Ion channel
others
(iii) A duplicated shape means this node contains multiple components.
(v) A line with a small circle ( ) denotes a biological result.
(iv) An arrow ( ) denotes the pathway direction.

## Slide 40
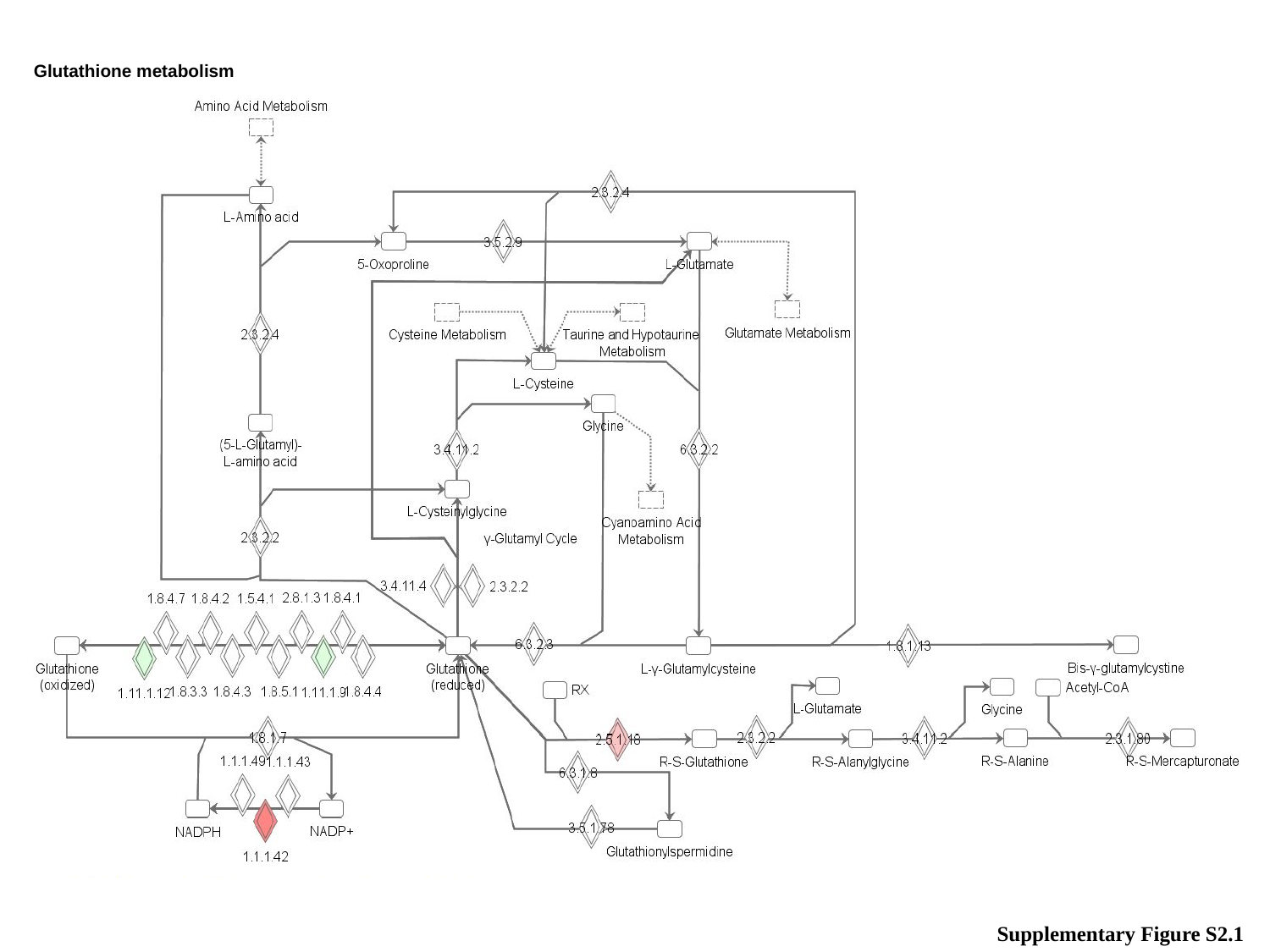

Glutathione metabolism
Supplementary Figure S2.1

## Slide 41
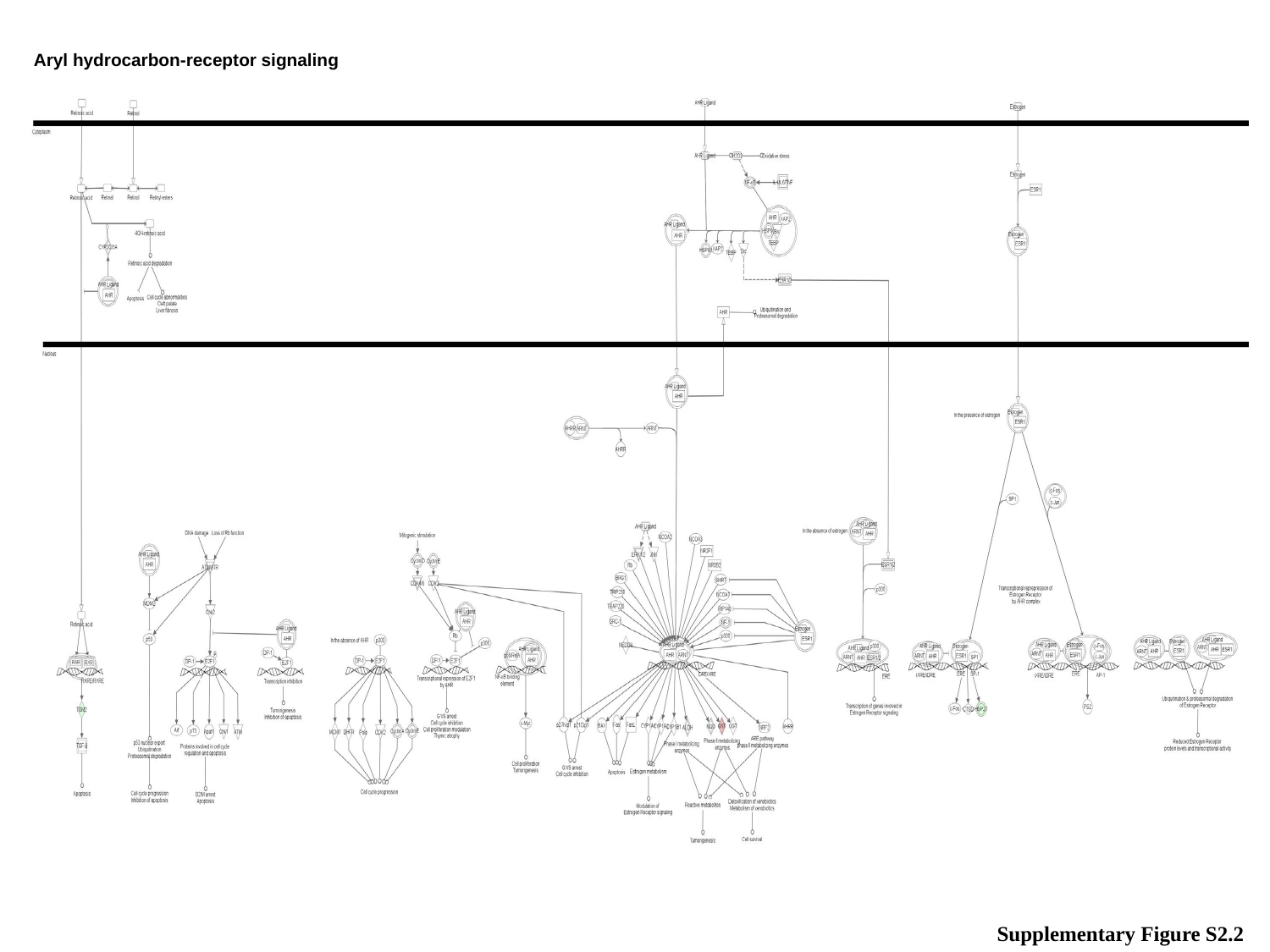

Aryl hydrocarbon-receptor signaling
Supplementary Figure S2.2

## Slide 42
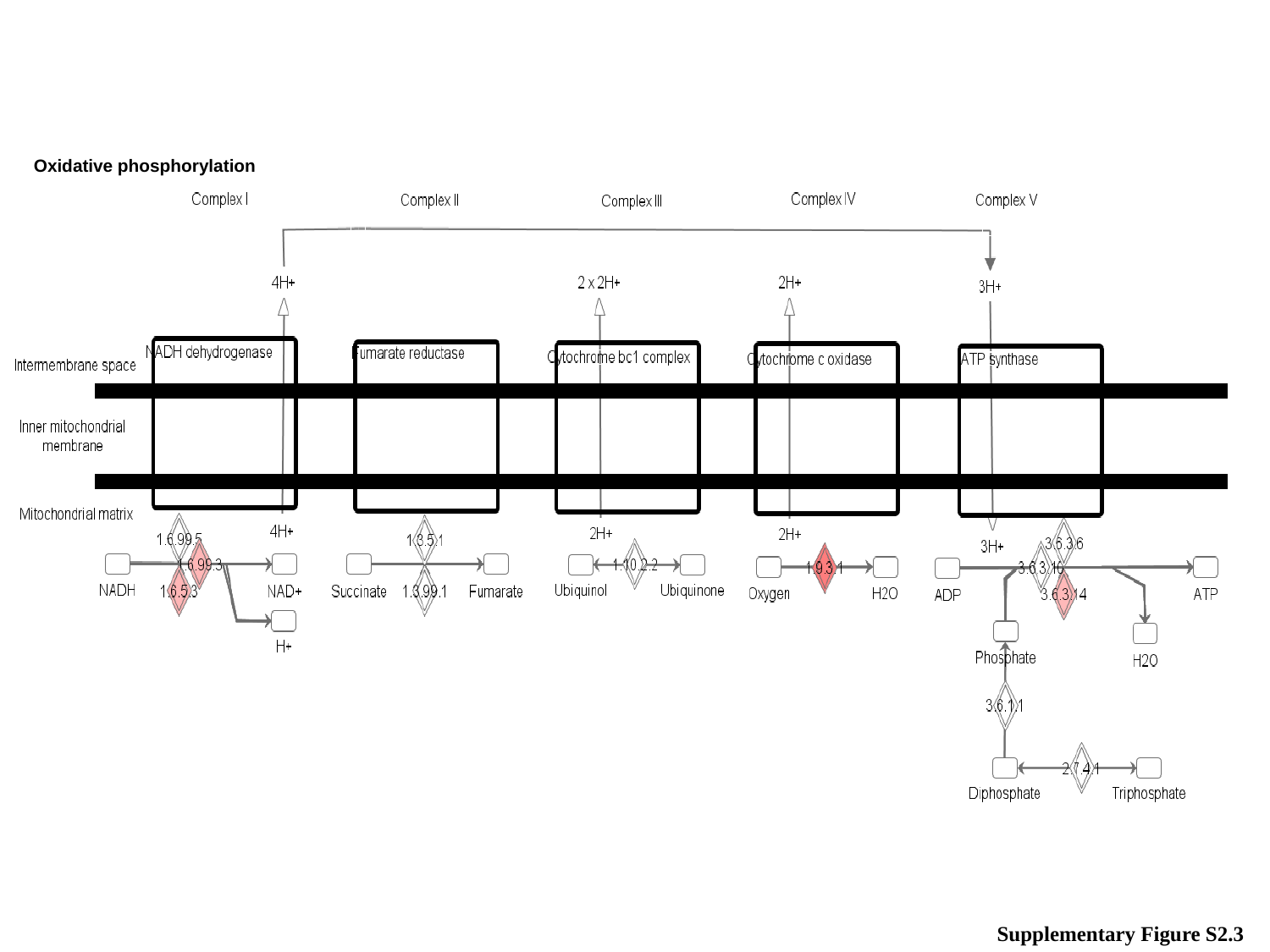

Oxidative phosphorylation
Supplementary Figure S2.3

## Slide 43
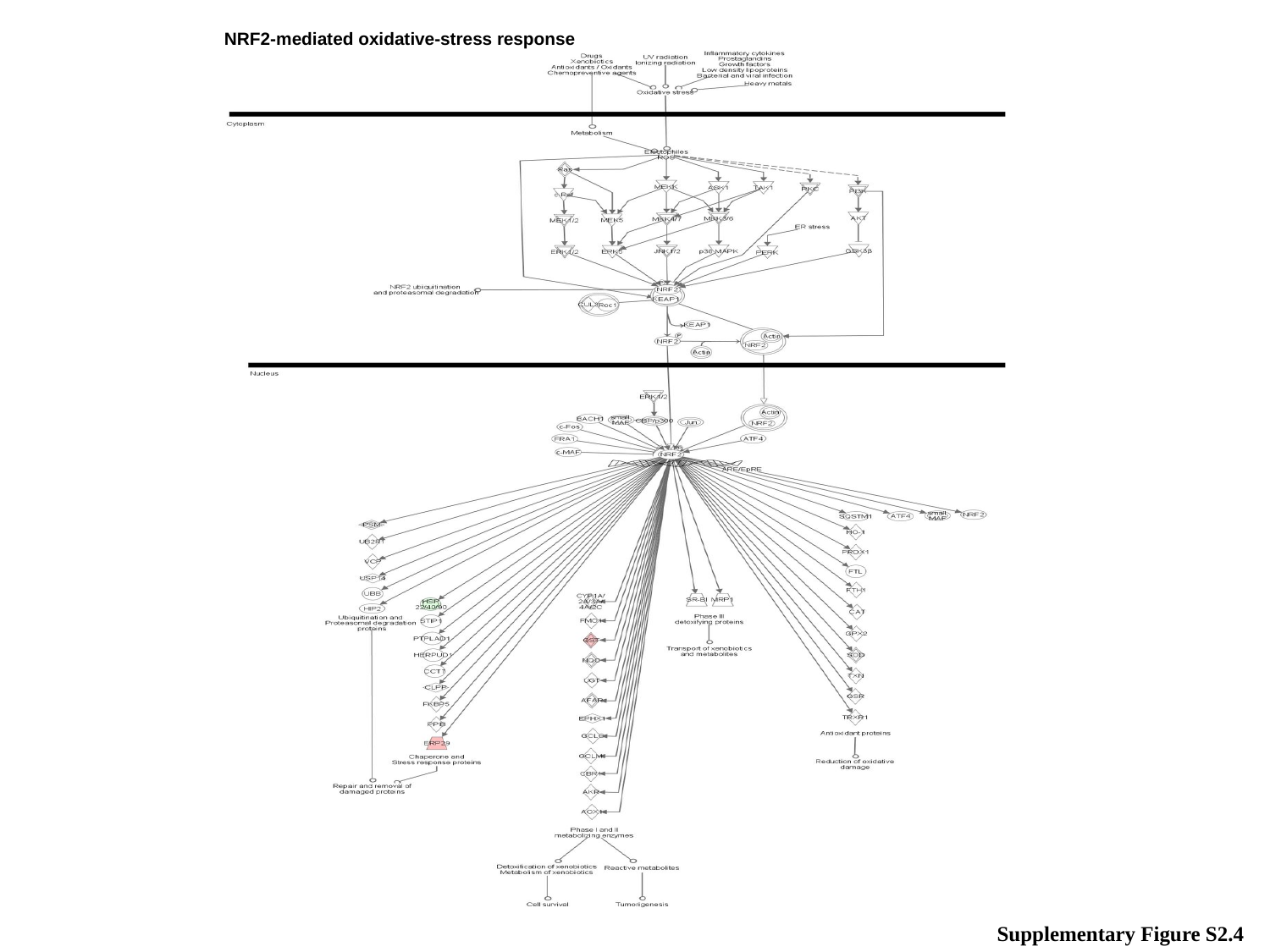

NRF2-mediated oxidative-stress response
Supplementary Figure S2.4

## Slide 44
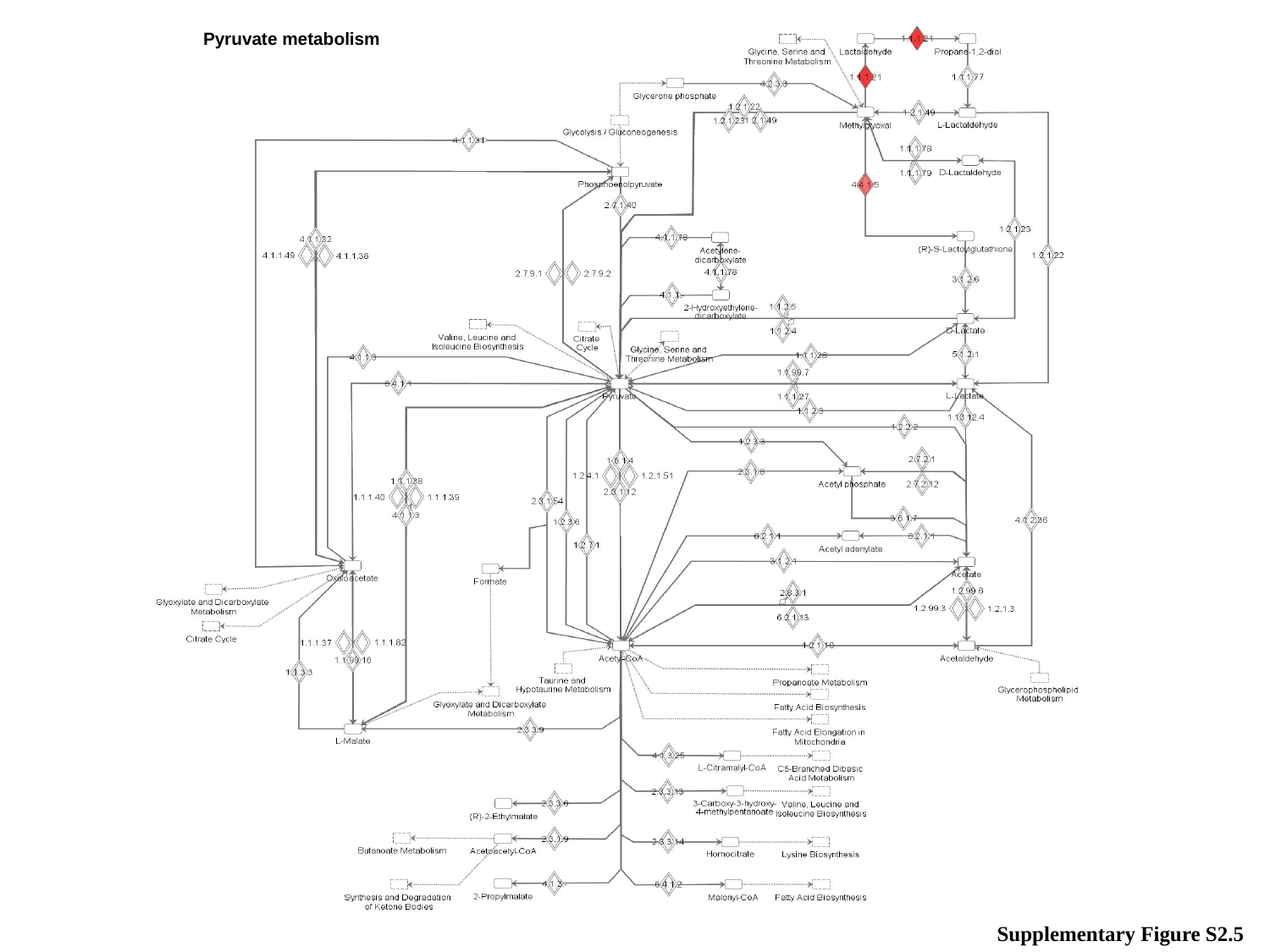

Pyruvate metabolism
Supplementary Figure S2.5

## Slide 45
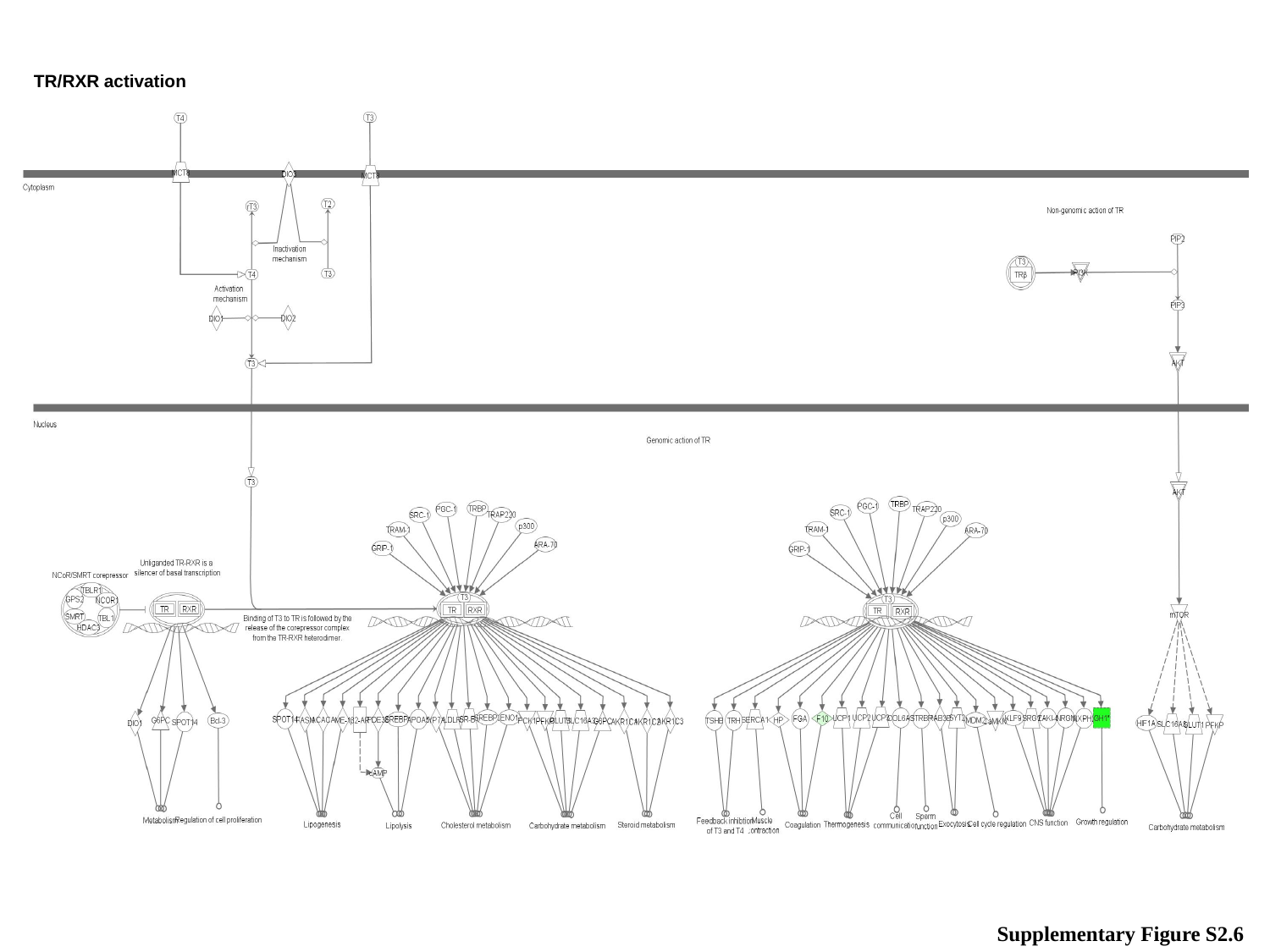

TR/RXR activation
Supplementary Figure S2.6

## Slide 46
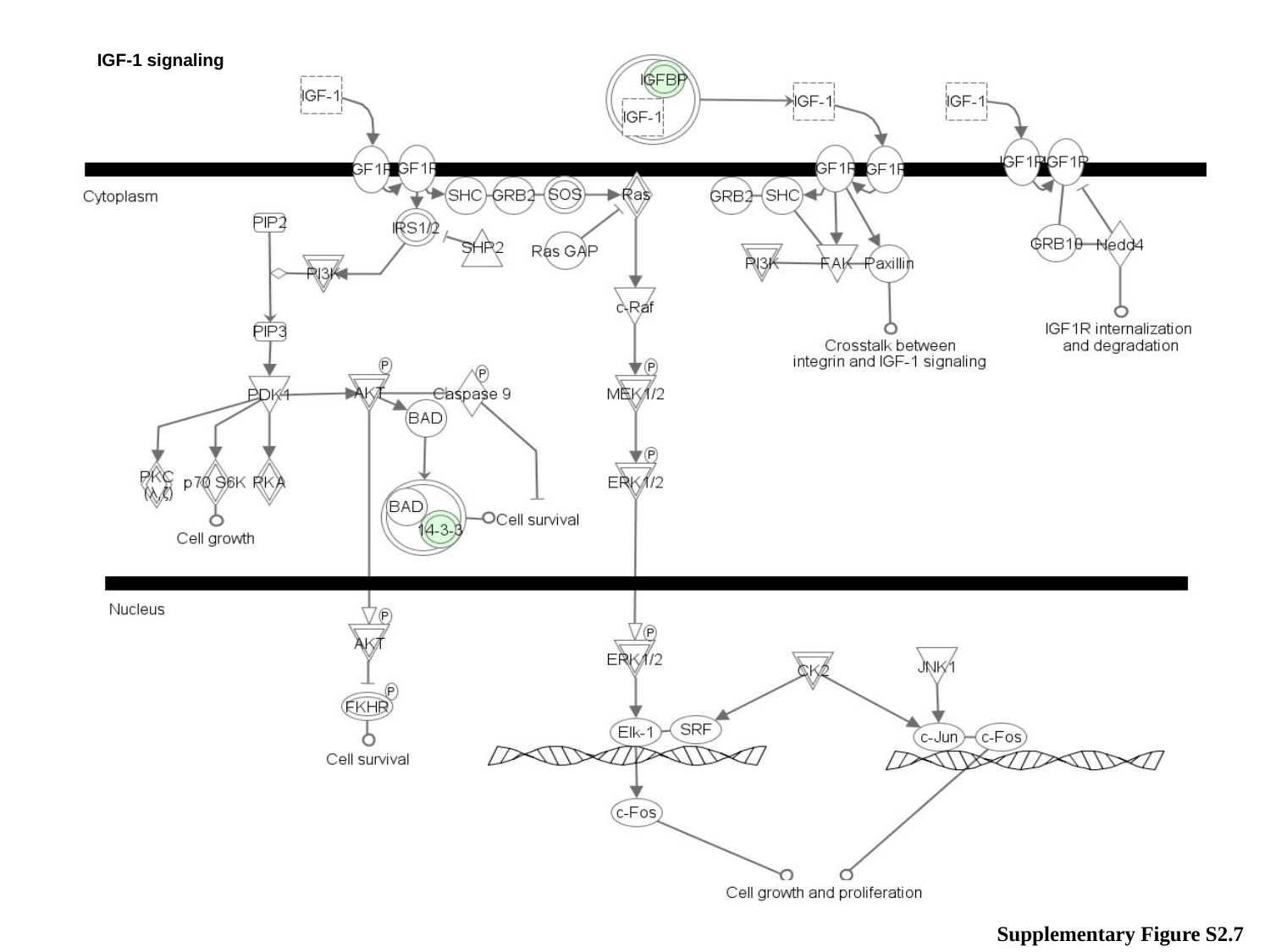

IGF-1 signaling
Supplementary Figure S2.7

## Slide 47
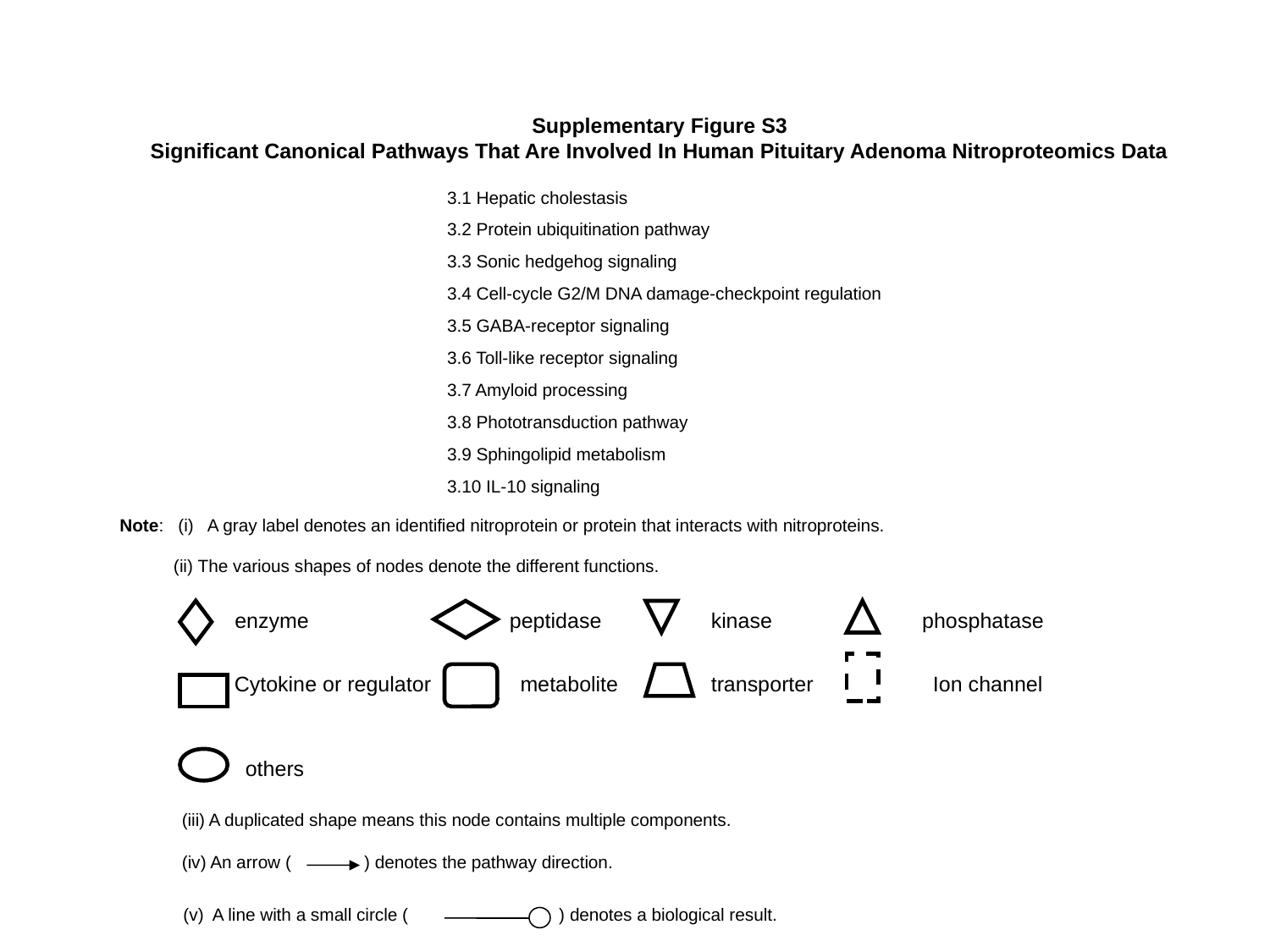

Supplementary Figure S3
Significant Canonical Pathways That Are Involved In Human Pituitary Adenoma Nitroproteomics Data
3.1 Hepatic cholestasis
3.2 Protein ubiquitination pathway
3.3 Sonic hedgehog signaling
3.4 Cell-cycle G2/M DNA damage-checkpoint regulation
3.5 GABA-receptor signaling
3.6 Toll-like receptor signaling
3.7 Amyloid processing
3.8 Phototransduction pathway
3.9 Sphingolipid metabolism
3.10 IL-10 signaling
Note: (i) A gray label denotes an identified nitroprotein or protein that interacts with nitroproteins.
 (ii) The various shapes of nodes denote the different functions.
enzyme
peptidase
kinase
phosphatase
Cytokine or regulator
metabolite
transporter
Ion channel
others
(iii) A duplicated shape means this node contains multiple components.
(v) A line with a small circle ( ) denotes a biological result.
(iv) An arrow ( ) denotes the pathway direction.

## Slide 48
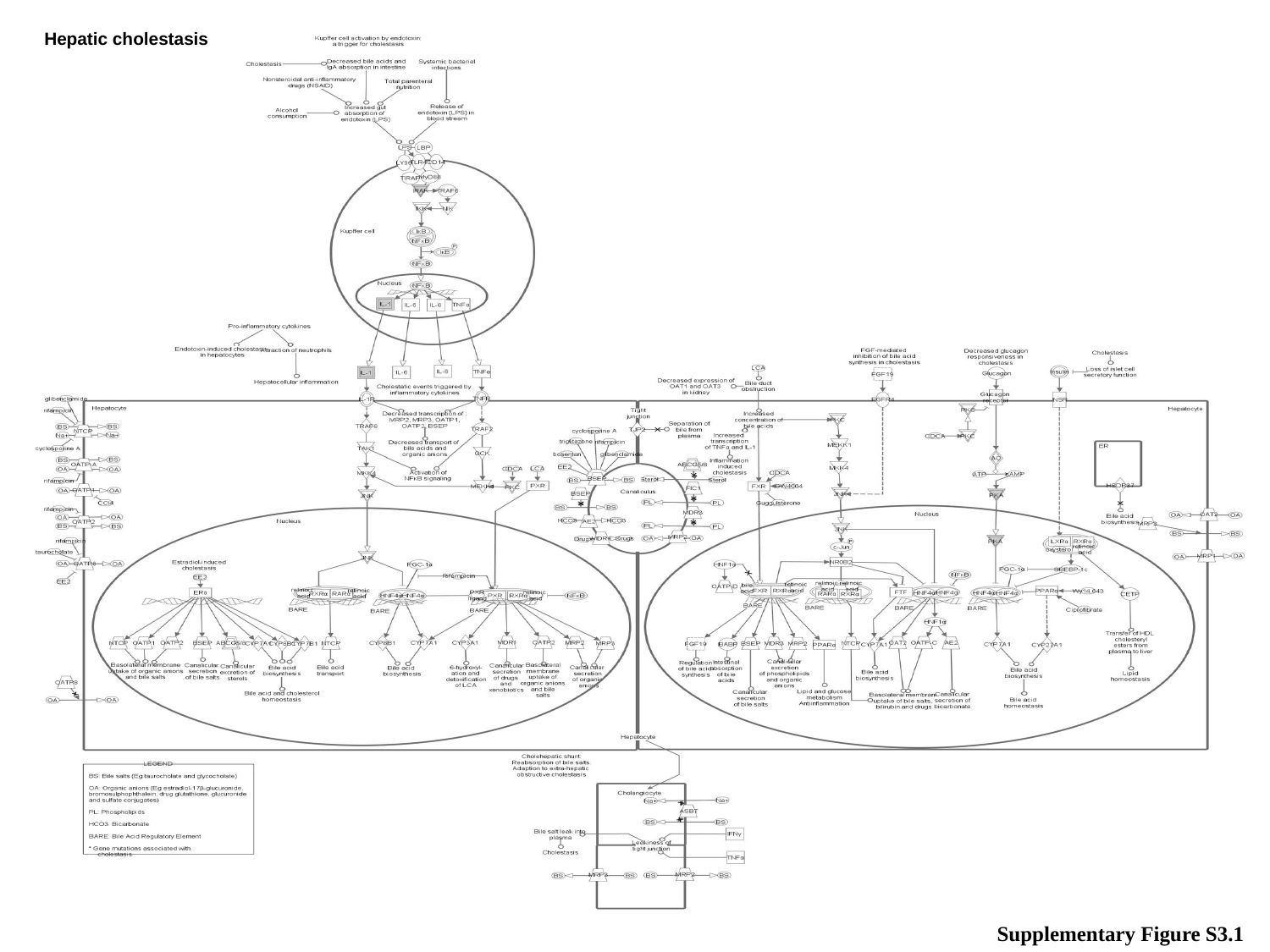

Hepatic cholestasis
Supplementary Figure S3.1

## Slide 49
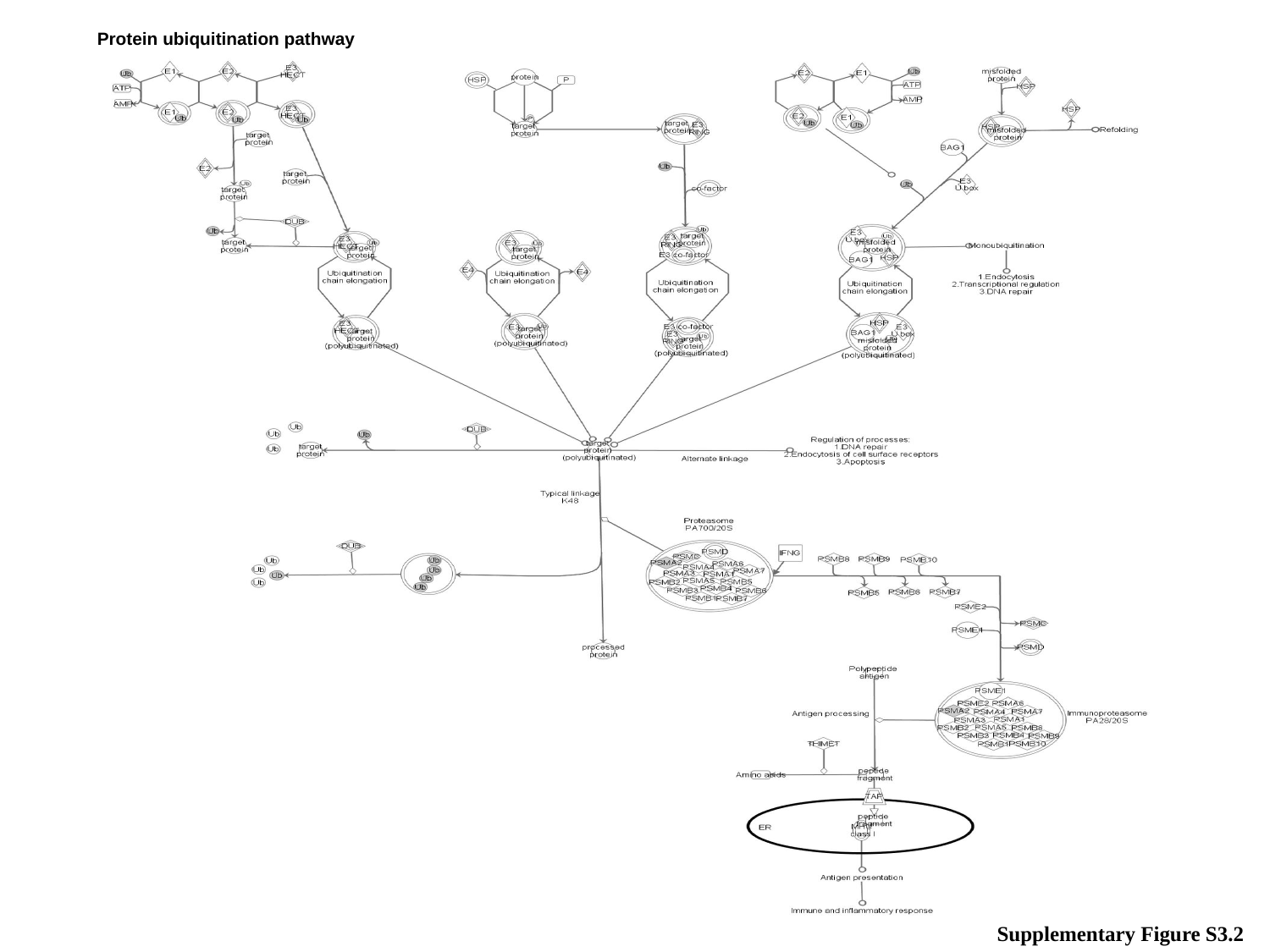

Protein ubiquitination pathway
Supplementary Figure S3.2

## Slide 50
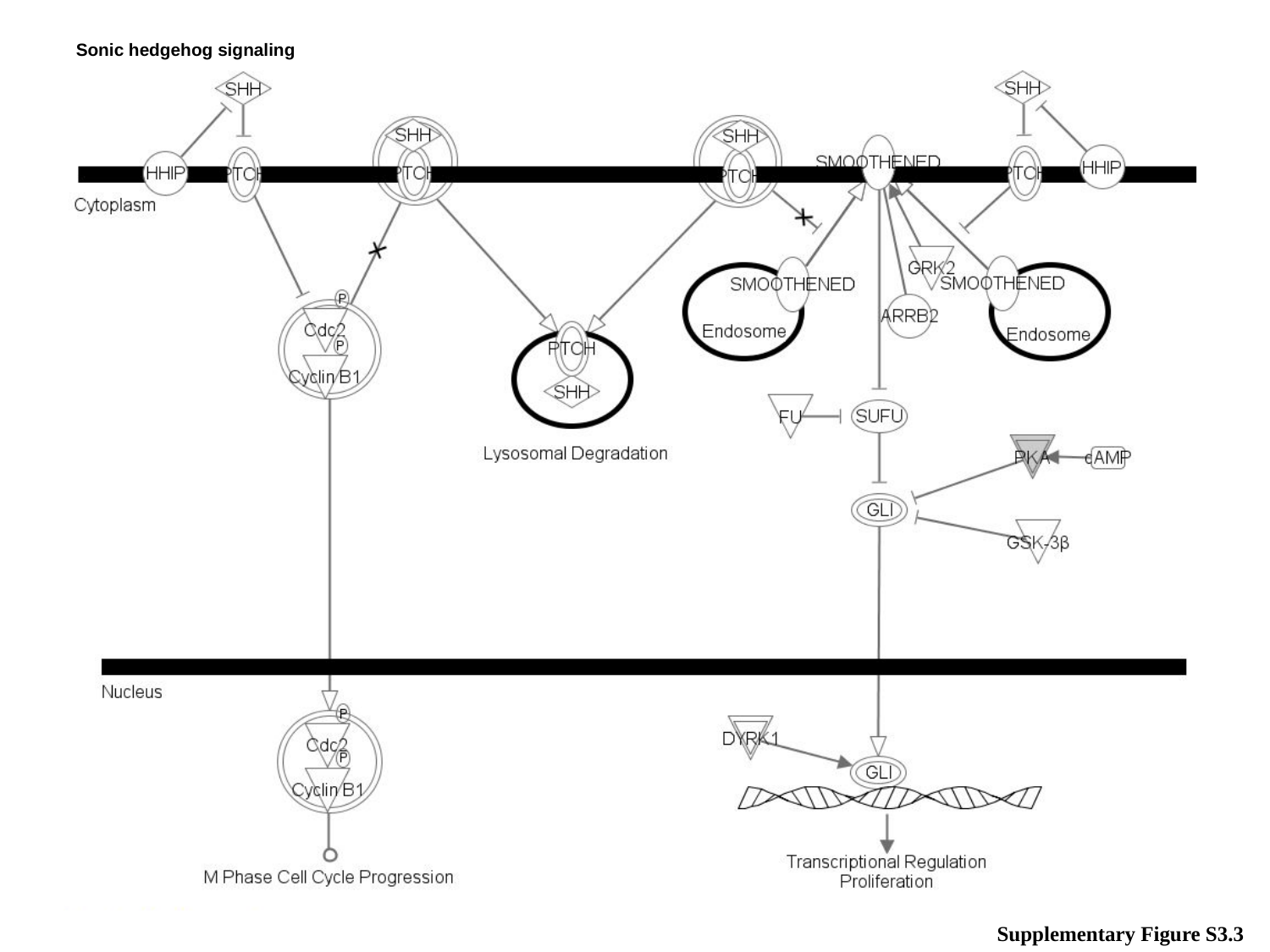

Sonic hedgehog signaling
Supplementary Figure S3.3

## Slide 51
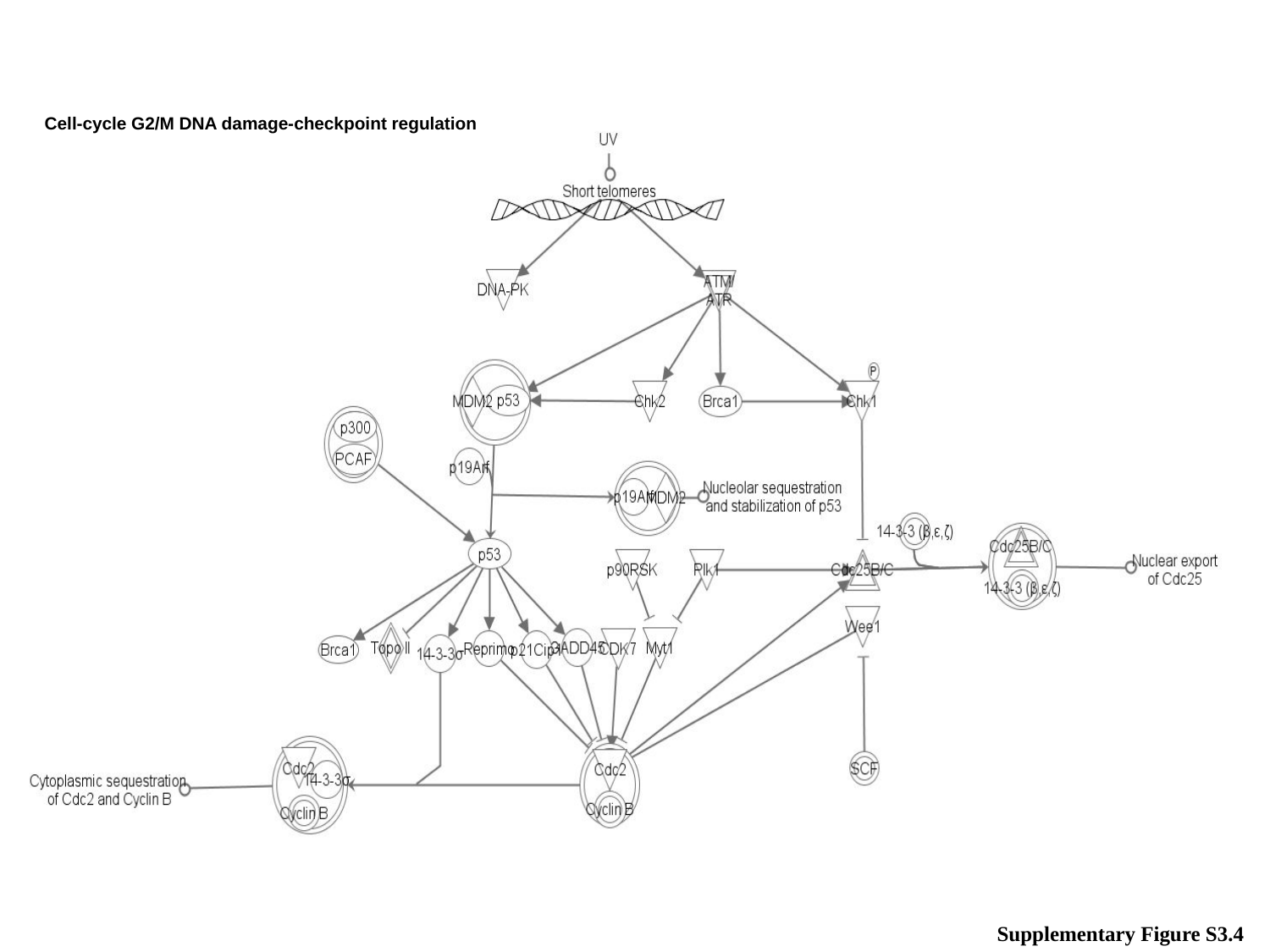

Cell-cycle G2/M DNA damage-checkpoint regulation
Supplementary Figure S3.4

## Slide 52
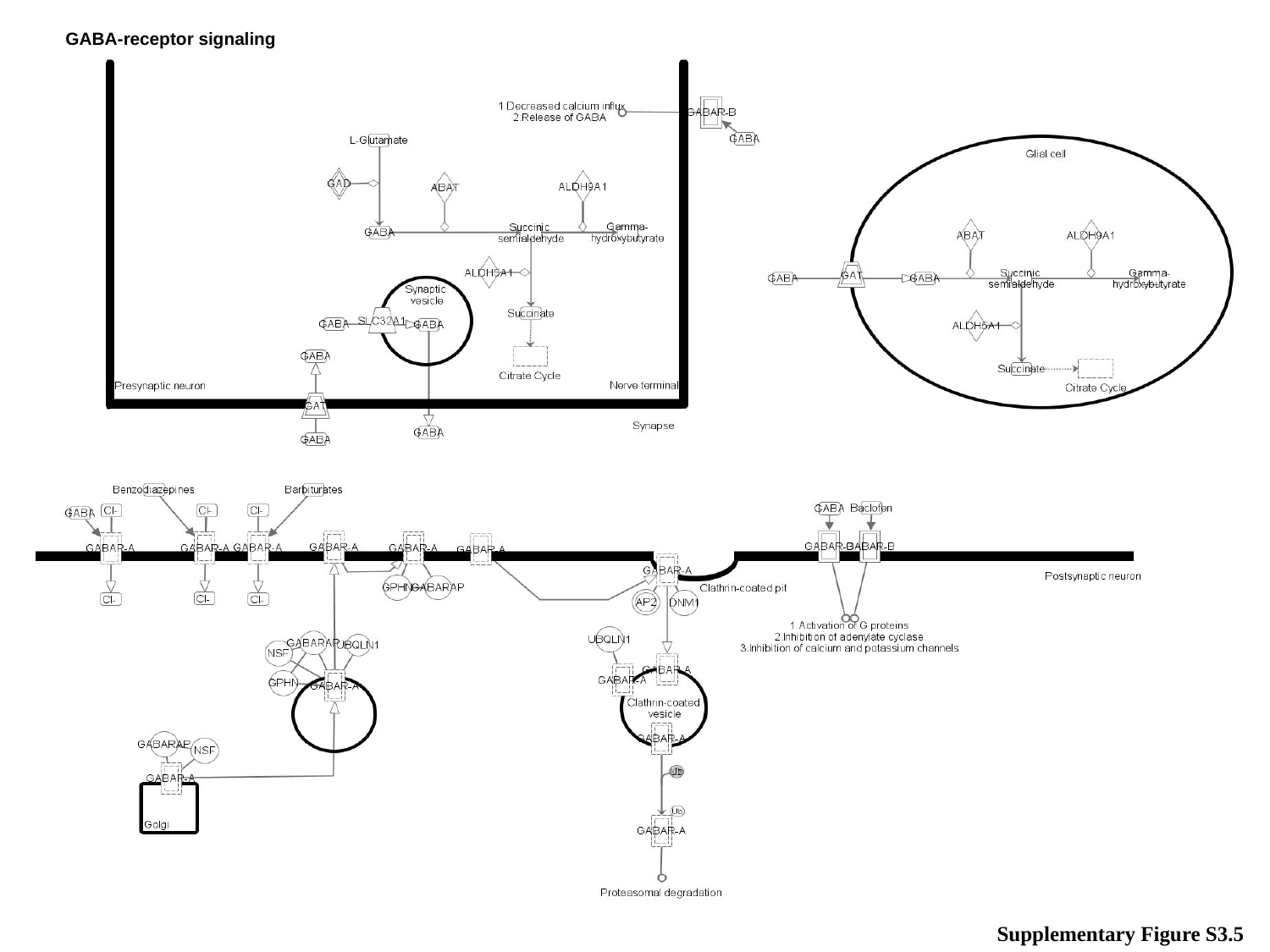

GABA-receptor signaling
Supplementary Figure S3.5

## Slide 53
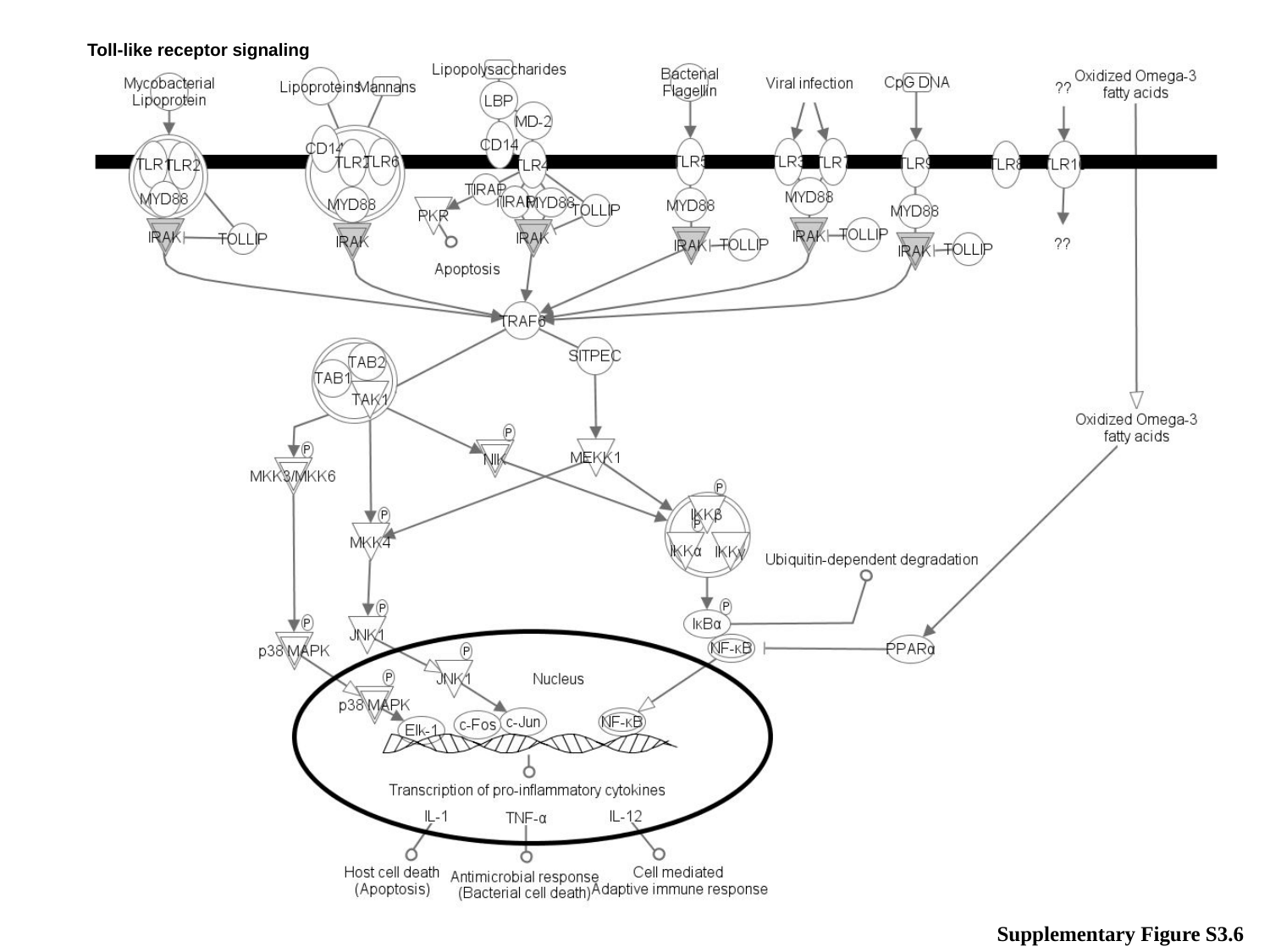

Toll-like receptor signaling
Supplementary Figure S3.6

## Slide 54
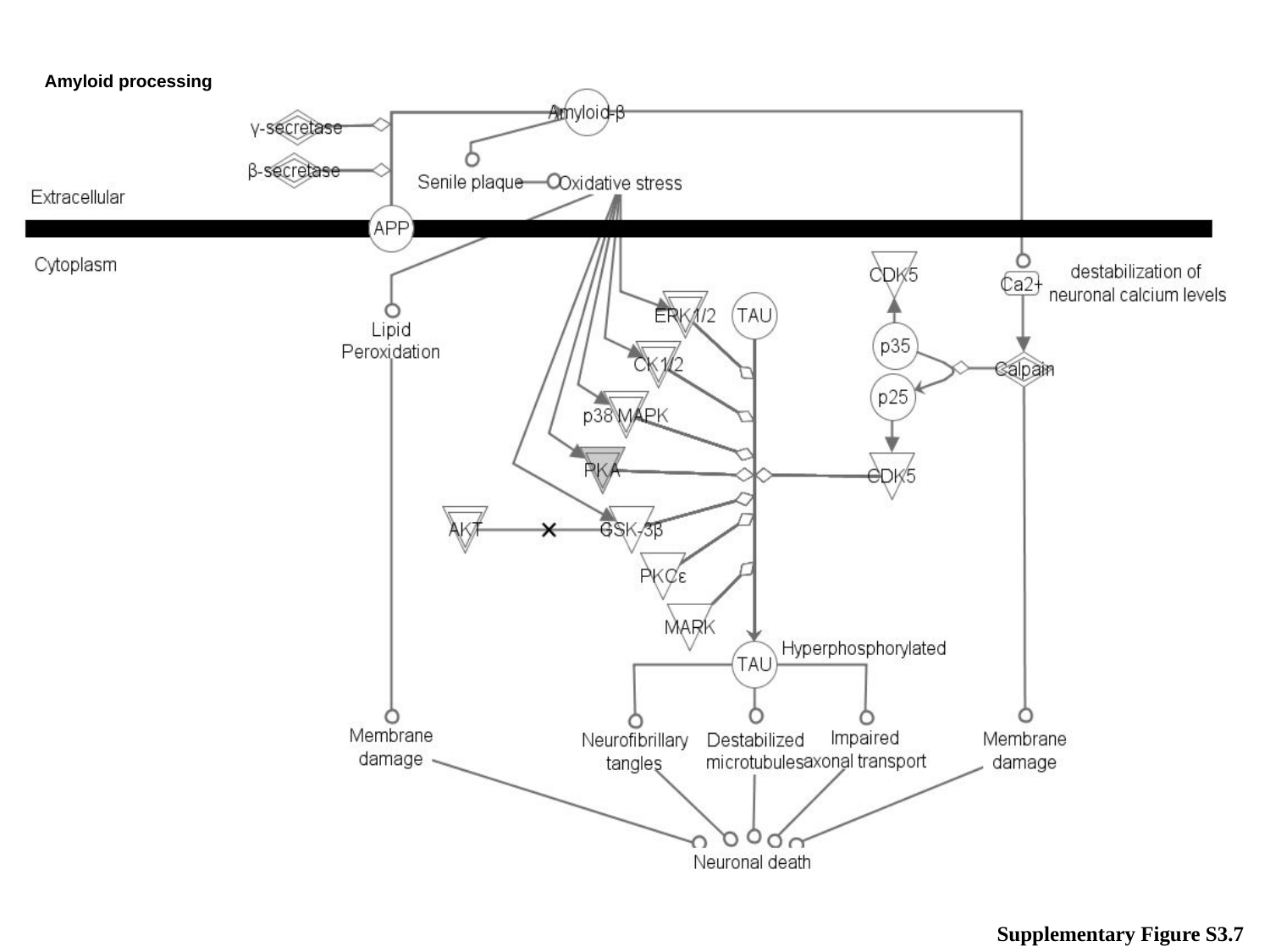

Amyloid processing
Supplementary Figure S3.7

## Slide 55
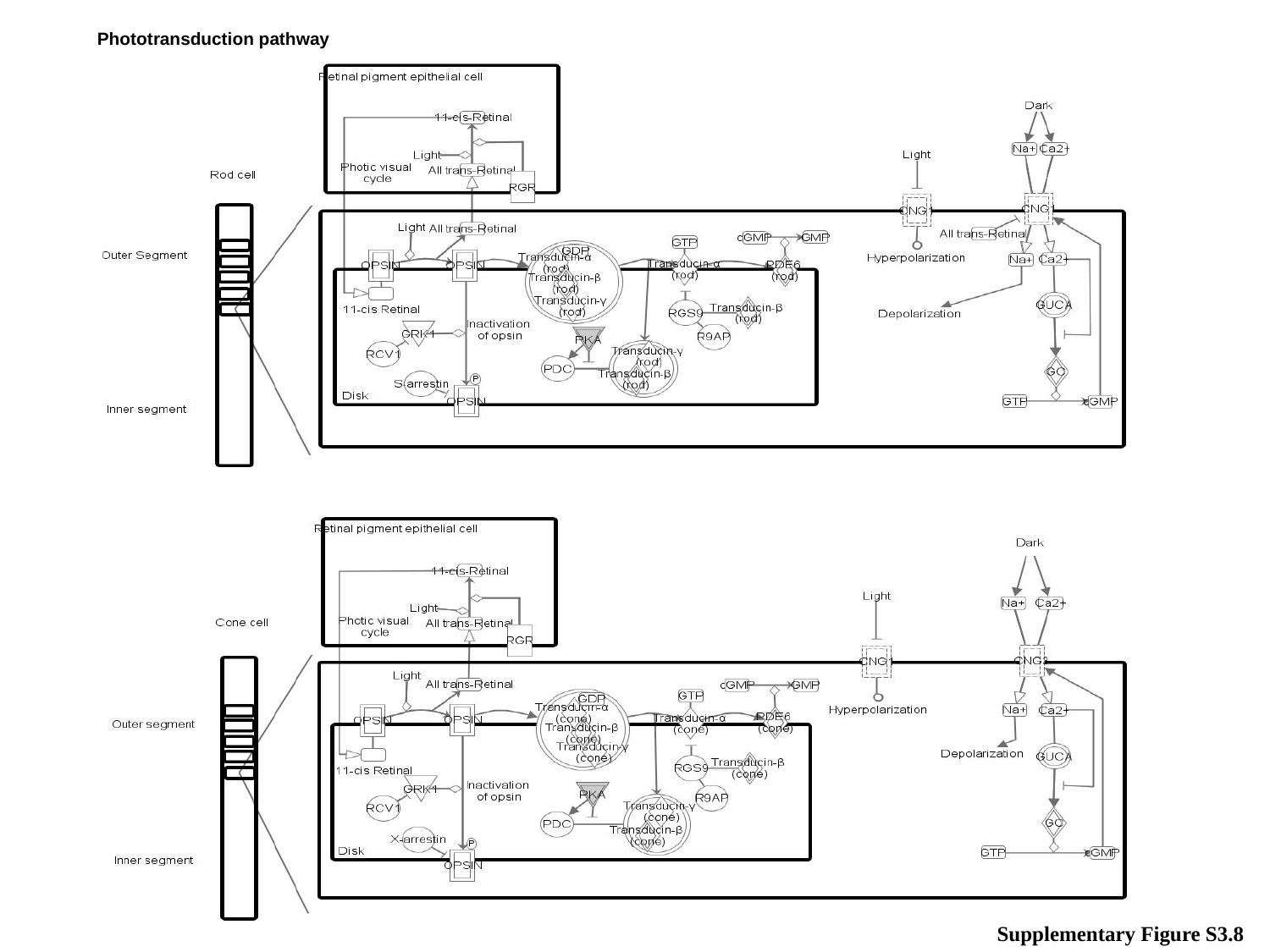

Phototransduction pathway
Supplementary Figure S3.8

## Slide 56
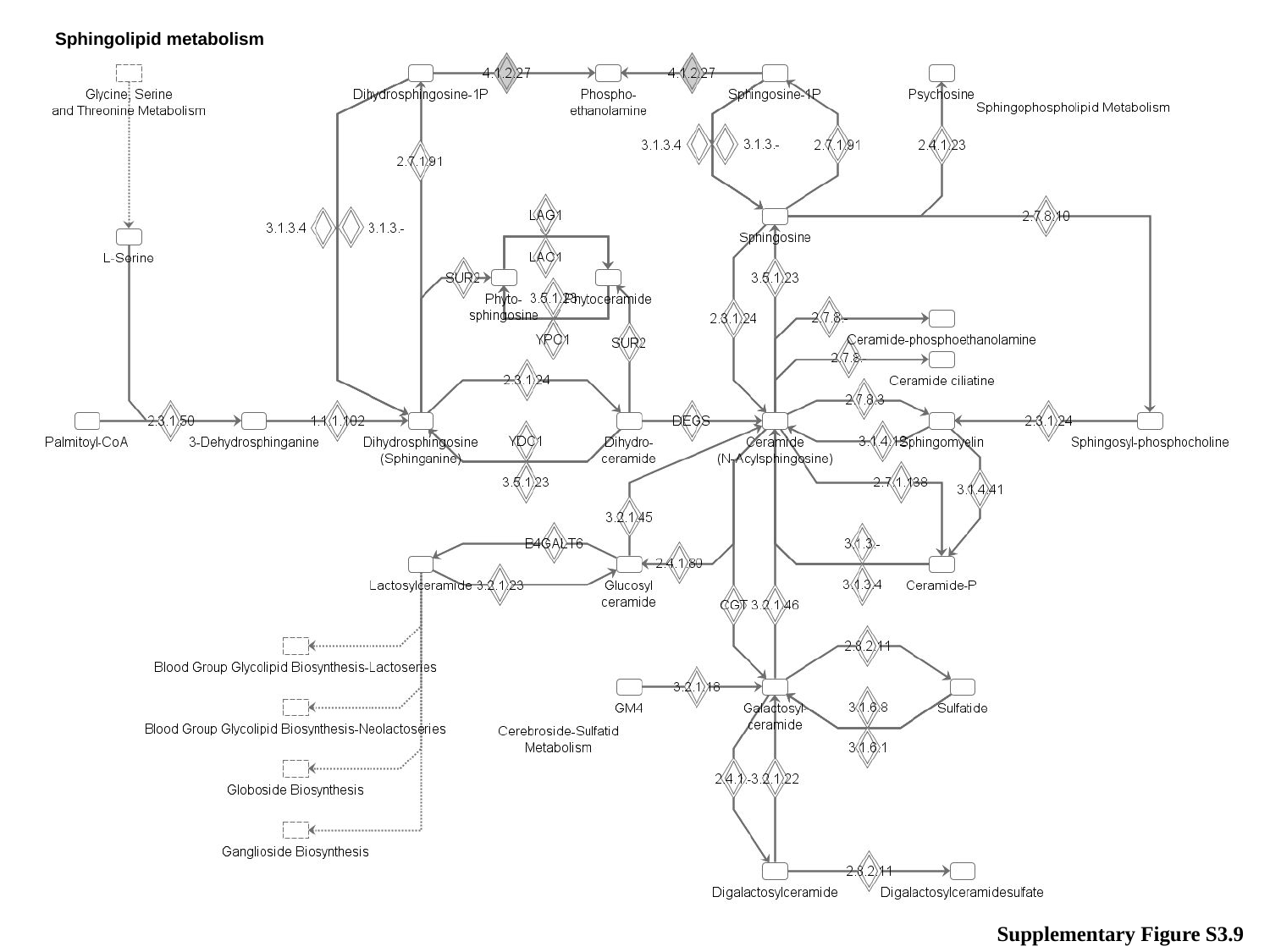

Sphingolipid metabolism
Supplementary Figure S3.9

## Slide 57
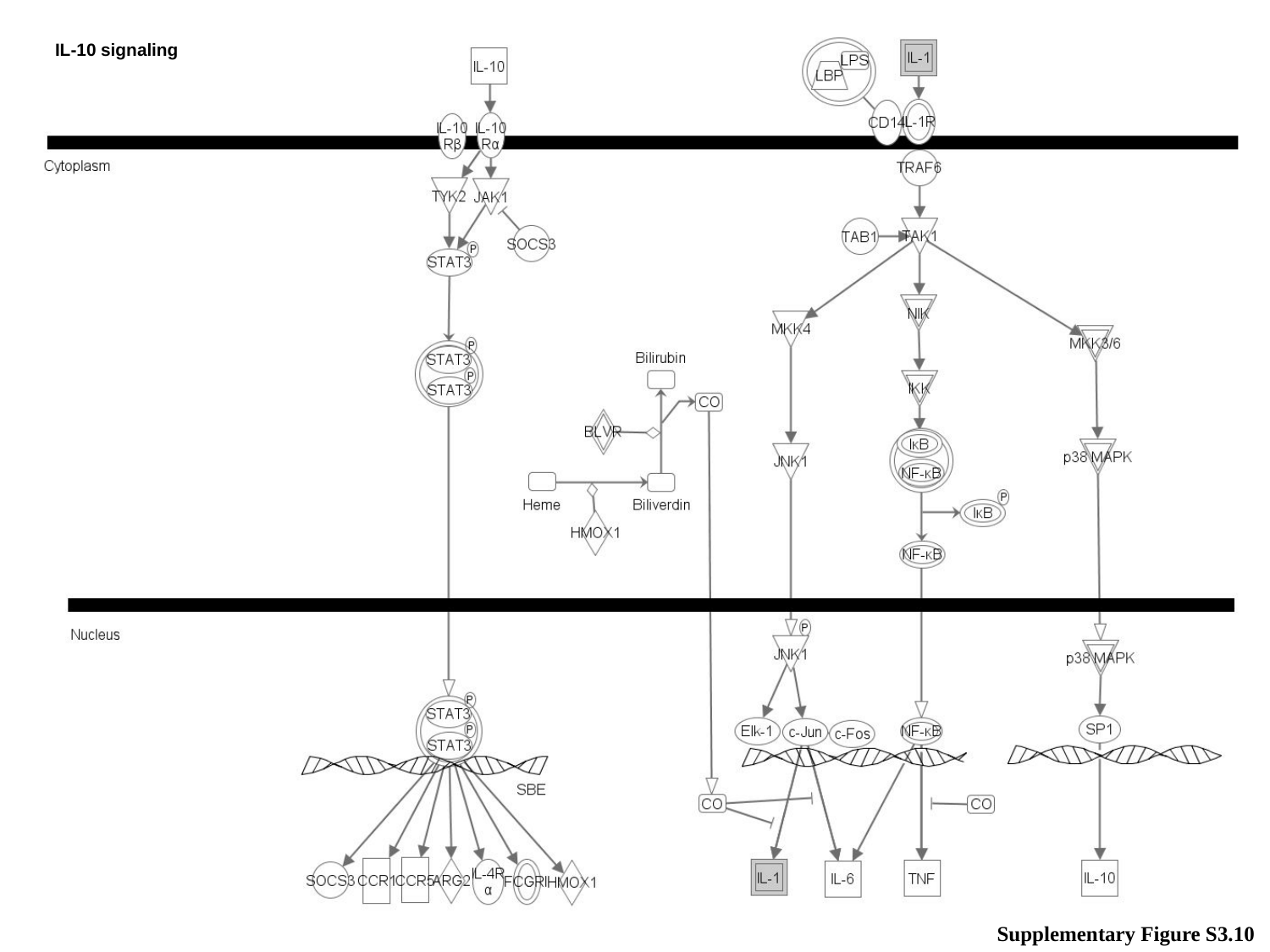

IL-10 signaling
Supplementary Figure S3.10

## Slide 58
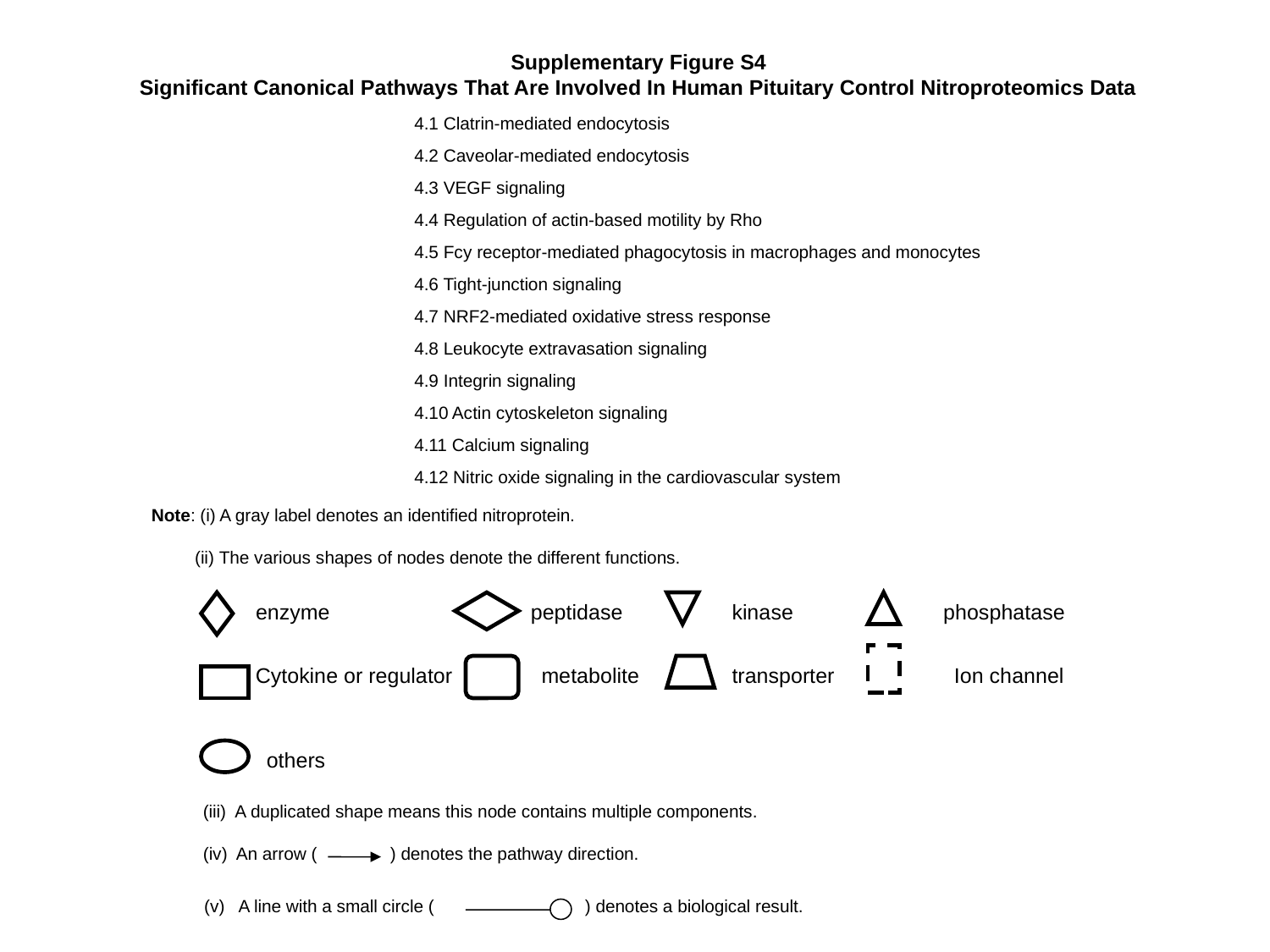

Supplementary Figure S4
Significant Canonical Pathways That Are Involved In Human Pituitary Control Nitroproteomics Data
4.1 Clatrin-mediated endocytosis
4.2 Caveolar-mediated endocytosis
4.3 VEGF signaling
4.4 Regulation of actin-based motility by Rho
4.5 Fcy receptor-mediated phagocytosis in macrophages and monocytes
4.6 Tight-junction signaling
4.7 NRF2-mediated oxidative stress response
4.8 Leukocyte extravasation signaling
4.9 Integrin signaling
4.10 Actin cytoskeleton signaling
4.11 Calcium signaling
4.12 Nitric oxide signaling in the cardiovascular system
Note: (i) A gray label denotes an identified nitroprotein.
 (ii) The various shapes of nodes denote the different functions.
enzyme
peptidase
kinase
phosphatase
Cytokine or regulator
metabolite
transporter
Ion channel
others
(iii) A duplicated shape means this node contains multiple components.
(v) A line with a small circle ( ) denotes a biological result.
(iv) An arrow ( ) denotes the pathway direction.

## Slide 59
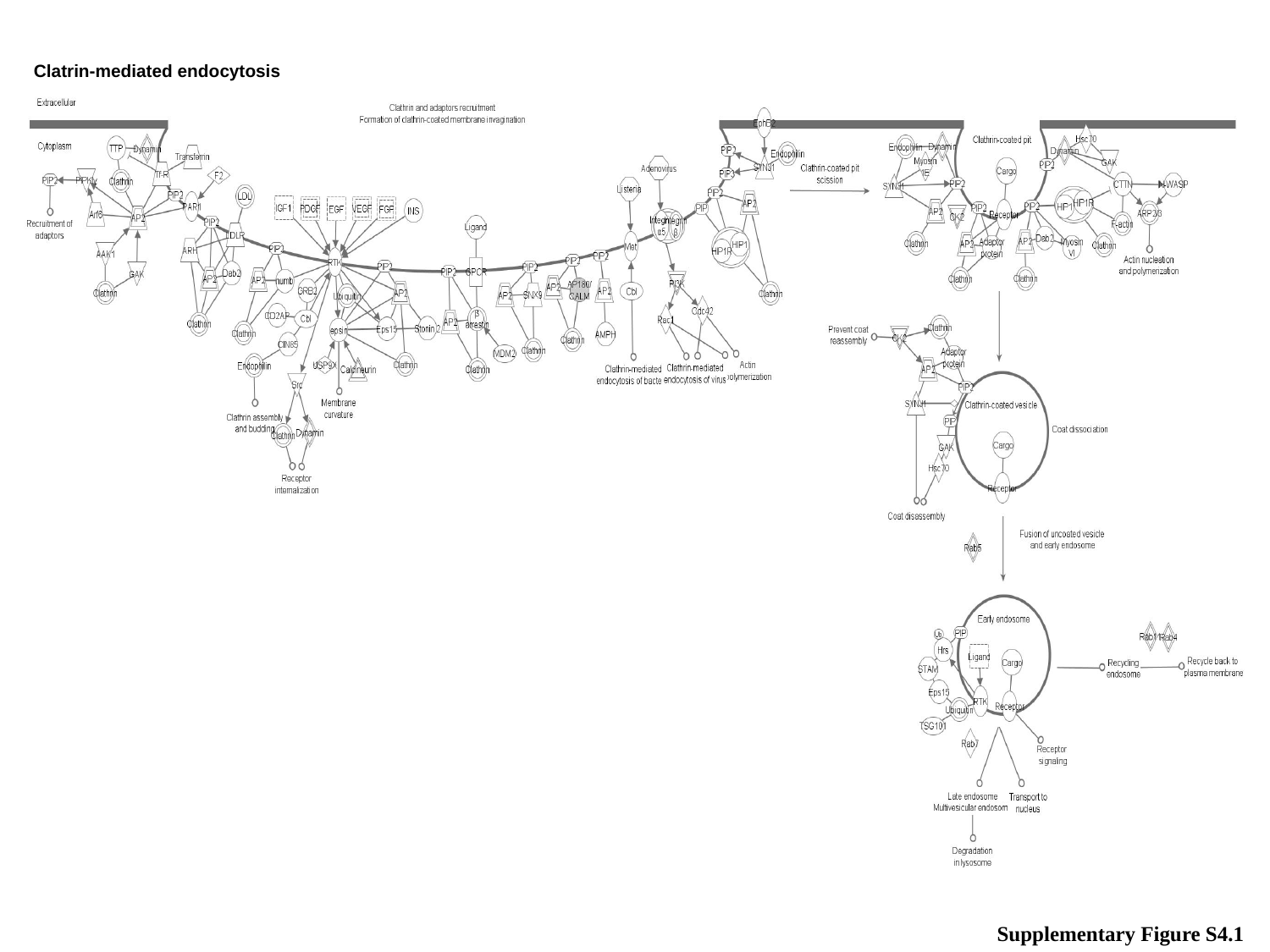

Clatrin-mediated endocytosis
Supplementary Figure S4.1

## Slide 60
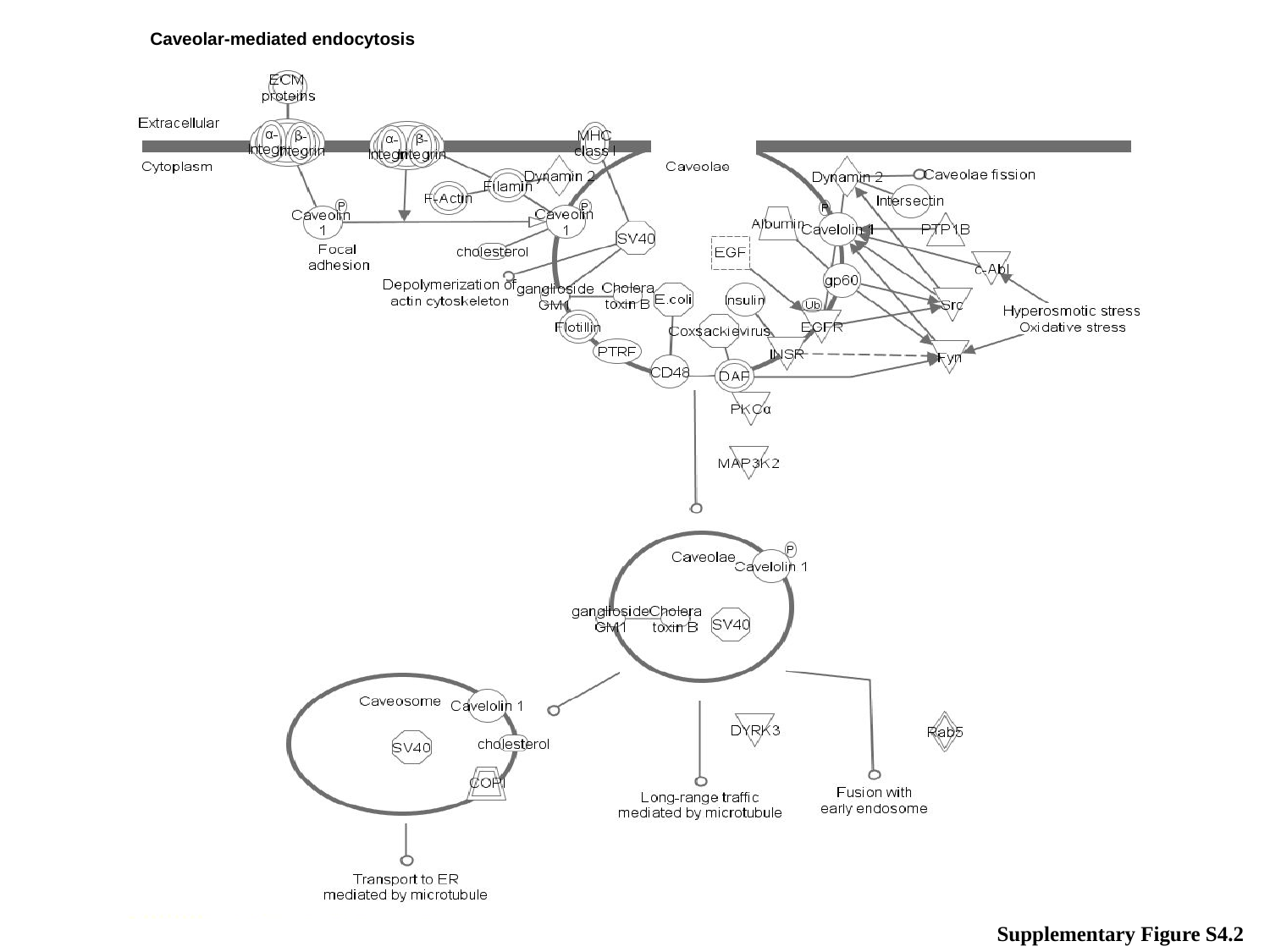

Caveolar-mediated endocytosis
Supplementary Figure S4.2

## Slide 61
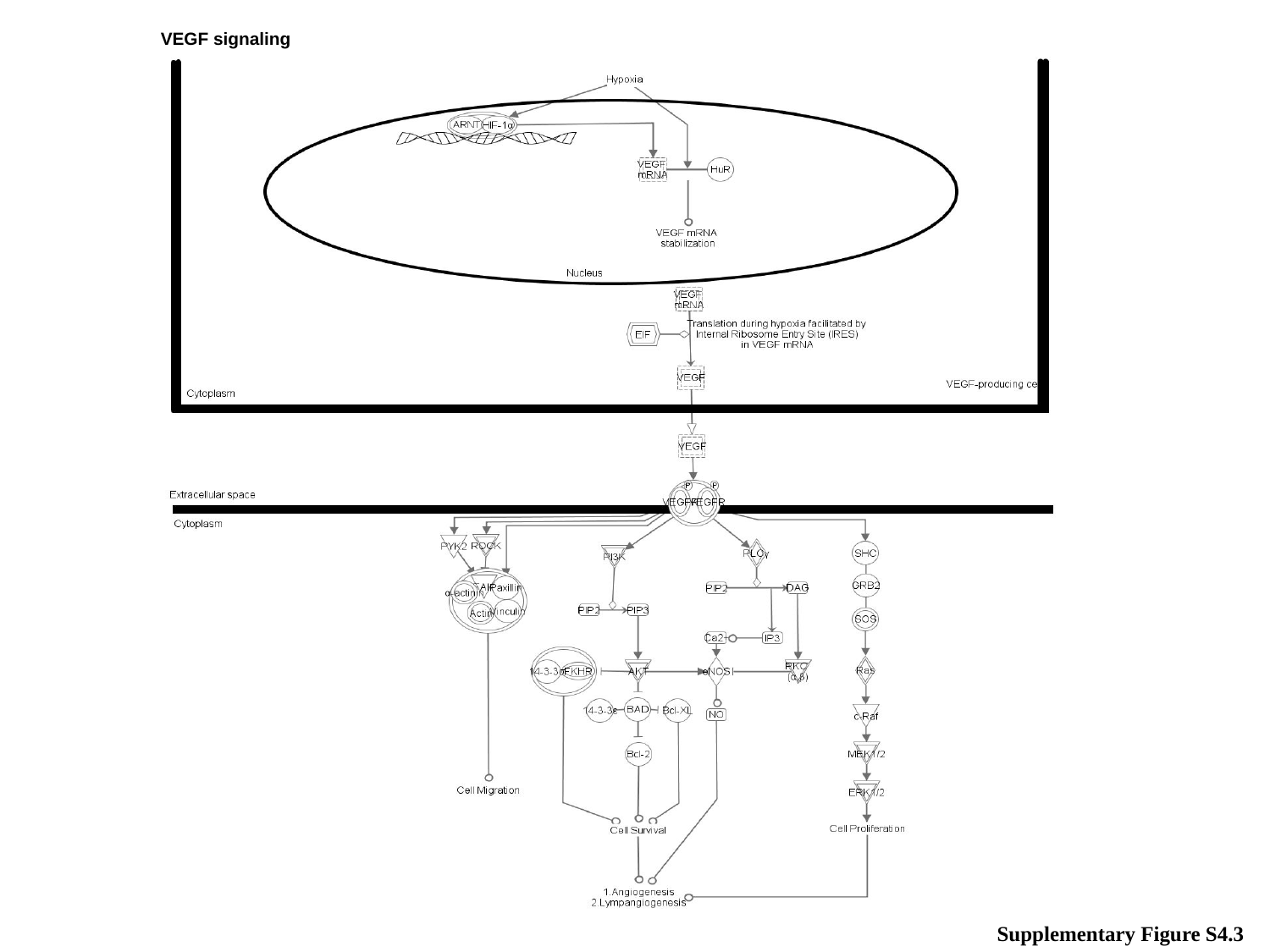

VEGF signaling
Supplementary Figure S4.3

## Slide 62
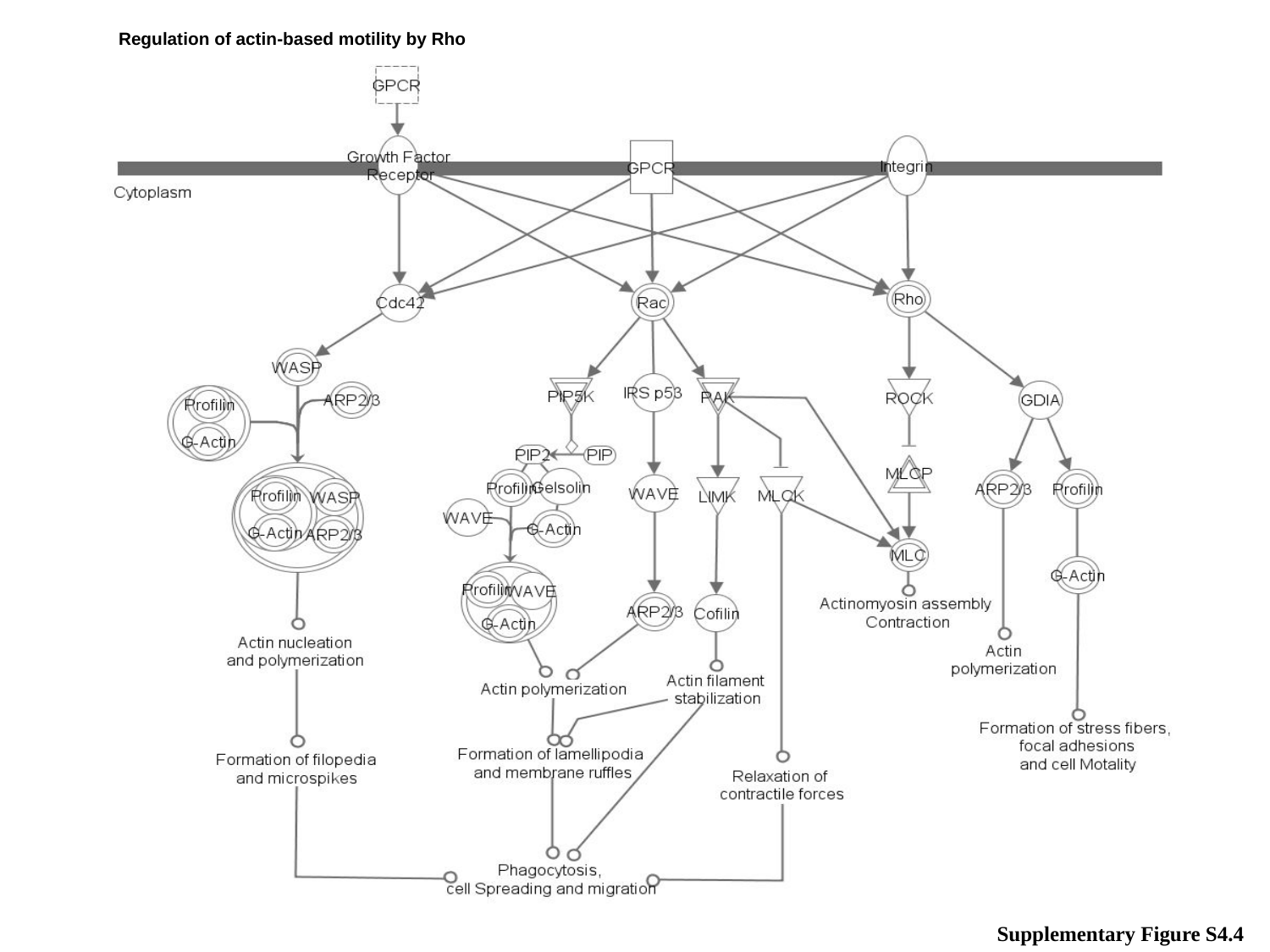

Regulation of actin-based motility by Rho
Supplementary Figure S4.4

## Slide 63
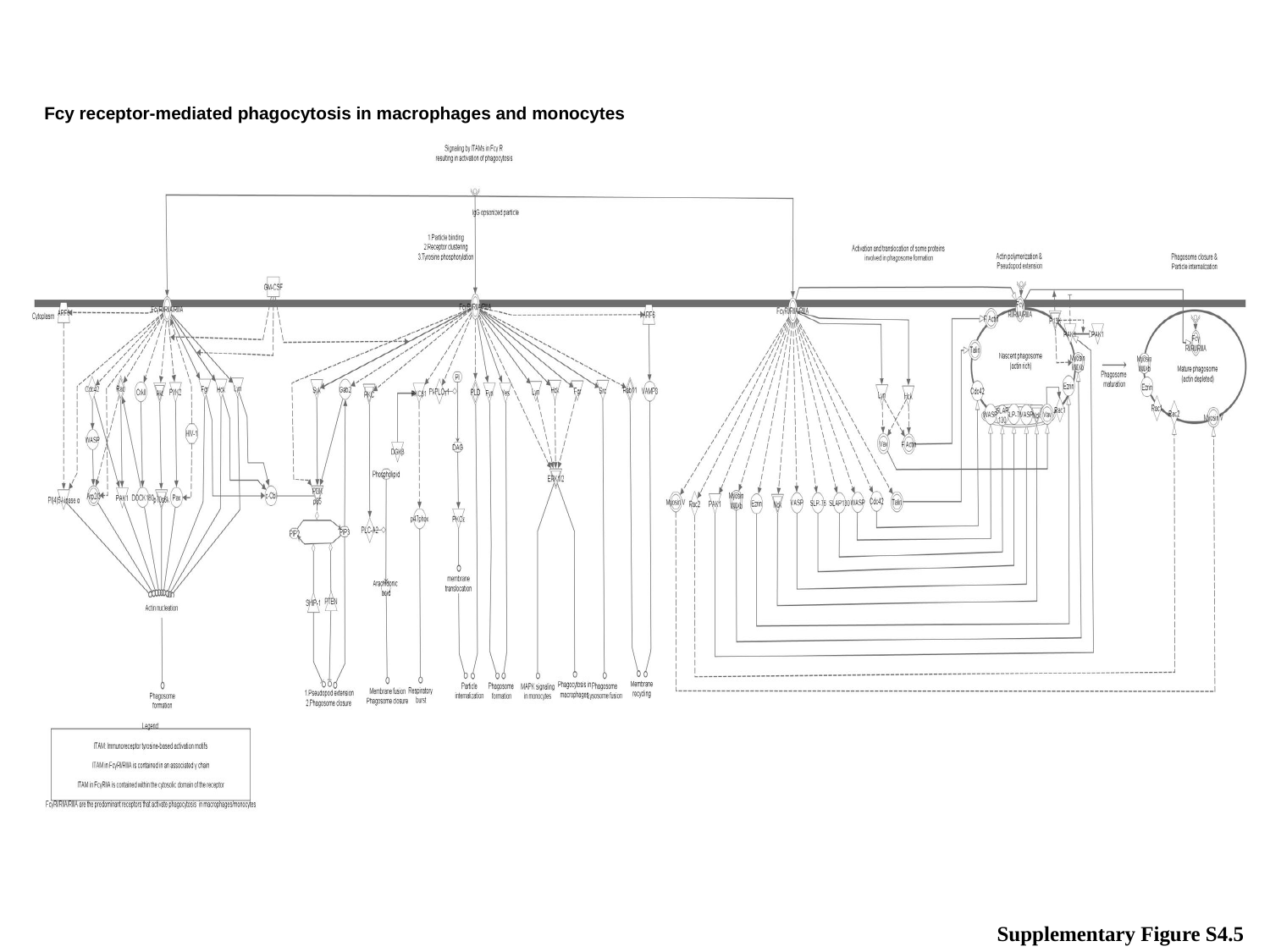

Fcy receptor-mediated phagocytosis in macrophages and monocytes
Supplementary Figure S4.5

## Slide 64
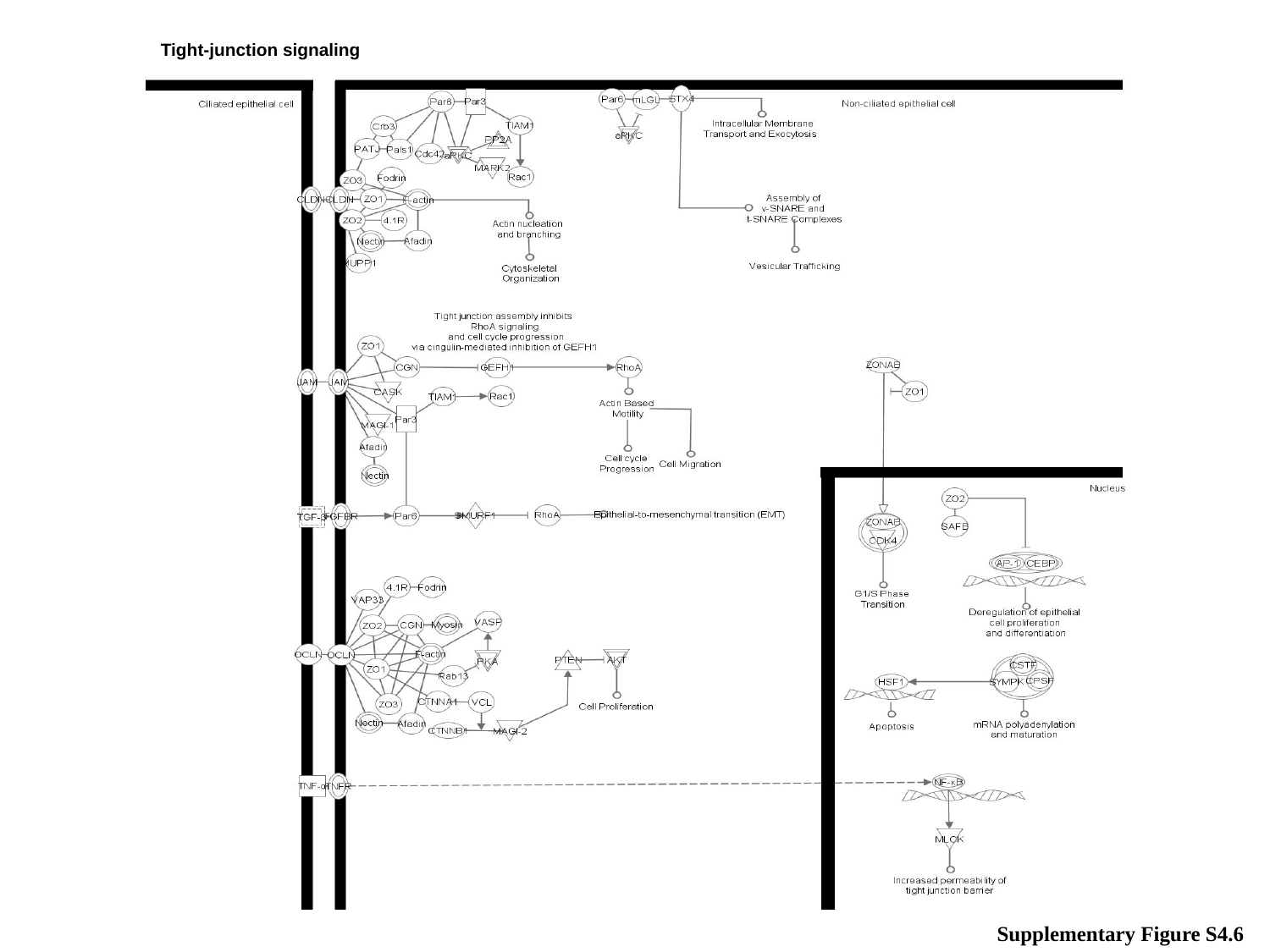

Tight-junction signaling
Supplementary Figure S4.6

## Slide 65
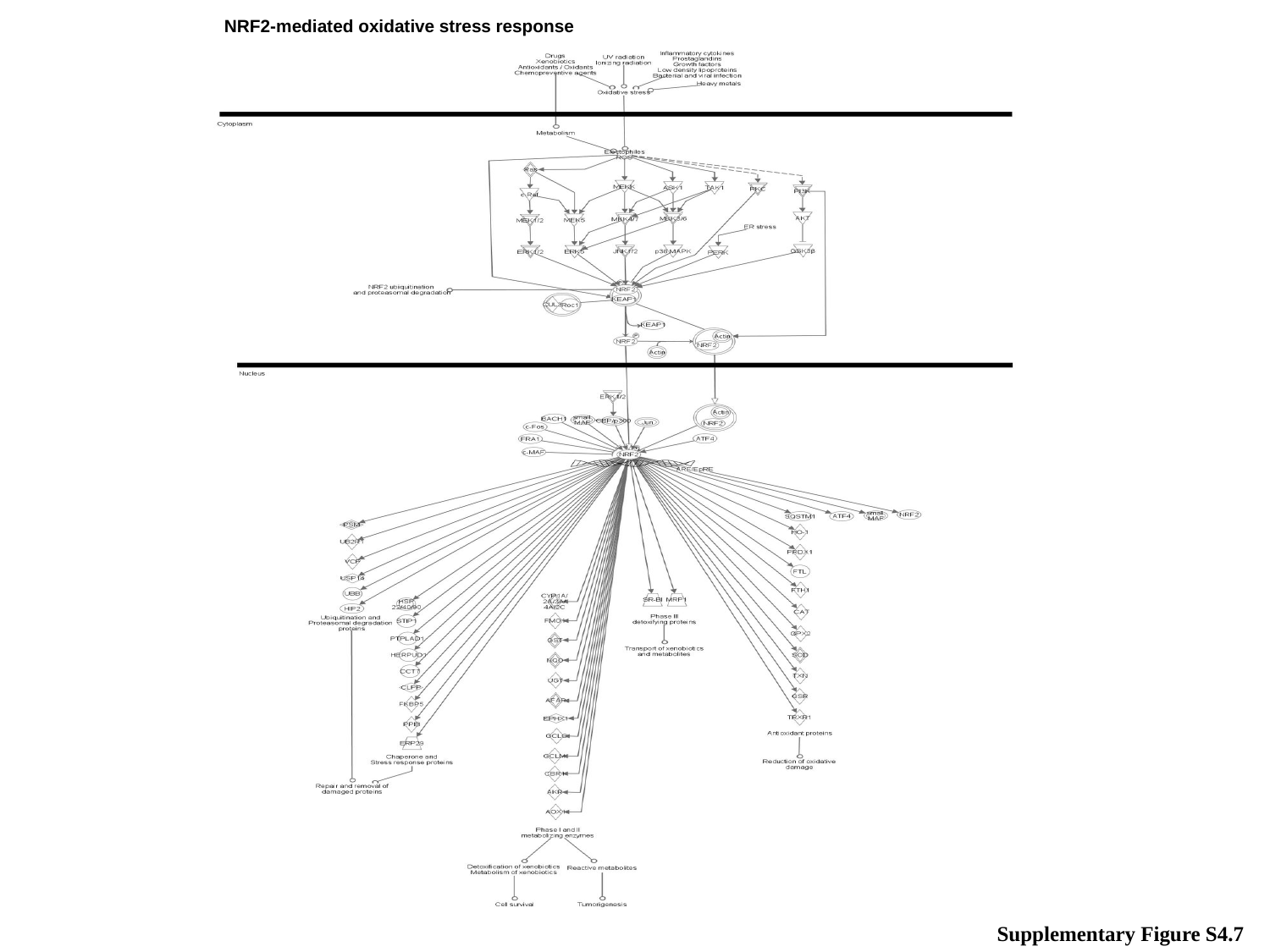

NRF2-mediated oxidative stress response
Supplementary Figure S4.7

## Slide 66
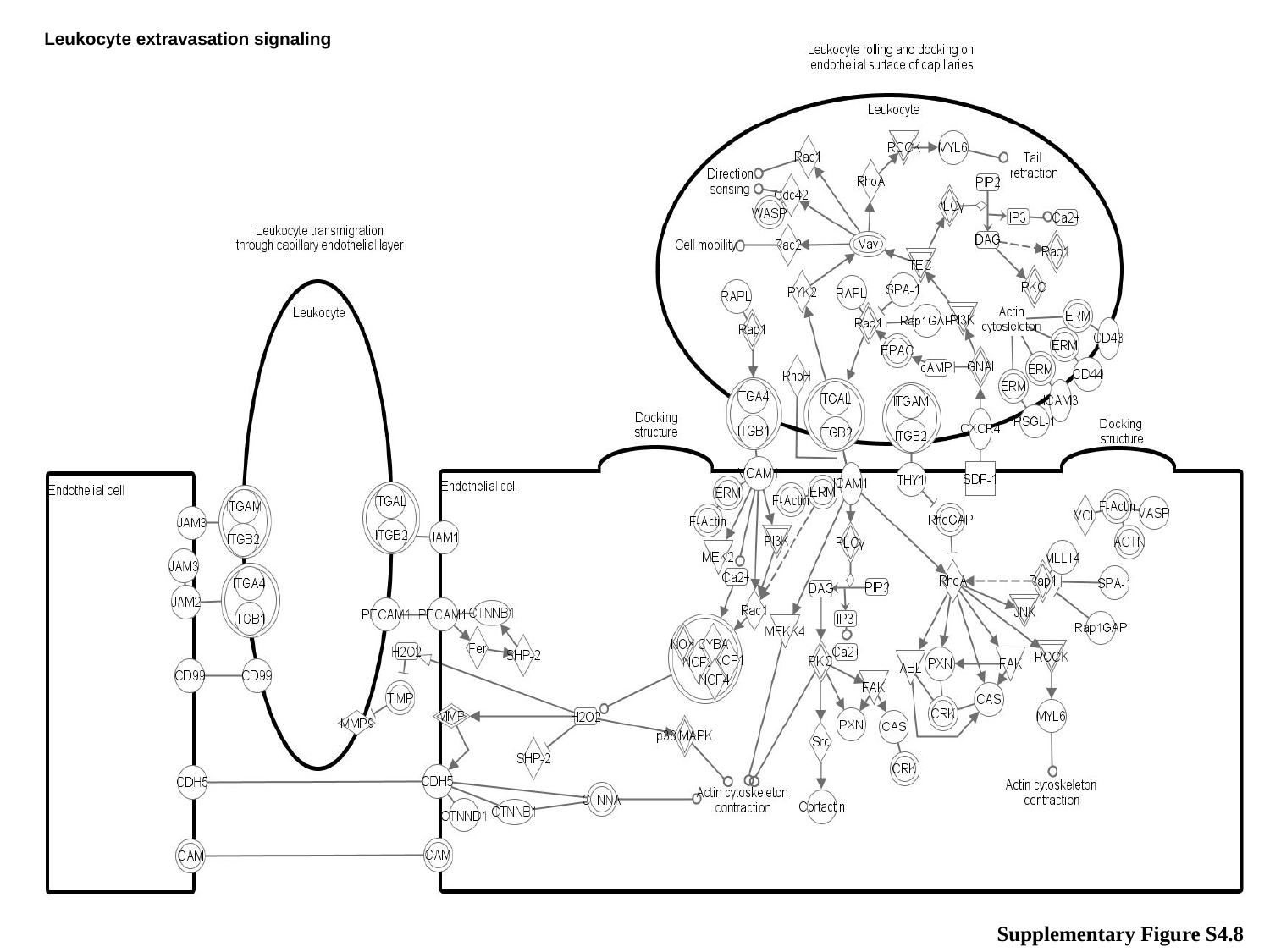

Leukocyte extravasation signaling
Supplementary Figure S4.8

## Slide 67
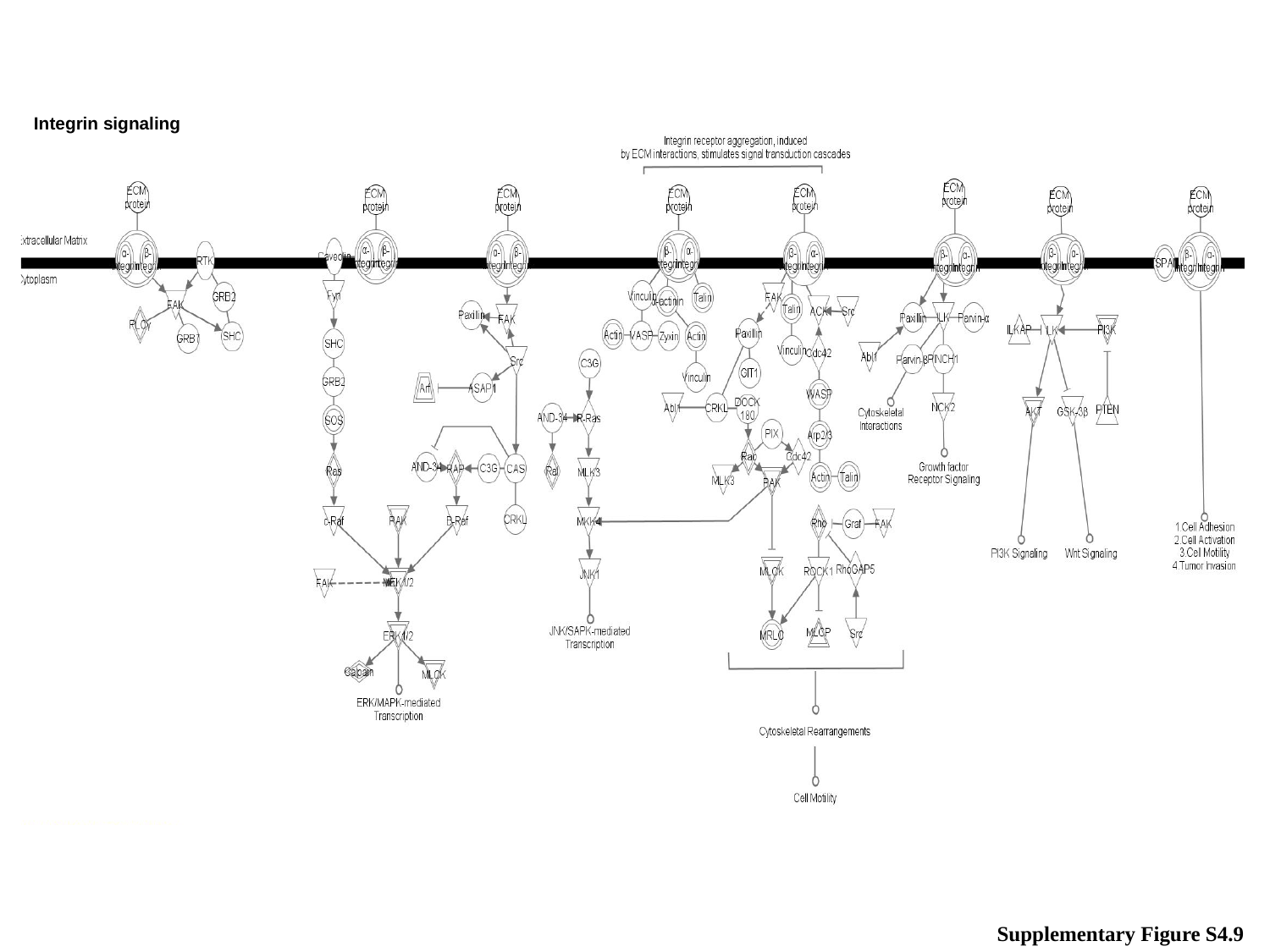

Integrin signaling
Supplementary Figure S4.9

## Slide 68
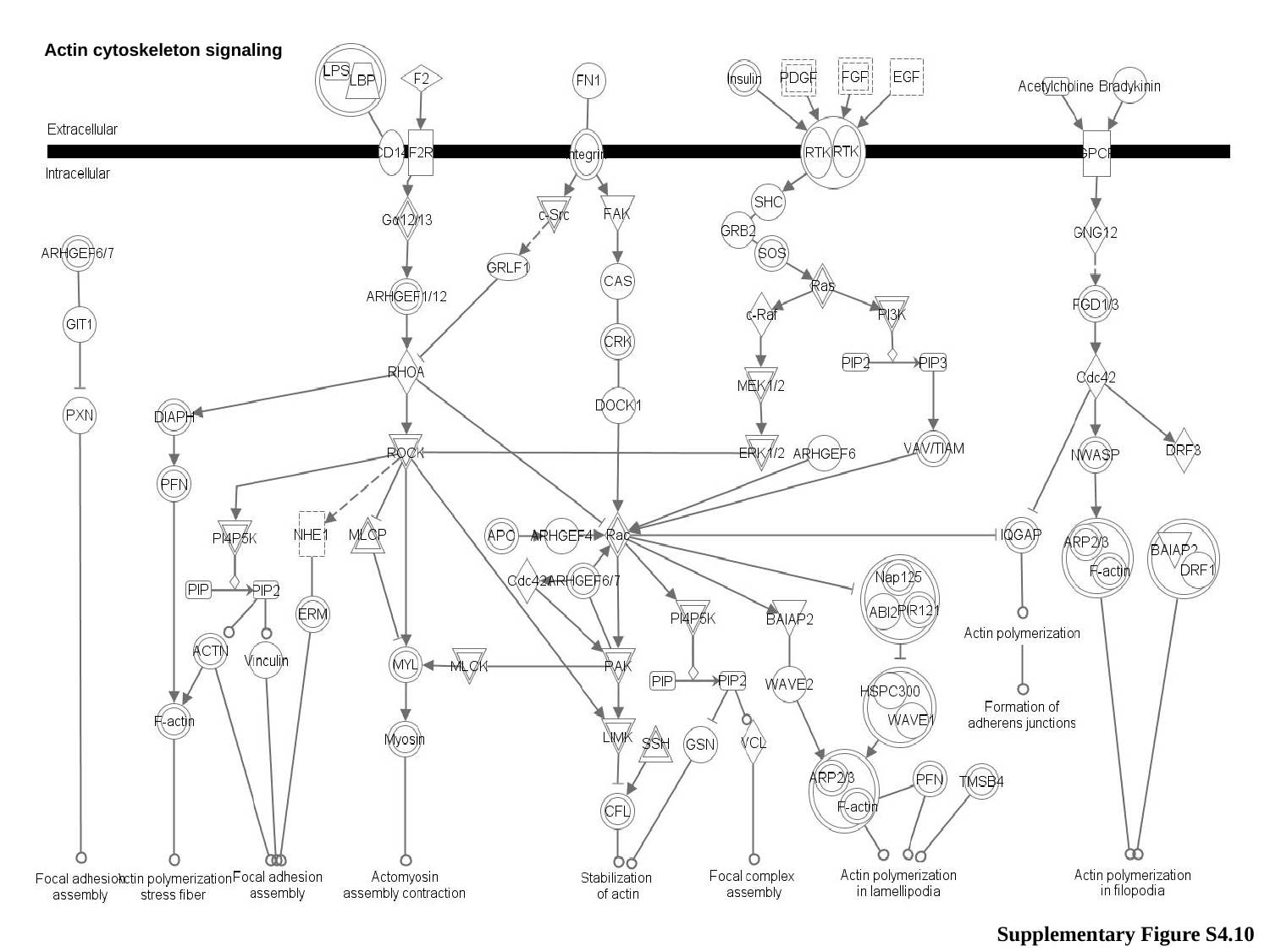

Actin cytoskeleton signaling
Supplementary Figure S4.10

## Slide 69
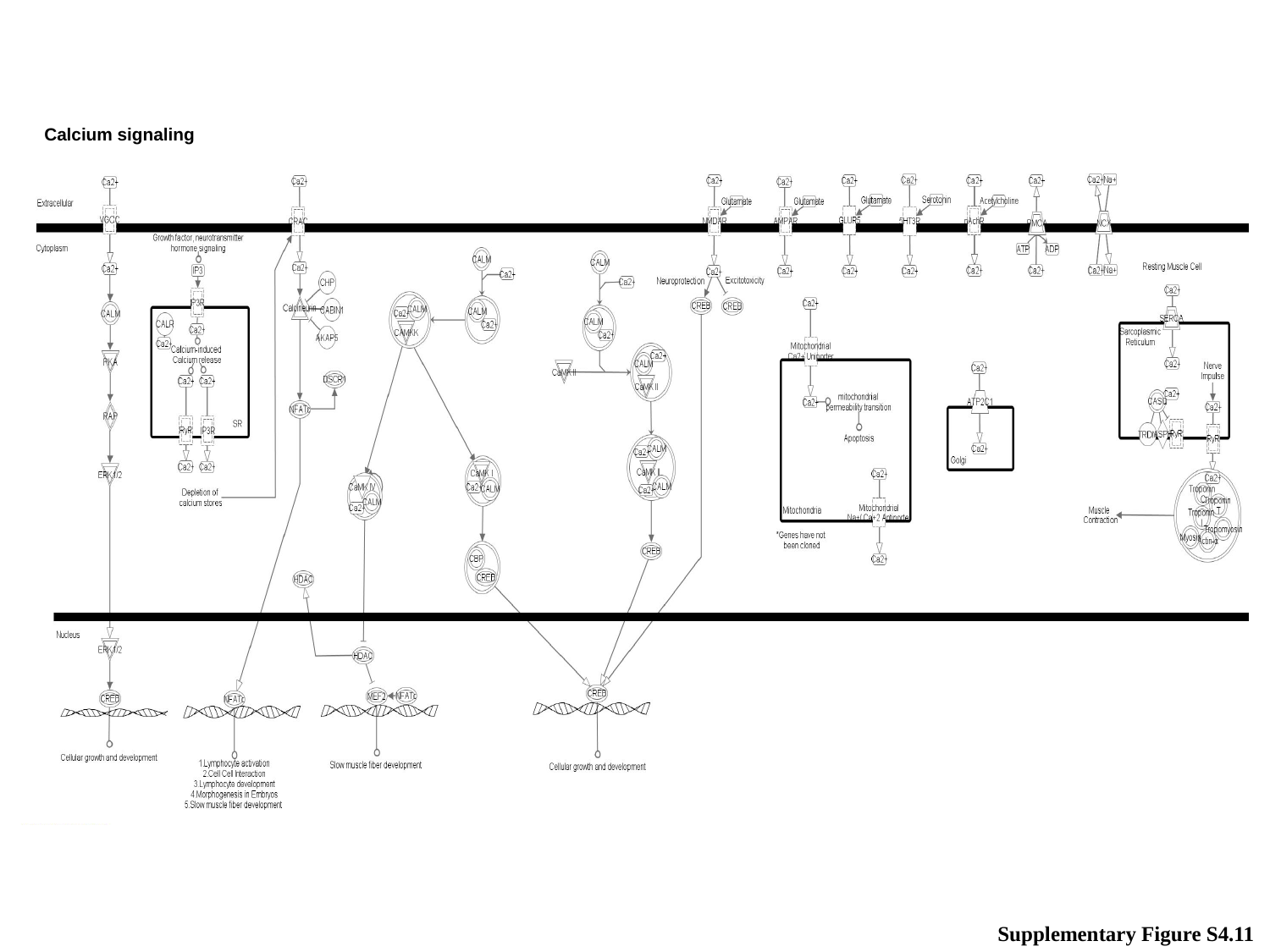

Calcium signaling
Supplementary Figure S4.11

## Slide 70
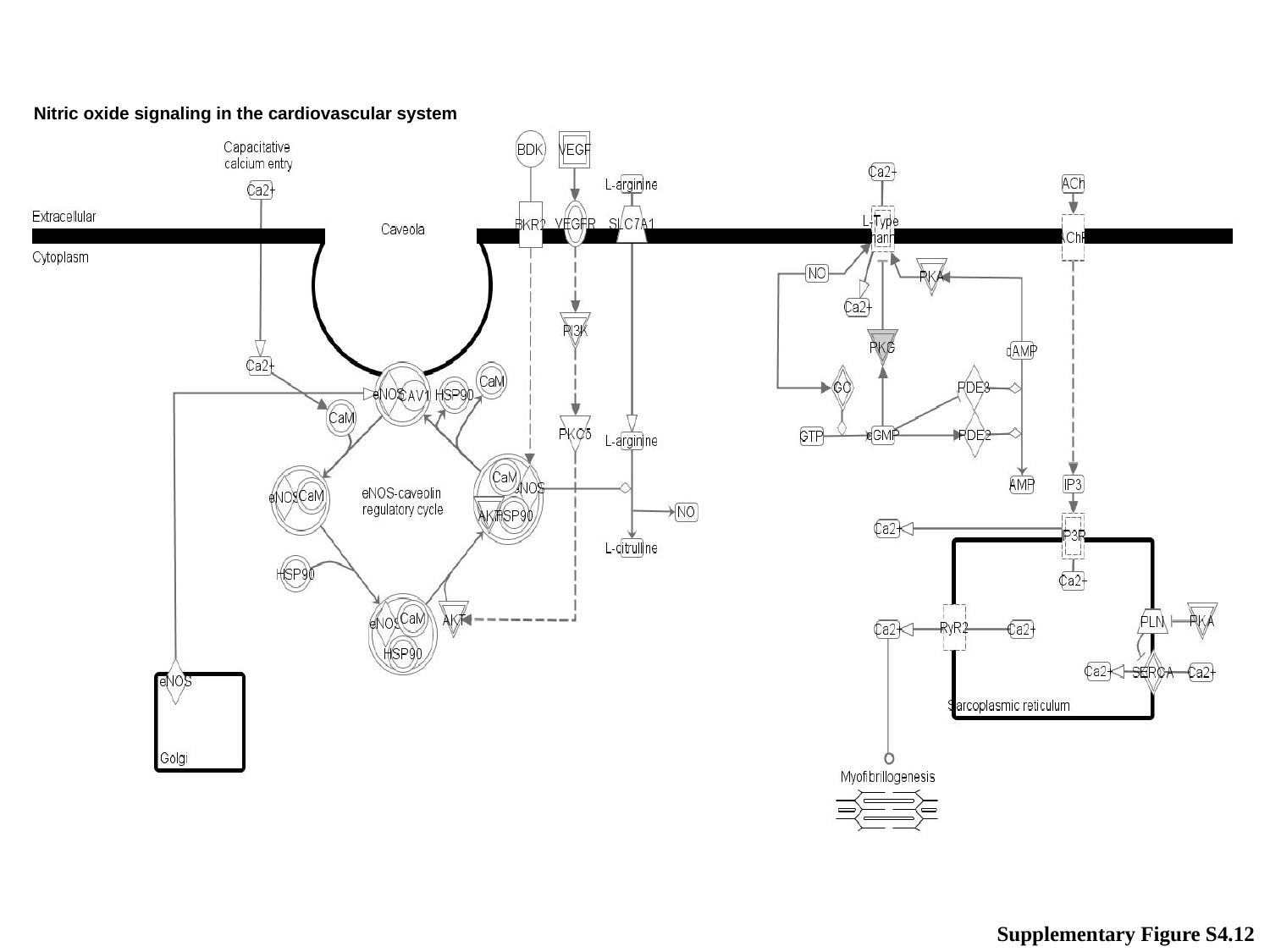

Nitric oxide signaling in the cardiovascular system
Supplementary Figure S4.12
